# Inference for binomial probability based on dependent Bernoulli random variables with applications to meta‐analysis and group level studies

**DOI:** 10.1002/bimj.201500115

**Published:** 2016-05-18

**Authors:** Ilyas Bakbergenuly, Elena Kulinskaya, Stephan Morgenthaler

**Affiliations:** ^1^ School of Computing SciencesUniversity of East AngliaNorwich NR4 7TJUK; ^2^ École polytechnique fédérale de Lausanne (EPFL)Station 81015 LausanneSwitzerland

**Keywords:** Intracluster correlation, Meta‐analysis, Overdispersion, Random effects, Transformation bias

## Abstract

We study bias arising as a result of nonlinear transformations of random variables in random or mixed effects models and its effect on inference in group‐level studies or in meta‐analysis. The findings are illustrated on the example of overdispersed binomial distributions, where we demonstrate considerable biases arising from standard log‐odds and arcsine transformations of the estimated probability $\hat{p}$, both for single‐group studies and in combining results from several groups or studies in meta‐analysis. Our simulations confirm that these biases are linear in ρ, for small values of ρ, the intracluster correlation coefficient. These biases do not depend on the sample sizes or the number of studies *K* in a meta‐analysis and result in abysmal coverage of the combined effect for large *K*. We also propose bias‐correction for the arcsine transformation. Our simulations demonstrate that this bias‐correction works well for small values of the intraclass correlation. The methods are applied to two examples of meta‐analyses of prevalence.

## 1 Introduction

The main focus of this paper is the bias that arises as a result of transformations of random variables in random or mixed effects models and the deleterious effects of these biases on inference in group‐level studies, such as ecological studies in epidemiology or cluster‐randomized trials, and in meta‐analyses. For a single sample, the influential paper by Cox (1983) investigated transformations in some detail. Suppose that $X_{n}$ is an unbiased estimator based on a sample of size *n* for some real parameter θ and furthermore that we are interested in the estimator $f(X_{n})$ of the transformed parameter η=f(θ) for a nonlinear transformation f(·). The estimator $f(X_{n})$ will then exhibit a finite‐sample bias, but it retains consistency. If an unsuspected random effect is introduced, however, the estimator loses its consistency, because the bias is enlarged by overdispersion.

If the overdispersion is small and undetectable in the data, it may still severely affect the inference on transformed effects in a group‐level study or in a meta‐analysis. Both types of studies are increasingly popular in biomedical applications and aim to combine group‐level effects. In epidemiology, studies are often based on routinely collected administrative unit‐level data, such as prevalence or incidence of a disease or condition in a population. Cluster‐randomized trials are motivated by the convenience of group‐level treatment allocation. Meta‐analyses aim to combine evidence from the existing studies. Requisite statistical methods are essentially the same; they are based on random or mixed effects models, and are well established, especially so in meta‐analysis. Our findings apply both to the standard additive random effects model (REM) of meta‐analysis (Hedges and Olkin, 1985), and to the multiplicative version of REM (Kulinskaya and Olkin, 2014).

We illustrate our findings with the comparatively simple example of overdispersed binomial data, where overdispersion arises as a result of an intracluster correlation ρ between Bernoulli random variables in cluster‐randomized trials or within studies in meta‐analyses. The most general assumptions about dependent Bernoulli variables $\left\{X_{1},X_{2},...,X_{n}\right\}$ specify only the first two moments:

(1) $$\text{E}\left(X_{i}\right)=p_{i},\;\text{Var}\left(X_{i}\right)=p_{i}\left(1-p_{i}\right),\;\text{and}\;\rho_{ij}=\left(P\left(X_{i}=1,X_{j}=1\right)-p_{i}p_{j}\right)/\sqrt{p_{i}q_{i}p_{j}q_{j}},$$

where $p_{i}$ is the probability of success or prevalence of a condition, $q_{i}=1-p_{i}$ and $\rho_{ij}=\text{corr}\left(X_{i},X_{j}\right)$ is the correlation of $X_{i}$ and $X_{j}$, i≠j. As Prentice (1988) shows, the correlation values are restricted to the interval

(2) $$\left(\text{max}\left(-\sqrt{\frac{p_{i}p_{j}}{q_{i}q_{j}}},-\sqrt{\frac{q_{i}q_{j}}{p_{i}p_{j}}}\right),\text{min}\left(\sqrt{\frac{p_{i}q_{j}}{q_{i}p_{j}}},\sqrt{\frac{q_{i}p_{j}}{p_{i}q_{j}}}\right)\right)\;.$$

This bound need not apply if the interest lies in an overdispersed binomial distribution, since overdispersed binomial random variables are not restricted to sums of dependent Bernoullis. We concentrate on the simplest model with constant probabilities and correlations, that is, $p_{i}=p$ and $\rho_{ij}=\rho$. The restriction on ρ then reduces to max(−p/q,−q/p)<ρ<1. Further restrictions may arise from particular data‐generating distributions and/or latent variables. There exist a variety of methods to generate dependent Bernoulli variables; three of them are used in our simulations: the Gaussian copula (GC) method of Emrich and Piedmonte (1991), the Lunn and Davies (1998) method and the beta‐binomial distribution; see Web Appendix for details.

The intraclass correlation ρ (ICC) is typically small in bio‐medical applications, and the number of clusters *K* is moderate to large in group level studies. Clustering is mostly due to the same healthcare provider (health practitioner, general practice, clinic, etc.). Gulliford et al. (2005) analyzed the data on 188 ICCs obtained from the General Practice Research Database (GPRD) for variation of outcomes and performance between United Kingdom general practices and 136 results from a Health Technology Assessment (HTA) review for a range of outcomes in community and health services settings. In the GPRD, the median prevalence *p* was 0.13 (interquartile range IQR 0.284 − 0.035), and median ICC was 0.051 (IQR 0.094 − 0.011). In the HTA review, the median prevalence was 0.065 (IQR 0.207 − 0.004), and median ICC, 0.006 (IQR 0.036 − 0.0003). Similarly, Littenberg and MacLean (2006) calculated the ICC for 62 binary variables, measured as part of the Vermont Diabetes Information System, a cluster‐randomized study of adults with diabetes from 73 primary care practices in Vermont, USA and surrounding areas. The median ICC was 0.022; IQR (0.040 − 0.006). Prevalence of some comorbidities and complications and certain aspects of quality of life varied much more among patients with only small correlation within practices (ICC<0.001). Eldridge et al. (2004) provided a systematic review of 152 published and 47 unpublished cluster‐randomized trials in primary health care, published between 1997 and 2000. The median number of clusters in the published trials was 29, and in the unpublished trials it was 32. In meta‐amalysis, though, the number of studies may be quite small.

We illustrate our general findings in examples of biases from arcsine and logit (log‐odds) transformations in single studies and in meta‐analysis, concentrating on small values of the ICC, viz. ρ<0.1. These transformations are very popular in the analysis of binomial proportions (Kulinskaya et al., 2008, Ch.18), and they are also used in other popular effect measures for binary data, such as the log‐odds‐ratios or the differences of arcsine‐transformed proportions (Hedges and Olkin, 1985; Rücker et al., 2009).

The structure of this paper is as follows. The transformation bias is introduced in Section 2. Section 3 explores the consequences of the transformation bias when combining results in meta‐analysis or (equivalently) in analysis of group‐level studies, and Section 4 applies our methodology to two examples of meta‐analyses of prevalence of a disease or a condition. Final comments and discussion are given in Section 5. Additional material, including the methods for generating Bernoulli variables and detailed simulation results, are provided in Web Appendix.

## 2 Transformation bias

### 2.1 Theoretical derivation of transformation bias

Consider a real‐valued statistic *X*, an estimator of a real parameter based on a sample of size *n*. Let $g_{\tau }\left(x\right)$ denote the density of *X*, where τ≥0 is an overdispersion parameter. When τ=0, we have the null or “fixed effect” model with density $g\left(x\right)=g_{0}\left(x\right)$, and for τ>0 we have a “random effects” model (REM). Denote the expected value and the central moments of *X* by $x_{\tau }={\text{E}}_{\tau }\left(X\right)$ and $\mu_{j}\left(\tau \right)={\text{E}}_{\tau }\left({\left(X-x_{\tau }\right)}^{j}\right),\;j>1$, respectively. We assume that all moments $\mu_{j}\left(\tau \right),\;j>1$ of *X* exist for values of τ close to zero, and that the variance is of order O(1/n) and the higher moments are o(1/n). This setting is similar to that of Cox (1983). Let f(X) be a transformation of *X* such that derivatives of all orders exist. Think of *X* as an estimator of *x*_0_ and of τ as an effect that is not part of the model used by the statistician. The expected value of the transformed variable is

(3) $${\text{E}}_{\tau }\left(f\left(X\right)\right)=\int f\left(x\right)g_{\tau }\left(x\right)dx=f\left(x_{0}\right)+\sum_{j=1}^{\infty }\frac{1}{j!}\frac{d^{j}f\left(x\right)}{dx^{j}}{|}_{x_{0}}{\text{E}}_{\tau }\left({\left(X-x_{0}\right)}^{j}\right)\;.$$

The first two terms of this series collect all the terms up to order O(1/n) at the model τ=0

(4) $${\text{E}}_{\tau }\left(f\left(X\right)\right)-f\left(x_{0}\right)=\frac{df(x)}{dx}{|}_{x_{0}}{\text{E}}_{\tau }\left(X-x_{0}\right)+\frac{1}{2}\frac{d^{2}f\left(x\right)}{dx^{2}}{|}_{x_{0}}{\text{E}}_{\tau }{\left(X-x_{0}\right)}^{2}+\mathrm{remainder}.$$

The left‐hand side is the bias of f(X) as an estimator of $f(x_{0})$. The first term on the right‐hand side measures the influence of the bias, and the second term the one of the mean squared error of *X* introduced by the “random effects” model. The formula shows that f(X) is an unbiased estimator of $f(x_{0})$ to order O(1/n) only if *X* remains unbiased even under the REM, that is $x_{\tau }=x_{0}$, and if furthermore the transformation is linear.

The bias to order O(1/n) can be calculated directly if the first two moments of *X* under the density $g_{\tau }\left(x\right)$ are known:

$$f^{\prime }\left(x\right){|}_{x_{0}}\left(x_{\tau }-x_{0}\right)+\frac{1}{2}f^{\prime \prime }\left(x\right){|}_{x_{0}}\left(\mu_{2}\left(\tau \right)+{\left(x_{\tau }-x_{0}\right)}^{2}\right)\;.$$

Further, setting τ=0 shows that for a nonlinear transformation, f(X) is in general not an unbiased estimator of $f(x_{0})$. Now consider a similar expansion for the variance. Using (3) for $f^{2}\left(X\right)$, and subtracting the relevant order terms from $\left[{\text{E}}_{\tau }{\left[f\left(X\right)\right]}^{2}\right]$, we obtain

(5) $$\begin{array}{ccc} {\text{Var}}_{\tau }\left(f\left(X\right)\right) & = & {\text{Var}}_{0}\left(f\left(X\right)\right)+{\left[f^{\prime }\left(x\right){|}_{x_{0}}\right]}^{2}\left[\mu_{2}\left(\tau \right)-\mu_{2}\left(0\right)\right]+\\ & & +\;2\left[f^{\prime }\left(x\right){|}_{x_{0}}\right]\left[f\left(x_{0}\right)-{\text{E}}_{0}\left(f\left(X\right)\right)\right]\left[x_{\tau }-x_{0}\right]+\\ & & +\;\frac{1}{2}\left[f^{\prime \prime }\left(x\right){|}_{x_{0}}\right]\left[f\left(x_{0}\right)-{\text{E}}_{0}\left(f\left(X\right)\right)\right]\left[\mu_{2}\left(\tau \right)-\mu_{2}\left(0\right)\right]+\\ & & -\;\left[f^{\prime \prime }\left(x\right){|}_{x_{0}}\right]{\left[x_{\tau }-x_{0}\right]}^{2}+\cdots . \end{array}$$

If τ is of order $O(1/\sqrt{n})$, only the terms in the first line are of order O(1/n), and the rest can be neglected.

**Example 1: Transformation of a normal random variable**

Consider a random variable *X* from a normal distribution with density $g_{\tau }\left(x\right)=\varphi \left(x;x_{0},\sigma^{2}/n+\tau^{2}\right)$, where $\varphi (\mu ,\sigma^{2})$ is the normal density with mean μ and variance σ^2^. Here $x_{\tau }=x_{0}$, so *X* is unbiased for *x*_0_ even under the REM, and the variance is $\sigma^{2}/n+\tau^{2}$. If we transform to f(X), our formula for the bias gives

$$\frac{\sigma^{2}/n+\tau^{2}}{2}\frac{d^{2}f\left(x\right)}{dx^{2}}{|}_{x_{0}}\;.$$

In this case the overdispersion is of size τ^2^ and in a nonlinear transformation it causes an added bias of size $0.5\tau^{2}f^{\prime \prime }{\left(x\right)|}_{x_{0}}$. This does not tend to 0 with n→∞.

**Example 2: Standard random effects model for log‐odds**

In a meta‐analysis, the following standard additive REM for the empirical log‐odds (θ=log(p/(1−p))) is used routinely: $\hat{\theta }=X∼N\left(\theta ,{\left[np\left(1-p\right)\right]}^{-1}+\tau^{2}\right)$, where *p* is the probability of an event of interest. We are interested in going from the logit scale θ to the probability scale *p*. This transformation is

$$p=f\left(\theta \right)={\left[1+\exp\left(-\theta \right)\right]}^{-1}\;.$$

It follows that $\hat{p}=f\left(X\right)$ has a bias of size

$$\frac{1}{2}f^{\prime \prime }\left(\theta \right)\left\{{\left[np\left(1-p\right)\right]}^{-1}+\tau^{2}\right\}=\frac{1}{2}p\left(1-p\right)\left(1-2p\right)\left[\frac{1}{np(1-p)}+\tau^{2}\right]\;.$$

This follows from $f^{\prime }\left(\theta \right)=f\left(\theta \right)-f{\left(\theta \right)}^{2}$ and

$$f^{\prime \prime }\left(\theta \right)=\frac{\exp(-\theta )(\exp(-\theta )-1)}{{\left(1+\exp\left(-\theta \right)\right)}^{3}}=p\left(1-p\right)\left(1-2p\right)\;.$$

The bias is nonzero unless p=1/2. For p=0.1, the bias is $0.4/n+0.036\tau^{2}$; and for p=0.2, it is $0.3/n+0.048\tau^{2}$. Note again the dramatic effect of the REM parameter τ.

**Example 3: Overdispersed binomial model for log‐odds**

Consider the sample log‐odds in any overdispersed binomial model. We denote the overdispersion parameter in this model by ρ instead of τ, as this corresponds to the correlation between each pair of underlying Bernoulli variables $X_{1},...,X_{n}$. The transformation of interest is f(p)=log(p/(1−p)). The derivatives are $f^{\prime }\left(p\right)={\left[p\left(1-p\right)\right]}^{-1}$ and $f^{\prime \prime }\left(p\right)=\left(2p-1\right)/{\left[p\left(1-p\right)\right]}^{2}$. When the log‐odds transformation is applied to $X=\hat{p}=\sum_{i=1}^{n}X_{i}/n$, Eq. (4) is used with the first two moments of an overdispersed binomial distribution, $\mu_{\rho }\left(\hat{p}\right)=p$ and ${\text{Var}}_{\rho }\left(\hat{p}\right)=n^{-1}p\left(1-p\right)\left[1+\left(n-1\right)\rho \right]$, to obtain

$$\begin{array}{ccc} {\text{E}}_{\rho }\left(\log\left(\frac{\hat{p}}{1-\hat{p}}\right)\right) & = & \log\left(\frac{p}{1-p}\right)+\frac{\left(2p-1\right)}{2p^{2}{\left(1-p\right)}^{2}}{\text{Var}}_{\rho }\left(\hat{p}\right)+\cdots =\\ & = & \log\left(\frac{p}{1-p}\right)-\frac{\left(1-2p)(1+(n-1)\rho \right)}{2np(1-p)}+\cdots \end{array}.$$

Therefore, the sample log‐odds has an additional bias term linear in ρ in the overdispersed binomial model. For p=0.1, the total bias is −4.444[1+(n−1)ρ]/n; and for p=0.2, this bias is −1.875[1+(n−1)ρ]/n.

The standard bias correction for log‐odds due to Gart et al. (1985) is to add 1/2 to *X* and to n−X, that is to use $\tilde{p}=\left(X+1/2\right)/\left(n+1\right)$ when estimating the log‐odds. As is well known, this correction eliminates the 1/n bias term of the log‐odds at the null model ρ=0. When using this correction, the bias term $f^{\prime }\left(p\right)\text{E}\left(\tilde{p}-p\right)={\left[p\left(1-p\right)\right]}^{-1}\left(1-2p\right)/\left(2\left(n+1\right)\right)+O\left(n^{-2}\right)$ is added in Eq. (4), and the variance is multiplied by ${\left[n/\left(n+1\right)\right]}^{2}$ and we are left with

$$\begin{array}{ccc} {\text{E}}_{\rho }\left(\log\left(\frac{\tilde{p}}{1-\tilde{p}}\right)\right) & = & {\text{E}}_{\rho }\left(\log\left(\frac{X+1/2}{n-X+1/2}\right)\right)=\\ & = & \log\left(\frac{p}{1-p}\right)+\frac{\left(1-2p\right)}{2p(1-p)(n+1)}-\frac{\left(1-2p)n(1+(n-1)\rho \right)}{2p\left(1-p\right){\left(n+1\right)}^{2}}+\cdots \;. \end{array}$$

**Figure 1:**
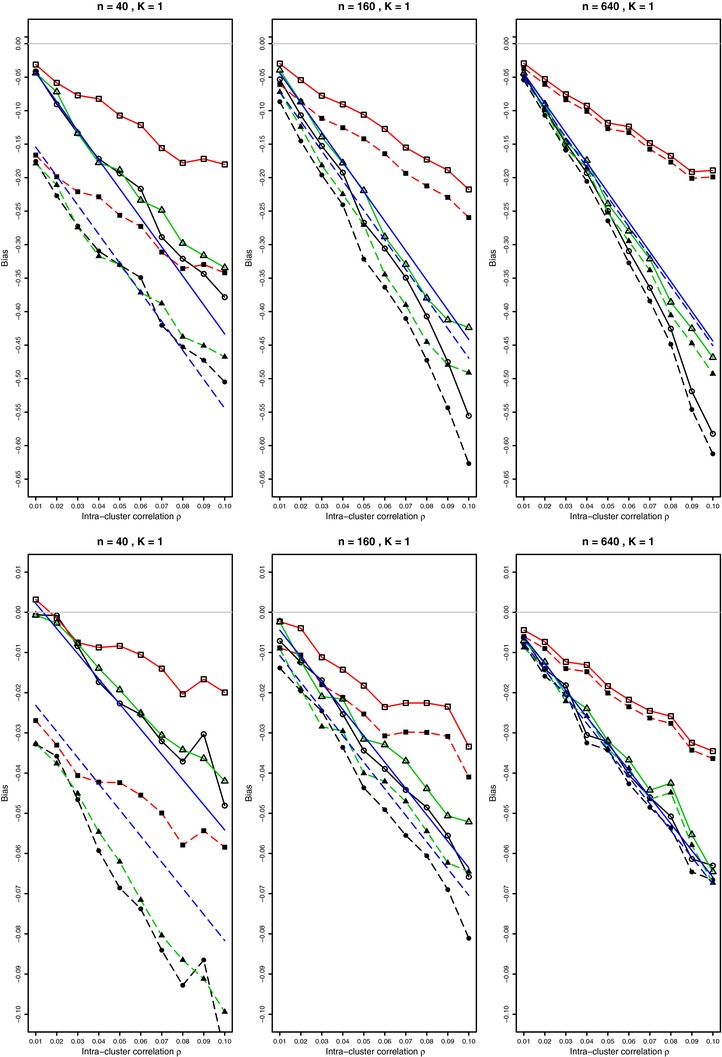
Biases of log‐odds (top) and arcsine (bottom) transformations on logit and arcsine scales, respectively, in overdispersed binomial model for p=0.1 and 0.01≤ρ≤0.1. For each value of ρ, 10,000 simulations from the beta‐binomial distribution (circles); from the Lunn and Davies model (squares); from the Gaussian Copula model (triangles), with and without the Gart (top) or Anscombe (bottom) corrections (solid and dashed lines, respectively); also the linear bias given by the first two terms of Eq. (4) using known values of *p* and ρ. Light gray line at zero.

To assess the precision of these two‐moment approximations to the bias with and without the Gart et al. (1985) correction, we performed 10,000 simulations for p=0.1 at each value of ρ from 0.01 to 0.1 in increments of 0.01, for various *n* values from 10 to 1000, generating overdispersed binomial variables from the beta‐binomial distribution, from the GC model of Emrich and Piedmonte (1991) and from the model of Lunn and Davies (1998). The results are given in Fig. 1 (first row). See also Supporting Information Fig. A2 in the Web Appendix. The values of *p* and ρ were known in these simulations. The approximation works well for small values of ρ in the beta‐binomial and GC models, but does badly for the Lunn–Davies model. Thus knowing just two moments of a distribution does not provide sufficient information on the magnitude of bias.

### 2.2 Variance‐stabilizing transformations in overdispersed families

Variance‐stabilizing transformations (v.s.t.'s) are used when the variance (under the fixed effect model) is a function of the mean: ${\text{Var}}_{0}\left(X\right)=h\left({\text{E}}_{0}\left(X\right)\right)$. The aim of a v.s.t. is to achieve ${\text{Var}}_{0}\left(f\left(X\right)\right)\approx 1$. To be a v.s.t. when τ=0, a transformation f(X) must satisfy

$$[f^{\prime }{\left({\text{E}}_{0}\left(X\right)\right]}^{2}={\left[{\text{Var}}_{0}\left(X\right)\right]}^{-1}=1/h\left({\text{E}}_{0}\left(X\right)\right)\;;$$

see Kulinskaya et al. (2008) for details and examples. Substituting this expression in equation (5), we obtain that, up to terms of smaller order,

(6) $${\text{Var}}_{\tau }\left[f\left(X\right)\right]=\frac{{\text{Var}}_{\tau }\left(X\right)}{{\text{Var}}_{0}\left(X\right)}\;.$$

It follows that for an additive REM, where under $g_{\tau }\left(x\right)$ the variance ${\text{Var}}_{\tau }\left(X\right)={\text{Var}}_{0}\left(X\right)+\tau^{2}$, the null v.s.t. f(x) does not stabilize the variance for τ≠0.

**Example 4: Overdispersed binomial model for arcsine transformation**

As an example, consider once more the arcsine transformation $f\left(p^{*}\right)=2\text{arcsin}\left(\sqrt{p^{*}}\right)$ for $p^{*}=\left(X+3/8\right)/\left(n+3/4\right)$, which is routinely used to variance‐stabilize binomial variables and is due to Anscombe (1948). Consider, first, the arcsine transformation without Anscombe correction, that is applied to $\hat{p}=X/n$. The derivatives are $f^{\prime }\left(p\right)={\left[p\left(1-p\right)\right]}^{-1/2}$ and $f^{\prime \prime }\left(p\right)=-\left(1/2\right){\left[p\left(1-p\right)\right]}^{-3/2}\left(1-2p\right)$. The bias under overdispersion ρ>0 is

(7) $${\text{E}}_{\rho }\left[2\text{arcsin}(\sqrt{\hat{p}})\right]-2\text{arcsin}\left(\sqrt{p}\right)=-\frac{1}{4}\frac{\left(1-2p\right)}{\sqrt{p(1-p)}}\frac{\left[1+(n-1)\rho \right]}{n},$$

and the variance is $n^{-1}\left[1+\left(n-1\right)\rho \right]$. With Anscombe correction, we need to add the first‐order bias term to the above formula. Using $p^{*}=\left(n\hat{p}+3/8\right)/\left(n+3/4\right)$, the bias is

(8) $${\text{E}}_{\rho }\left[2\text{arcsin}(\sqrt{p^{*}})\right]-2\text{arcsin}\left(\sqrt{p}\right)=\frac{3(1-2p)}{2\sqrt{p(1-p)}\left(4n+3\right)}-\frac{\left(1-2p\right)}{\sqrt{p(1-p)}}\frac{4n[1+(n-1)\rho ]}{{\left(4n+3\right)}^{2}}.$$

For p=0.1, the bias is −(2/3)[1+(n−1)ρ]/n and the additional bias from the overdispersion is −(2/3)ρ; and for p=0.2, the bias is −0.375[1+(n−1)ρ]/n with an additional bias of −0.375ρ.

To assess the bias of the arcsine transform and the precision of our two‐moment approximation to the bias for p=0.1, we performed 10,000 simulations for p=0.1 at each value of ρ from 0.01 to 0.1 in increments of 0.01, for various values for *n* from 10 to 1000, generating overdispersed binomial variables from the beta‐binomial distribution, from the model of Lunn and Davies (1998) and from the GC model of Emrich and Piedmonte (1991). The results are given in Fig. 1 (second row). See also Supporting Information Fig. A3 in the Web Appendix. The linear bias term was plotted for the known values of *p* and ρ. Overall, the bias of the arcsine transformation is rather small. The approximation has the correct slope but not the intercept of a linear trend for smaller values of *n*. For larger *n*, the approximation is very good for the beta‐binomial and for the GC, but not for the Lunn–Davies model unless ρ≤.01. For larger ρ, the bias of the arcsine transform with the Lunn–Davies model is clearly not linear. The Anscombe (1948) correction reduces bias for all values of *n*, though it does not matter much for larger *n*. In this case the Lunn–Davies model results in a somewhat smaller bias than the beta‐binomial and the GC models.

We also studied the coverage of confidence intervals for *p* based on the normal approximation with the variance [1+(n−1)ρ] for known ρ to the arcsine transformation of $\hat{p}$ for the three models. The results are given in Supporting Information Fig. A4 in the Web Appendix.

Overall the coverage in the beta‐binomial and the GC models with Anscombe (1948) correction is pretty good. It becomes increasingly conservative with increasing ρ. The coverage deteriorates for larger sample sizes in the Lunn–Davies model. This is due to its asymptotic nonnormality, as discussed in Web Appendix, Section A.4.

## 3 Transformation bias when combining datasets

### 3.1 Small biases in meta‐analysis

In meta‐analysis or when combining group‐level data, a relation between the average sample size *n* and the number of studies/clusters *K* is important for the quality of the inference for the combined effect. We retain the meta‐analysis terminology in this Section, but it is just as applicable to the analysis of group‐level studies. In our context, if a statistic *X* estimating some parameter μ has a bias of order 1/n, the mean (weighted or not) of *K* such statistics has a bias of the same order, but its variance is of order 1/(nK). So keeping *n* fixed and increasing *K* results in diminishing coverage of μ as the narrower confidence intervals are centered on a biased estimator. This observation was originally made in Kulinskaya et al. (2014). In the current setting, a minor bias from a transformation used under REM may result in substandard coverage of the combined effect, as is demonstrated for the arcsine transform in Section 3.2. Therefore in meta‐analysis we cannot afford even small biases when *K* is large.

Denote by $n_{i},i=1,...,K$ the sample sizes and by $\bar{n}$ the “average” sample size of the *K* studies, and let the total sample size be $N=\bar{n}K$. Denote by $X_{i}$ a summary statistic from study *i*, and denote its expectation and variance by μ and $\sigma_{i}^{2}$. Note that $\sigma_{i}^{2}$ is of order $1/n_{i}$. Let the bias of the combined weighted mean $\bar{X}$ be $c/\bar{n}$, so the combined mean is centered at $\mu +c/\bar{n}$. If inverse‐variance weights $w_{i}$ are used in the combined mean, its variance is approximated by ${\left[\sum w_{i}\right]}^{-1}={\left[K\bar{w}\right]}^{-1}=O\left(1/N\right)$, where $\bar{w}$ is the average weight. Denote by $\sigma^{2}={\bar{w}}^{-1}=O\left(1/\bar{n}\right)$ the “average” variance of the within‐studies summary statistics $X_{i}$. The half‐width of the confidence interval (CI) for the combined mean of *K* studies is $z_{1-\alpha /2}\sigma /\sqrt{K}$. For the CI for the combined effect to reliably cover the mean μ, the requirement is

$$z_{1-\alpha /2}\sigma /\sqrt{K}>>\left|c\right|/\bar{n}.$$

This may not be satisfied when the number of studies *K* is too large, or the sample size $\bar{n}$ is too small. To achieve good coverage of the combined effect, given biased estimates from the individual trials whose bias is of rough order $1/\bar{n}=K/N$ , the following relation between the number of studies *K* and the overall sample size *N* should hold:

$$K=O(N^{1/2-\gamma })\;\text{for}\;\text{some}\;\gamma >0.$$

This means that the sample sizes of the individual studies in meta‐analysis cannot be too small in relation to the number of studies. In practice, as the constant *c* is not known, particular caution is required in the case of a large number of comparatively small studies. A similar complication arises, for instance, when combining penalised GLM regressions (which are intentionally somewhat biased) on subsamples of a big dataset. The even stronger restriction $K<N^{1/5}$ is required in that case in order for the combined result to be equivalent to the regression on the full dataset (Chen and Xie, 2012).

### 3.2 Arcsine transformation in meta‐analysis

We have studied by simulation the bias and coverage of the parameter $2\text{arcsin}(\sqrt{p})$ when the data is generated from an overdispersed binomial distribution with intracluster correlation coefficient ρ≤0.1. The estimated probabilities ${\hat{p}}_{i}$, i=1,...,K from the individual studies are arcsine‐transformed, and the meta‐analysis is performed on the variance‐stabilized scale. We varied the sample sizes from n=10 to n=1000 and the number of studies from K=10 to K=80. Inverse‐variance weights on the variance‐stabilized scale, $w_{i}=n_{i}/\left[1+\left(n_{i}-1\right)\rho \right]$ with known ρ, were used in the meta‐analysis

**Figure 2:**
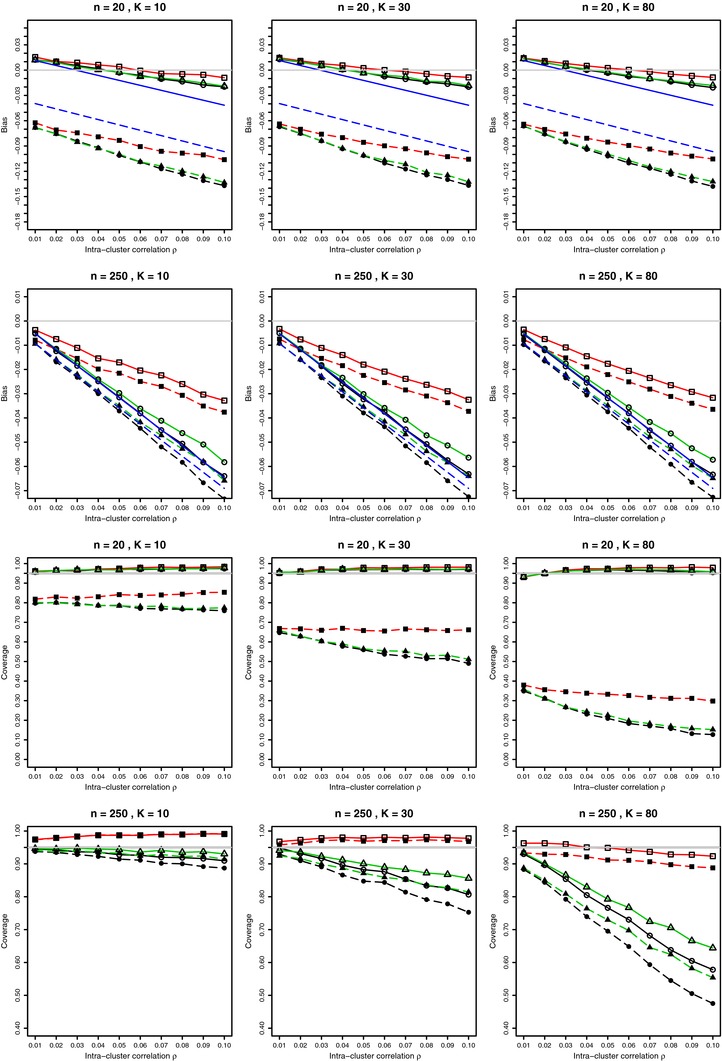
Bias on the arcsine scale and coverage at nominal 95% level of the combined mean in the meta‐analysis of arcsine transformations from *K* studies in overdispersed binomial model for p=0.1 and 0.01≤ρ≤0.1. For each value of ρ, 10, 000 simulations from the beta‐binomial distribution (circles); from the Lunn and Davies model (squares); from the Gaussian Copula model (triangles), with and without Anscombe correction (solid and dashed lines, respectively). Also linear bias given by the first two terms of Eqs. (7) and (8) and plotted for known *p* and ρ. Light gray lines at zero for bias and at 0.95 for coverage.

A representative selection of these simulation results when p=0.1 is given in Fig. 2 for the bias and the coverage of the combined mean (with the known ρ in weights) of the arcsine‐transformed estimated probabilities from *K* studies. Detailed results for bias and coverage for p=0.1,0.2, and 0.4 are given in Supporting Information Figs. A5 and A10 in the Web Appendix. The coverage of the combined mean when the parameter ρ is estimated is explored in Section 3.3.

The Anscombe correction substantially reduces bias and improves coverage. For each sample size, coverage deteriorates as the number of studies *K* increases. The reason is that the bias, though small, becomes nonnegligible for large *K*, as discussed in Section 3.1. Interestingly, for large *n* (starting from n=80), there is a substantial difference in coverage between the beta‐binomial and GC models, and the Lunn–Davies model. Coverage in the Lunn–Davies model is close to nominal, whereas in the other two models, coverage deteriorates quite dramatically. For p=0.2 and p=0.4 the above overall patterns also apply but on a milder scale, especially for p=0.4, see Supporting Information Figs. A7 and A10 in the Web Appendix.

### 3.3 Bias correction for arcsine transformation in meta‐analysis

In this section, we aim to correct the bias in the arcsine transformation by taking out the first‐order bias terms given by Eqs. (7) and (8). The bias terms depend on $\left(1-2p\right)/\sqrt{p(1-p)}$ and ρ. The bias correction using known values of *p* and ρ substantially improves both bias and coverage, see Supporting Information Figs. A11 and A13 in the Web Appendix. Substituting an estimate $\hat{p}$ in the expressions for bias results in an additional bias in the expected value of $\left(1-2\hat{p}\right)/\sqrt{\hat{p}\left(1-\hat{p}\right)}$. This bias is minimised when the Gart et al. (1985) correction $\tilde{p}=\left(X+0.5\right)/\left(n+1\right)$ is used in the bias term.

For the intracluster correlation coefficient ρ various estimators were reviewed by Ridout et al. (1999). The analysis of variance (AOV) estimator and an estimator based on a weighted average of Pearson correlation coefficients between pairs of observations within each group, denoted ${\hat{\rho }}_{PPR}$ by Ridout et al. (1999) perform best in terms of bias. Our own simulations show that the AOV estimator, ${\hat{\rho }}_{AOV}$, defined in Web Appendix (Section B), is superior to the ${\hat{\rho }}_{PPR}$ estimator, see Supporting Information Fig. A20.

**Figure 3:**
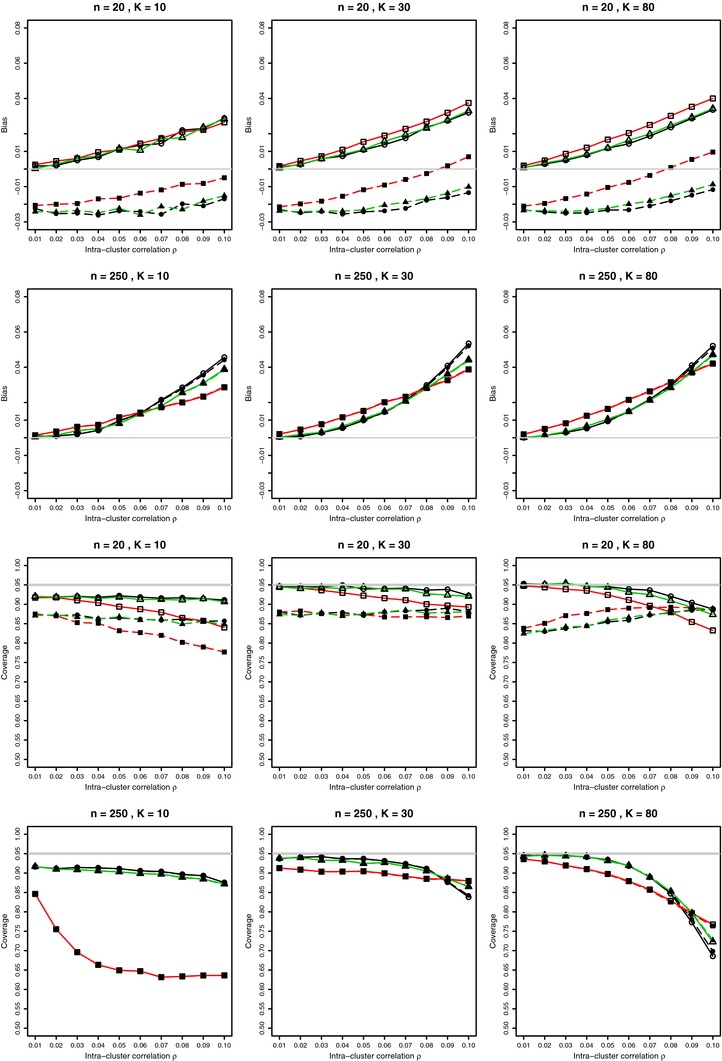
Bias on the arcsine scale and coverage at nominal 95% level of the combined mean of bias‐corrected arcsine transformations from *K* studies in overdispersed binomial model for p=0.1 and 0.01≤ρ≤0.1 with estimated probabilities ${\hat{p}}_{i}$ and ${\hat{\rho }}_{AOV}$ in the bias correction terms. For each value of ρ, 10, 000 simulations from the beta‐binomial distribution (circles); from the Lunn and Davies model (squares); from the Gaussian Copula model (triangles), with and without Anscombe correction (solid and dashed lines, respectively). Light gray lines at zero for bias and at 0.95 for coverage.

We have studied by simulation the changes to bias and coverage of the $2\text{arcsin}(\sqrt{p})$ when the bias correction based on the estimated first‐order bias term is applied to the arcsine transformation and meta‐analysis is performed on the variance‐stabilized scale. A representative selection of these simulation results for the bias and the coverage when p=0.1 is given in Fig. 3. The intraclass correlation was estimated by ${\hat{\rho }}_{AOV}$, and the Gart et al. (1985) 1/2 correction was applied to $\tilde{p}$ in the bias term. We varied sample sizes *n* from 10 to 1000 and the number of studies *K* from 10 to 80. In meta‐analysis, inverse‐variance weights on the variance‐stabilized scale were used: $w_{i}=n_{i}/\left[1+\left(n_{i}-1\right){\hat{\rho }}_{AOV}\right]$.

Comparing the first two rows of Fig. 3 with respective rows of Fig. 2, we see that the bias correction reduced the bias, especially for small values of the intraclass correlation ρ≤0.06. For larger values of ρ, the bias correction results in a positive bias, compared with negative bias without the correction. The bias after correction increases for large values of ρ.

Comparing the lower two rows of Fig. 3 with respective rows of Fig. 2, it is clear that the bias correction improves coverage in the beta‐binomial and the GC models for ρ≤0.06 and K≥30. The coverage deteriorates for larger values of $\hat{\rho }$. For K=10 the bias correction results in coverage at about 90% at nominal 95% level. The reason is the inferior estimation of ρ for small values of *K*. For the Lunn–Davies model, the correction decreases coverage.

### 3.4 Meta‐analysis of log‐odds: Complications with unstable weights

In a meta‐analysis of the log‐odds parameter log(p/(1−p)) from an overdispersed binomial distribution with intracluster correlation ρ, the weight of an estimated log‐odds is given by the inverse estimated variance $w=\left(1+\left(n-1\right)\hat{\rho }\right)/\left(n\hat{p}\left(1-\hat{p}\right)\right)$. In contrast to the arcsine‐transformed parameter, the weights of log‐odds estimates depend on the unknown probabilities. Estimation of the probabilities affects the bias of the log‐odds, and even its sign.

**Figure 4:**
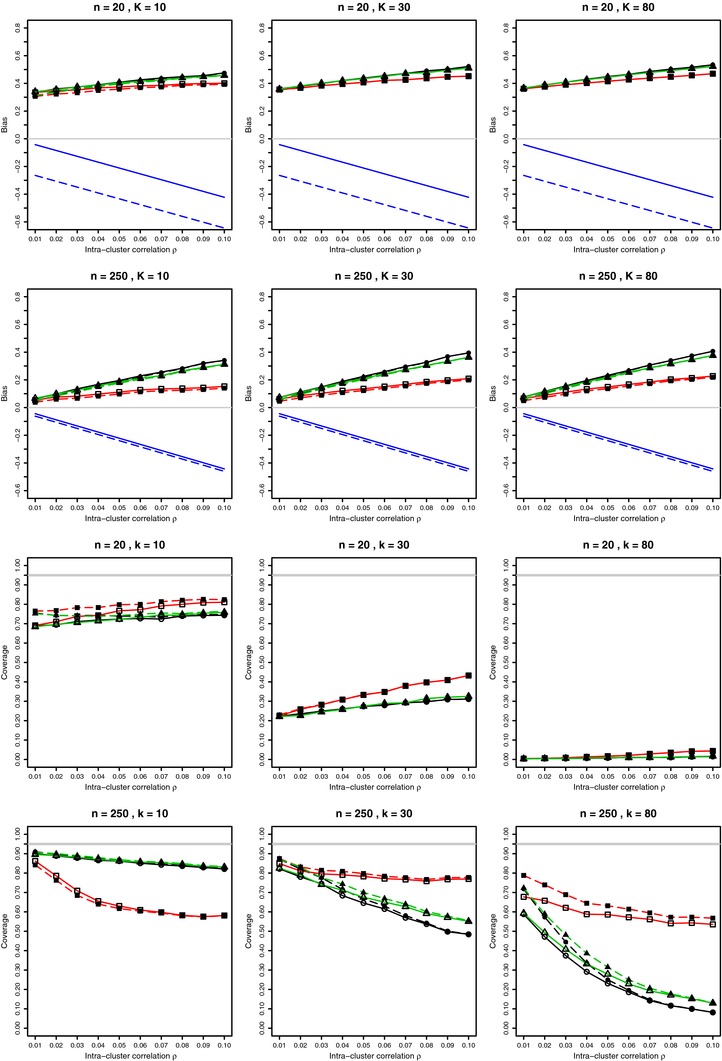
Bias on log‐odds scale and coverage at nominal 95% level of the combined mean of bias‐corrected log‐odds from *K* studies in overdispersed binomial model for p=0.1 (log(p/(1−p))=−2.20) and 0.01≤ρ≤0.1 with estimated probabilities ${\hat{p}}_{i}$ and ${\hat{\rho }}_{AOV}$ in the weights. For each value of ρ, 10, 000 simulations from the beta‐binomial distribution (circles); from the Lunn and Davies model (squares); from the Gaussian Copula model (triangles), with and without Gart correction (solid and dashed lines, respectively). Also the first‐order linear bias given by the first two terms of Eq. (4) with known values of *p* and ρ. Light gray lines at zero for bias and at 0.95 for coverage.

Trikalinos et al. (2013) studied by simulation the log, logit, and arcsine transformations for overdispersed binomial data. They rightly point out that “All these functions are concave for proportions between 0, 0.50, and therefore introduce a negative bias: The mean in the transformed scale will be smaller than the transformation of the mean in the proportion scale”. However, this theoretical finding is reversed when the probabilities *p* are estimated. Supporting Information Fig. A16 and Fig. A17 in Web Appendix show the bias and coverage of log‐odds in the meta‐analysis of *K* studies using known probabilities *p* and intracluster correlation ρ in the weights. The bias is very similar to the bias for a single study given in Fig. 1. The first‐order bias given by the first two terms of Eq. (4) approximates the bias of the log‐odds transformation reasonably well. Compare these results with those in Fig. 4, and with Supporting Information Fig. A18 and Fig. A19 in the Web Appendix showing bias and coverage when the weights include estimated probabilities and an estimated or known value of ρ. Coverage in the meta‐analysis of the log‐odds parameter is pretty dismal in all settings. However, the sign of the bias changes from negative to positive with substitution of $\hat{p}$ in the weights. New terms taking the random weights into account are required to estimate the bias. It is therefore considerably more difficult to provide bias correction for meta‐analysis of log‐odds, and we do not pursue this further.

## 4 Examples

An important application of our methodology is to group‐level studies or meta‐analyses of prevalence of a disease or a condition. In this section, we consider the severity of transformation bias and the usefulness of our correction to this bias in two examples of meta‐analyses of prevalence. The first example is that of syndromal depression in chronic kidney disease (Palmer et al., 2013), and the second is the prevalence of HIV infection in homeless people (Beijer et al., 2012). For both examples we obtained the results using the standard meta‐analytic methods for the arcsine‐transformed prevalences, and also our bias‐correcting methods. These varying techniques result in somewhat different estimates of prevalence. Our R program for meta‐analysis of prevalence is provided as Supplementary Information. To evaluate which method is more likely to provide a correct inference, we have performed three simulation studies for each example, using the three methods for generation of overdispersed binomial outcomes, the GC, the BB, and the LD methods. In all simulations we used the sample mean prevalence $\bar{p}$ and the estimated correlation ${\hat{\rho }}_{AOV}$ as the true values, and simulated 1000 new meta‐analytic datasets with the same number of studies and the same sample sizes as in the original meta‐analyses. For each simulation, we estimated the combined prevalence using the arcsine transformation with and without the Anscombe (1948) correction, and also with and without our bias correction.

### 4.1 Prevalence of syndromal depression for patients on dialysis

A meta‐analysis of 41 studies by Palmer et al. (2013) evaluated the prevalence of syndromal depression in chronic kidney disease (CKD). We consider the subset of 28 studies with N=2855 patients in total undergoing dialysis for CKD. According to Palmer et al. (2013), the dialysis stage has the highest rate of depressive symptoms. These data are provided in Supporting Information Table A1 in Web Appendix. Table A1 also includes estimated prevalences, their arcsine transformations and corresponding variances. The sample sizes in these 28 studies are unbalanced and the range of estimated prevalences is (0.0808,0.5484). The results of various meta‐analyses of these data are summarized in Table 1.

**Table 1:** Combined estimates of prevalence of syndromal depression and their confidence intervals for the data from Palmer et al. (2013)

Model	Anscombe correction	${\hat{p}}_{w}$	${\hat{p}}_{L}$	${\hat{p}}_{U}$
Fixed effects model (FE)	None	0.2060	0.1914	0.2211
	3/8	0.2081	0.1934	0.2232
Random effects model (REM)	None	0.2267	0.1904	0.2652
	3/8	0.2299	0.1937	0.2682
Overdispersed model (ODM)	None	0.2269	0.1900	0.2661
without bias correction	3/8	0.2302	0.1931	0.2696
Overdispersed model (ODM)	None	0.2357	0.1982	0.2753
with bias correction	3/8	0.2356	0.1982	0.2752

Cochran's *Q* statistic is Q=142.5, at K−1=27 degrees of freedom, indicating significant heterogeneity. In the standard random effects model for the arcsine‐transformed data, the DerSimonian‐Laird estimate of the between‐studies variance is ${\hat{\tau }}_{DL}^{2}=0.043$, and the combined estimate of prevalence is 0.227. The overdispersed model provides an estimated intracluster correlation of ${\hat{\rho }}_{AOV}=0.046$ and a very similar combined estimate of prevalence. The Anscombe (1948) correction increases this estimate to 0.230 for both models. The proposed bias correction increases it further to 0.236 when used both with and without the Anscombe correction, but the Anscombe correction does not seem to matter when the bias correction is used. The value of ρ=0.046 and the sample mean prevalence of $\bar{p}=0.2367$ were used in further simulations, summarised in Table 2.

**Table 2:** Quality of estimation of prevalence in meta‐analyses using the arcsine transformation and estimated or theoretical value of ρ in weights evaluated from 1000 simulated meta‐analyses of 28 studies with the value of ρ=0.046, and the prevalence of p=0.2367 with sample sizes from Palmer et al. (2013)

Generation method	Anscombe correction	Bias correction	Estimated ρ				Known ρ			
			$2\text{arcsin}(\sqrt{\hat{p}})$	Bias of vst	$\hat{p}$	Coverage	$2\text{arcsin}(\sqrt{\hat{p}})$	Bias of vst	$\hat{p}$	Coverage
Beta‐binomial	None	NO	0.9967	−0.0194	0.2285	0.9020	0.9974	−0.0187	0.2288	0.9170
	3/8	NO	1.0051	−0.0110	0.2320	0.9200	1.0059	−0.0102	0.2323	0.9370
	None	YES	1.0157	−0.0004	0.2365	0.9400	1.0195	0.0034	0.2381	0.9630
	3/8	YES	1.0157	−0.0004	0.2365	0.9410	1.0196	0.0034	0.2381	0.9630
Lunn‐Davies	None	NO	0.9983	−0.0178	0.2291	0.8850	1.0013	−0.0148	0.2304	0.9540
	3/8	NO	1.0061	−0.0100	0.2324	0.9030	1.0092	−0.0069	0.2337	0.9590
	None	YES	1.0180	0.0019	0.2375	0.9250	1.0184	0.0023	0.2376	0.9790
	3/8	YES	1.0179	0.0018	0.2374	0.9260	1.0182	0.0021	0.2375	0.9790
Gaussian Copula	None	NO	0.9974	−0.0187	0.2287	0.9100	0.9980	−0.0181	0.2290	0.9170
	3/8	NO	1.0057	−0.0104	0.2322	0.9210	1.0063	−0.0098	0.2325	0.9400
	None	YES	1.0148	−0.0013	0.2361	0.9310	1.0158	−0.0003	0.2365	0.9690
	3/8	YES	1.0148	−0.0013	0.2361	0.9320	1.0158	−0.0003	0.2365	0.9710

Overall, the bias of the arcsine transformation is reduced by bias correction, and the coverage is noticeably improved. Known ρ results in somewhat higher, and the estimated ρ in somewhat lower than nominal coverage, but the differences are within 2 percentage points in both cases when the bias correction is used. Once more, the Anscombe correction does not seem to be needed when the bias correction is used.

### 4.2 Prevalence of HIV in homeless people

A meta‐analysis of the data on N=10,886 participants in 16 studies by Beijer et al. (2012) evaluated prevalence of HIV infection in homeless people. These data are provided in Supporting Information Table A2 in Web Appendix. The main feature of these data is low prevalences, varying from 0 to 0.13. The results of meta‐analyses by various techniques after the arcsine transformation are summarized in Table 3.

**Table 3:** Combined estimates of prevalence of HIV in homeless people and their confidence intervals for the data by Beijer et al. (2012)

	Anscombe (1948) correction	${\hat{p}}_{w}$	${\hat{p}}_{L}$	${\hat{p}}_{U}$
Fixed effects model (FEM)	None	0.0482	0.0442	0.0523
	3/8	0.0495	0.0455	0.0536
Random effects model (REM)	None	0.0429	0.0223	0.0697
	3/8	0.0445	0.0241	0.0708
Overdispersed model	None	0.0427	0.0252	0.0645
without bias correction (ODM)	3/8	0.0444	0.0265	0.0666
Overdispersed model	None	0.0587	0.0379	0.0836
with bias correction (ODM)	3/8	0.0590	0.0382	0.0841

Cochran's *Q* statistic is 536.4036 at K−1=15 degrees of freedom, indicating significant heterogeneity. The standard random effects model for the arcsine‐transformed data provides the DerSimonian–Laird estimate of between study variance ${\hat{\tau }}_{DL}=0.054$ and combined estimate of prevalence of 0.043. The overdispersed model provides the estimated intracluster correlation of ${\hat{\rho }}_{AOV}=0.037$ and the same combined estimate of prevalence. The Anscombe (1948) correction increase these estimates to 0.045 and 0.044, respectively, for the two models. The proposed bias correction increases estimated prevalence to 0.059 when used both with and without the Anscombe correction.

The value of ρ=0.037, and the sample mean prevalence of $\hat{p}=0.054$ were used in further simulations, summarized in Table 4.

**Table 4:** Quality of estimation of prevalence in meta‐analyses using the arcsine transformation and estimated or theoretical value of ρ in weights evaluated from 1000 simulated meta‐analyses of 16 studies with the value of ρ=0.037, and the prevalence of p=0.054 with sample sizes from Beijer et al. (2012)

Generation method	Anscombe Correction	Bias Correction	Estimated ρ				Known ρ			
			$2\text{arcsin}(\sqrt{\hat{p}})$	Bias of vst	$\hat{p}$	Coverage	$2\text{arcsin}(\sqrt{\hat{p}})$	Bias of vst	$\hat{p}$	Coverage
Beta‐binomial	None	NO	0.4303	−0.0404	0.0456	0.8000	0.4264	−0.0443	0.0448	0.8600
	3/8	NO	0.4381	−0.0326	0.0472	0.8300	0.4346	−0.0361	0.0465	0.8990
	None	YES	0.4823	0.0116	0.0570	0.9070	0.4855	0.0148	0.0578	0.9630
	3/8	YES	0.4836	0.0129	0.0573	0.9110	0.4867	0.0160	0.0581	0.9630
Lunn‐Davies	None	NO	0.4491	−0.0216	0.0496	0.5790	0.4533	−0.0174	0.0505	0.9890
	3/8	NO	0.4532	−0.0175	0.0505	0.5830	0.4587	−0.0120	0.0517	0.9860
	None	YES	0.4790	0.0083	0.0563	0.5170	0.4945	0.0238	0.0599	0.9680
	3/8	YES	0.4790	0.0083	0.0563	0.5170	0.4945	0.0238	0.0599	0.9670
Gaussian Copula	None	NO	0.4317	−0.0390	0.0459	0.8050	0.4380	−0.0327	0.0472	0.9140
	3/8	NO	0.4387	−0.0320	0.0473	0.8450	0.4454	−0.0253	0.0488	0.9410
	None	YES	0.4820	0.0113	0.0570	0.9050	0.4859	0.0152	0.0579	0.9640
	3/8	YES	0.4829	0.0122	0.0572	0.9070	0.4868	0.0161	0.0581	0.9630

Overall, the negative bias of the arcsine transformation is reduced and becomes positive due to bias correction, and the coverage is noticeably improved. Known ρ results in somewhat higher, and the estimated ρ in considerably lower than nominal coverage, reaching 91% at 95% nominal level for the BB and the GC generated data as compared with 96–97% for all generation mechanisms when ρ is known and the bias correction is used. Unfortunately, for the LD generation the estimation of ρ by $\rho_{AOV}$ clearly does not work, resulting in abysmal coverage with or without the bias correction. Once more, the Anscombe (1948) correction does not seem to be of much benefit when the bias correction is used. To summarize, low prevalence is considerably more challenging to estimate correctly. The perils of routine use of transformations are very clear in this example, and the proposed bias correction is of much benefit.

## 5 Discussion

We have investigated bias arising in the estimation of transformed probabilities under the assumptions of random or mixed effects models, and its deleterious effects on inference in a meta‐analysis. We demonstrated and quantified these effects in the examples of arcsine and log‐odds transformations for overdispersed binomial data. In the standard additive REM of meta‐analysis, the random effect is modeled as the between‐study variance component τ^2^. In the overdispersion model (Kulinskaya and Olkin, 2014), the overdispersion parameter can be interpreted as the intracluster correlation coefficient. Both models can be described in the common framework of overdispersion.

Cox (1983) compared ML estimates ${\hat{\mu }}^{+}$ and $\hat{\mu }$ for the overdispersed and original models, respectively, under contiguous alternatives $g_{\tau }\left(x\right)$. He found that ${\hat{\mu }}^{+}-\hat{\mu }$ is proportional to τ unless the parametrization is chosen to eliminate bias of order $n^{-1}$ in μ. The model specification is important in this context: “*if the log linear model specifies a Poisson distribution for* $Y_{j}$ with $\log\text{E}\left(Y_{j}\right)=x_{j}^{T}\beta$, *the overdispersed model should have* $E\left(Y_{j}\right)=\exp\left(x_{j}^{T}\beta \right)$, *with* $\text{Var}\left(Y_{j}\right)>\text{E}\left(Y_{j}\right)$. *An overdispersed model in which* $Y_{j}$ *is considered to have a Poisson distribution with* $\log\text{E}\left(Y_{j}\right)=x_{j}^{T}\beta +\xi_{j}$, *where* $\xi_{j}$ *in turn is a random variable of expectation zero*, *would, however, lead to the inconsistencies*...” (Cox, 1983, p. 273).

In the same vein, we have demonstrated in Section 2.1 that for close alternatives to the fixed effect model, any nonlinear transformation of an overdispersed random variable has a bias that is linear in τ. We have seen in simulations, for both the log‐odds and arcsine transformations, that the reduction in bias of a transformation under the fixed effect model reduces bias under the REM. We have used the Gart et al. (1985) and Anscombe (1948) corrections to this end. Unfortunately, this, in general, is not sufficient to correct bias under the REM. Additionally, such correction is more complicated in the regression setting.

Gart et al. (1985) discuss bias reduction for the logit. Let the empirical logit be $L_{a}\left(X\right)=\log\left(X+a\right)/\left(n-X+a\right)$. “*It is not possible to recommend a universal correction* *a* *for* $L_{a}\left(x\right)$ *in weighted linear regression; sometimes* a=1/2 *is best, at other instances* 1/4,0,1/2, *or intermediate values are appropriate. The estimation of its variance also presents problems of bias and correlation*.” (Gart et al., 1985, p. 187).

We have also seen that, depending on the way the REM is defined, the primary statistic may be unbiased, but any transformation of this statistic is biased unless the transformation is a linear function of the primary statistic. Thus, linear models are bias‐free, but Generalised Linear Mixed Models (GLMMs) are not. Other popular classes of transformations that are affected are the variance‐stabilizing and the normalizing transformations. This may have important implications in data analysis, where these kinds of transformations are routinely performed. In Section 3, we demonstrated how large an effect of these small biases may be in the context of meta‐analysis, and explained the reasons for these findings.

Model misspecification bias in meta‐analysis of rates and proportions is discussed in Trikalinos et al. (2013, p.81). The authors simulated the properties of log, log‐odds and arcsine transformations of the estimated probability *p* under beta‐binomial and binomial‐uniform (i.e., discrete uniform) distributions. They noted a very small bias of the arcsine transformation compared with the log‐odds and log transformations, and recommended inference based on the arcsine transformation without Anscombe (1948) correction in meta‐analysis. We agree with their recommendation on the preferential use of the arcsine transformation, but our results show that their recommendation on not using the Anscombe correction cannot be accepted without reservations. They also noticed that for both the log‐odds and arcsine transformations “coverage appears to become worse with increasing *K*, and more so for scenarios where heterogeneity is large”, but failed to explain this pattern.

Our simulations confirm that the biases of the log‐odds and arcsine transformations are linear in ρ for small values of the intracluster correlation coefficient. These biases do not depend on the sample sizes or the number of studies *K* in a meta‐analysis and result in abysmal coverage of the combined effect for large *K*. As a remedy, we proposed a plug‐in bias correction for the arcsine transformation in meta‐analysis. For a large number of studies K≥30, and for well‐behaved overdispersed binomial distributions such as the beta‐binomial or the GC model, this correction improves coverage and reduces the bias for ρ≤0.06. For ρ>0.06, the coverage still deteriorates. For the log‐odds transformation of proportions, it is more difficult to provide a similar bias correction due to the dependence between the probabilities of the outcome and the weights. Our R programs for simulations are provided as Supplementary Information.

Random effects models are often written without any details on how the overdispersion is generated. However we demonstrated that knowing just two moments of a distribution is not sufficient. When the meta‐analysis includes just a few studies, the mechanism of randomness is difficult to ascertain. In such cases, our examples show that it will be nearly impossible to get a realistic bias correction for large ICC. How to safeguard against misspecification of the REM and which method to use in a meta‐analysis are open questions. If the REM is specified on the original scale *X*, the transformed effect measure f(X) is biased. It appears to be safer to specify the REM on the transformed scale when the inference on this scale is preferable. These considerations may apply in the context of a meta‐analysis, where the REM is rather artificial to start with, and therefore there is some freedom on how to define it. Such freedom is not ordinarily present in the analysis of real data, where the correct model is paramount.

## Conflict of interest

*The authors declare no conflict of interest*.

## Supporting information

As a service to our authors and readers, this journal provides supporting information supplied by the authors. Such materials are peer reviewed and may be re‐organized for online delivery, but are not copy‐edited or typeset. Technical support issues arising from supporting information (other than missing files) should be addressed to the authors.

Supporting Information

Supporting Information

## References

[bimj1692-bib-0001] Anscombe, F. J. (1948). The transformation of Poisson, binomial and negative‐binomial data. Biometrika 35, 246–254.

[bimj1692-bib-0002] Beijer, U. , Wolf, A. and Fazel, S. (2012). Prevalence of tuberculosis, hepatitis c virus, and hiv in homeless people: a systematic review and meta‐analysis. The Lancet Infectious Diseases 12, 859–870. 22914343 10.1016/S1473-3099(12)70177-9 PMC3494003

[bimj1692-bib-0003] Chen, X. and Xie, M. (2012). A split‐and‐conquer approach for analysis of extraordinarily large data. Technical report, 2012‐01, Dept. Statistics, Rutgers Univ.

[bimj1692-bib-0004] Cox, D. (1983). Some remarks on overdispersion. Biometrika 70, 269–274.

[bimj1692-bib-0005] Eldridge, S. , Ashby, D. , Feder, G. , Rudnicka, A. and Ukoumunne, O. (2004). Lessons for cluster randomized trials in the twenty‐first century: a systematic review of trials in primary care. Clinical Trials 1, 80–90. 16281464 10.1191/1740774504cn006rr

[bimj1692-bib-0006] Emrich, L. J. and Piedmonte, M. R. (1991). A method for generating high‐dimensional multivariate binary variates. The American Statistician 45, 302–304.

[bimj1692-bib-0007] Gart, J. J. , Pettigrew, H. M. and Thomas, D. G. (1985). The effect of bias, variance estimation, skewness and kurtosis of the empirical logit on weighted least squares analyses. Biometrika 72, 179–190.

[bimj1692-bib-0008] Gulliford, M. , Adams, G. , Ukoumunne, O. , Latinovic, R. , Chinn, S. and Campbell, M. (2005). Intraclass correlation coefficient and outcome prevalence are associated in clustered binary data. Journal of Clinical Epidemiology 58, 246–251. 15718113 10.1016/j.jclinepi.2004.08.012

[bimj1692-bib-0009] Hedges, L. and Olkin, I. (1985). Statistical Methods for Meta‐analysis. Academic Press, Orlando, FL.

[bimj1692-bib-0010] Kulinskaya, E. , Morgenthaler, S. , and Staudte, R. G. (2008). Meta Analysis: A Guide to Calibrating and Combining Statistical Evidence. Wiley Series in Probability and Statistics. John Wiley & Sons, Chichester, UK.

[bimj1692-bib-0011] Kulinskaya, E. , Morgenthaler, S. and Staudte, R. G. (2014). Combining statistical evidence. International Statistical Review 82, 214–242.

[bimj1692-bib-0012] Kulinskaya, E. and Olkin, I. (2014). An overdispersion model in meta‐analysis. Statistical Modelling 14, 49–76.

[bimj1692-bib-0013] Littenberg, B. and MacLean, C. (2006). Intra‐cluster correlation coefficients in adults with diabetes in primary care practices: the vermont diabetes information system field survey. BMC Medical Research Methodology 6, 20. 16672056 10.1186/1471-2288-6-20 PMC1513389

[bimj1692-bib-0014] Lunn, A. D. and Davies, S. J. (1998). A note on generating correlated binary variables. Biometrika 85, 487–490.

[bimj1692-bib-0015] Palmer, S. , Vecchio, M. , Craig, J. C. , Tonelli, M. , Johnson, D. W. , Nicolucci, A. , Pellegrini, F. , Saglimbene, V. , Logroscino, G. , Fishbane, S. and Strippoli, G. F. (2013). Prevalence of depression in chronic kidney disease: systematic review and meta‐analysis of observational studies. Kidney International 84, 179–191. 23486521 10.1038/ki.2013.77

[bimj1692-bib-0016] Prentice, R. (1988). Correlated binary regression with covariates specific to each binary observation. Biometrics 44, 1033–1048. 3233244

[bimj1692-bib-0017] Ridout, M. S. , Demétrio, C. G. and Firth, D. (1999). Estimating intraclass correlation for binary data. Biometrics 55, 137–148. 11318148 10.1111/j.0006-341x.1999.00137.x

[bimj1692-bib-0018] Rücker, G. , Schwarzer, G. , Carpenter, J. and Olkin, I. (2009). Why add anything to nothing? The arcsine difference as a measure of treatment effect in meta‐analysis with zero cells. Statistics in Medicine 28, 721–738. 19072749 10.1002/sim.3511

[bimj1692-bib-0019] Trikalinos, T. A. , Trow, P. and Schmid, C. H. (2013). Simulation‐based comparison of methods for meta‐analysis of proportions and rates. Technical Report 13(14)‐EHC084‐EF, Agency for Healthcare Research and Quality (US). 24404633

