# Supplementary material for: Inference for binomial probability based on dependent Bernoulli random variables with applications to meta‐analysis and group level studies
Source: Biom J. 2016 May 18;58(4):896–914. doi: 10.1002/bimj.201500115 (PMC4999030; doi:10.1002/bimj.201500115)
Supplement: Supplementary file 1 — Supporting Information [file BIMJ-58-896-s001.pdf]

Web Appendix

A Generation of dependent Bernoullis

Algorithms for generating dependent Bernoulli random variables (r.v.'s) include normal or Archimedian copulas (Demirtas et al., 2009; Emrich and Piedmonte, 1991; Madsen and Birkes, 2013) and the method by Lunn and Davies (1998). In addition to summing dependent Bernoulli r.v., the beta-binomial distribution, methods based on sums of Poisson random variables (Demirtas et al., 2009), and Binomial mixtures (Qaqish et al., 2012) are available. Based on the first two moments, generalised estimating equations can be used for fitting the parameters (Rao Chaganty and Joe, 2004), but without knowledge of the probability frequency function, likelihood methods cannot be used. In any case, the third and forth moments are required to compare the asymptotic efficiency of various methods. The three methods used in the rest of this paper to generate the overdispersed binomial data are described in more detail below. These are the gaussian copula (GC) method by Emrich and Piedmonte (1991), the method by Lunn and Davies (1998) and the beta-binomial distribution.

A.1 Generation through Gaussian copula

A simple method for generation of correlated binary data is proposed by Emrich and Piedmonte (1991). The aim is to generate n Bernoulli r.v.'s $X_1, ..., X_n$ distributed as in (**??**). Let $Z = (Z_1, ..., Z_n)$ be a vector of independent standard normal r.v.'s. Denote by Σ the $n \times n$ covariance matrix such that

$$\Sigma = (1 - \rho^*)I_n + \rho^* J_n,$$

where ρ^* is the correlation, J_n is the matrix of 1's and I_n is the identity matrix of size $n \times n$. Let A be the lower triangular matrix resulting from a Cholesky decomposition $\Sigma = AA^T$, and the random variables $Y_1, ..., Y_n$ equal to $Y = AZ$. Finally, let $U = (\Phi(Y_1), ..., \Phi(Y_n))$. The binomial inverse transformation of $U_1, \ldots, U_n$ then produces correlated Binomial variables with correlations ρ. According to Emrich and Piedmonte (1991) and Demirtas et al. (2009), the link between ρ^* and ρ is given by $\Phi[z(p_i), z(p_j); \rho^*_{ij}] = \rho_{ij}(p_i q_i p_j q_j)^{1/2} + p_i p_j$, which for a given ρ can be solved using the bisection method. Here, $z(p)$ denotes the pth quantile of the standard normal distribution, and $\Phi[x_1, x_2, \rho^*]$ is the standard bivariate normal distribution function with correlation coefficient ρ^*. This solution is unique as long as the restriction (**??**) for the correlations ρ_{ij} holds. This method of generation is called Gaussian copula (GC) model throughout the paper.

A.2 Generation by the method of Lunn and Davies (1998)

Lunn and Davies (1998) consider the case of clustered binary variables

$$\{X_{ij},\ i = 1, \cdots, K; j\ =\ 1, \cdots, n_i\},$$

where i is the cluster and n_i is the size of each cluster. In order to generate correlated binary data with correlations ρ_i within each cluster i, firstly generate $n_i + 1$ independent Bernoulli random variables $\{Y_{ij}, j = 1, \cdots, n_i\}$ and Z_i from $B(1, p_i)$, where $B(n, p)$ is the binomial distribution with n trials and the probability of success p. Additionally, generate n_i independent Bernoulli variables U_{ij} from $B(1, \sqrt{\rho_i})$. The random variables $X_{ij} = (1 - U_{ij})Y_{ij} + U_{ij}Z_i$ for $j = 1, \cdots, n_i$ are then correlated binary r.v. such that $P(X_{ij} = 1) = p_i$, $\text{Var}(X_{ij}) = p_i(1 - p_i)$ and $\text{Cov}(X_{ij}, X_{is}) = \rho_i p_i(1 - p_i)$.

A.3 The Beta-Binomial distribution

The beta-binomial (BB) distribution is a mixture of Binomial distributions with fixed size n and a random success probability p following a Beta distribution. The Beta distribution is a popular choice for a mixture

distribution since it is the conjugate prior distribution for the parameter p if the data are binomial. When $Y_i \sim B(n_i, p_i)$ and $p_i \sim Beta(\alpha, \beta)$, then unconditionally, Y_i follows a beta-binomial distribution with parameters α, β and n_i. The expected value and variance of Y_i are

$$\mathrm{E}(Y_i) = \frac{n_i \alpha}{\alpha + \beta}, \qquad \mathrm{Var}(Y_i) = \frac{n_i \alpha \beta (n_i + \alpha + \beta)}{(\alpha + \beta)^2 (\alpha + \beta + 1)}.$$

It is more convenient to re-parametrize this distribution as $BB(n_i, \pi, \rho)$, where $\pi = \alpha/(\alpha + \beta)$ and $\rho = 1/(\alpha + \beta + 1)$. Then the moments are

$$\mathrm{E}(Y_i) = n_i \pi, \qquad \mathrm{Var}(Y_i) = n_i \pi (1 - \pi)(1 + (n_i - 1)\rho),$$

which shows the beta-binomial distribution to be an overdispersed binomial distribution. The density of the beta-binomial distribution is

$$P(Y_i = t; \pi, \rho) = \binom{n_i}{t} \prod_{j=0}^{t-1} (\pi + j\gamma) \prod_{j=0}^{n_i - t - 1} (1 - \pi + j\gamma) / \prod_{j=0}^{n_i - 1} (1 + j\gamma),$$

for $\gamma = \rho/(1 - \rho)$.

A.4 Large sample properties of the overdispersed binomial distributions

To better understand the properties of the overdispersed binomial distributions, we produced the QQ plots exploring large-sample normality or lack thereof, for the arcsine-transformed sample probabilities $\tilde{p} = (X + 3/8)/(n + 3/4)$ estimated from the data generated by the above three methods. The continuity correction $\tilde{p}$ was introduced by citeanscombe1948transformation) for use with the arcsine transformation. The arcsine transformation stabilizes the variance and is equal to 2arcsine($\sqrt{\tilde{p}}$). The QQ plots for several combinations of sample sizes and correlation coefficients ρ are given in Figure A1. It is clear that the Lunn-Davies model results in a much clumpier distribution. For small values of ρ, the beta-binomial and Gaussian copula models are close to normality, but the Lunn-Davies model is further from the normal, it is almost dichotomous for large n. For large n and large ρ all three distributions are almost dichotomous, but once more the Lunn-Davies much more so than the other two models. As we shall see, this results in much worse coverage of the confidence intervals based on the normal quantiles. In our further simulations, the Lunn-Davies model is the worst case scenario.

B Estimation of intra-class correlation ρ

The analysis of variance (AOV) estimator for intra-cluster correlation is

$$\hat{\rho}_{AOV} = \frac{MS_b - MS_w}{MS_b + (n_0 - 1) MS_w},$$

where MS_w and MS_b are the within and between group mean squares for a one-way analysis of variance applied to Bernoulli r.v.'s, and where

$$n_0 = \frac{1}{K - 1}[N - \sum_{j=1}^{K} \frac{n_j^2}{N}], \qquad \text{with} \qquad N = \sum_{j=1}^{K} n_j.$$

For binary outcomes, the within and between group mean squares are

$$MS_b = \frac{1}{K - 1}[\sum_{j=1}^{K} \frac{X_j^2}{n_j} - \frac{1}{N}(\sum_{j=1}^{K} X_j)^2]$$

© 0 WILEY-VCH Verlag GmbH & Co. KGaA, Weinheim

and

$$MS_w = \frac{1}{N-K}[\sum_{j=1}^{K} X_j - \sum_{j=1}^{K} \frac{X_j^2}{n_j}],$$

respectively.

Bias in the estimation of intra-class correlation ρ by ρ_{AOV} and ρ_{PPR} is plotted in Figure A20.

C Detailed results of simulations

Figure A2 shows bias of log-odds transformation for $p = 0.1$. Figures A3 and A4 provide bias and coverage of arcsine transformation for $p = 0.1$.

Detailed results for meta-analysis of arcsine transformed probabilities for $p = 0.1,\ 0.2$ and 0.4 (known p and ρ, no bias correction) are given in Figures A5– A10.

Coverage in meta-analysis of arcsine transformed probabilities with bias correction of arcsine transformation using known p and ρ is depicted in Figures A11, A12 and A13 for $p = 0.1$, $p = 0.2$ and $p = 0.4$, respectively. It can be seen that the coverage is greatly improved.

Figures A14 and A15 depict bias and coverage in meta-analysis of arcsine transformed probabilities with bias correction of arcsine transformation using estimated p and ρ.

Bias and coverage in meta-analysis of log-odds (known p and ρ) are depicted in Figures A16 and A17; Figures A18 andA19 provide bias and coverage for estimated p and known ρ. The sign of bias is reversed.

Bias in the estimation of intra-class correlation ρ by ρ_{AOV} and ρ_{PPR} is plotted in Figure A20.

D Datasets

Data for Examples 1 and 2 are provided in Tables A1 and A2.

© 0 WILEY-VCH Verlag GmbH & Co. KGaA, Weinheim

$n = 20, \;\; \rho = 0$

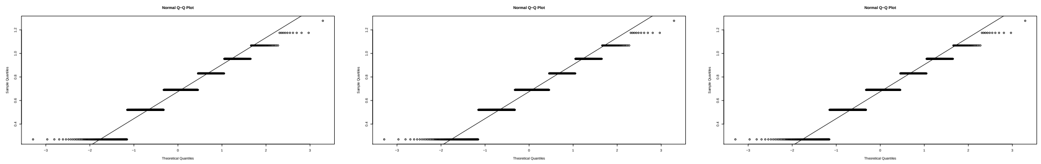

(a) Beta-Binomial (b) Gaussian Copula model (c) Lunn-Davies model

$n = 20, \;\; \rho = 0.1$

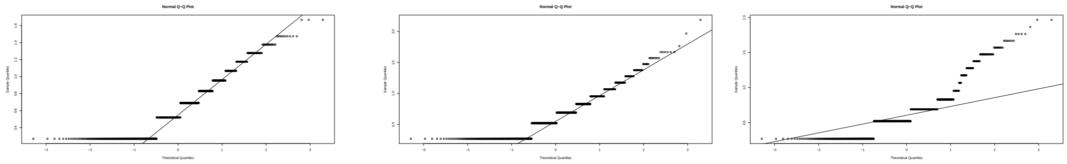

(d) Beta-Binomial (e) Gaussian Copula model (f) Lunn-Davies model

$n = 1000, \;\; \rho = 0.1$

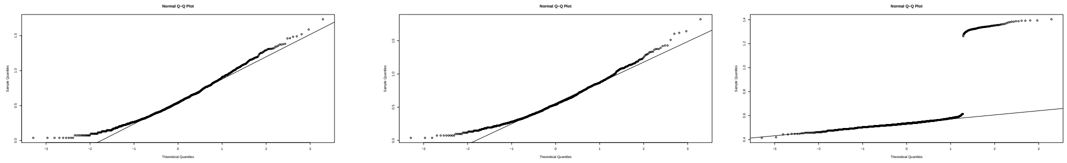

(g) Beta-Binomial (h) Gaussian Copula model (i) Lunn-Davies model

$n = 1000, \;\; \rho = 0.9$

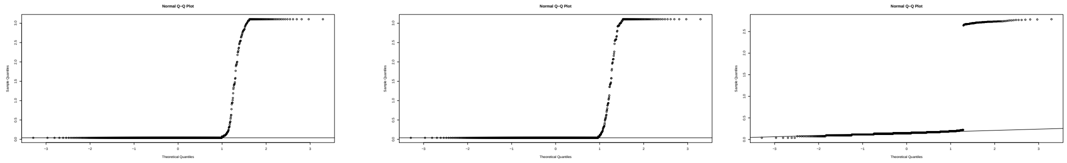

(j) Beta-Binomial (k) Gaussian Copula model (l) Lunn-Davies model

Figure A1 QQ plots for arcsine-transformed (with the continuity correction proposed in Anscombe (1948)) sample probabilities in Beta-Binomial, Gaussian Copula and Lunn-Davies models when the true probability is $p = 0.1$.

© 0 WILEY-VCH Verlag GmbH & Co. KGaA, Weinheim

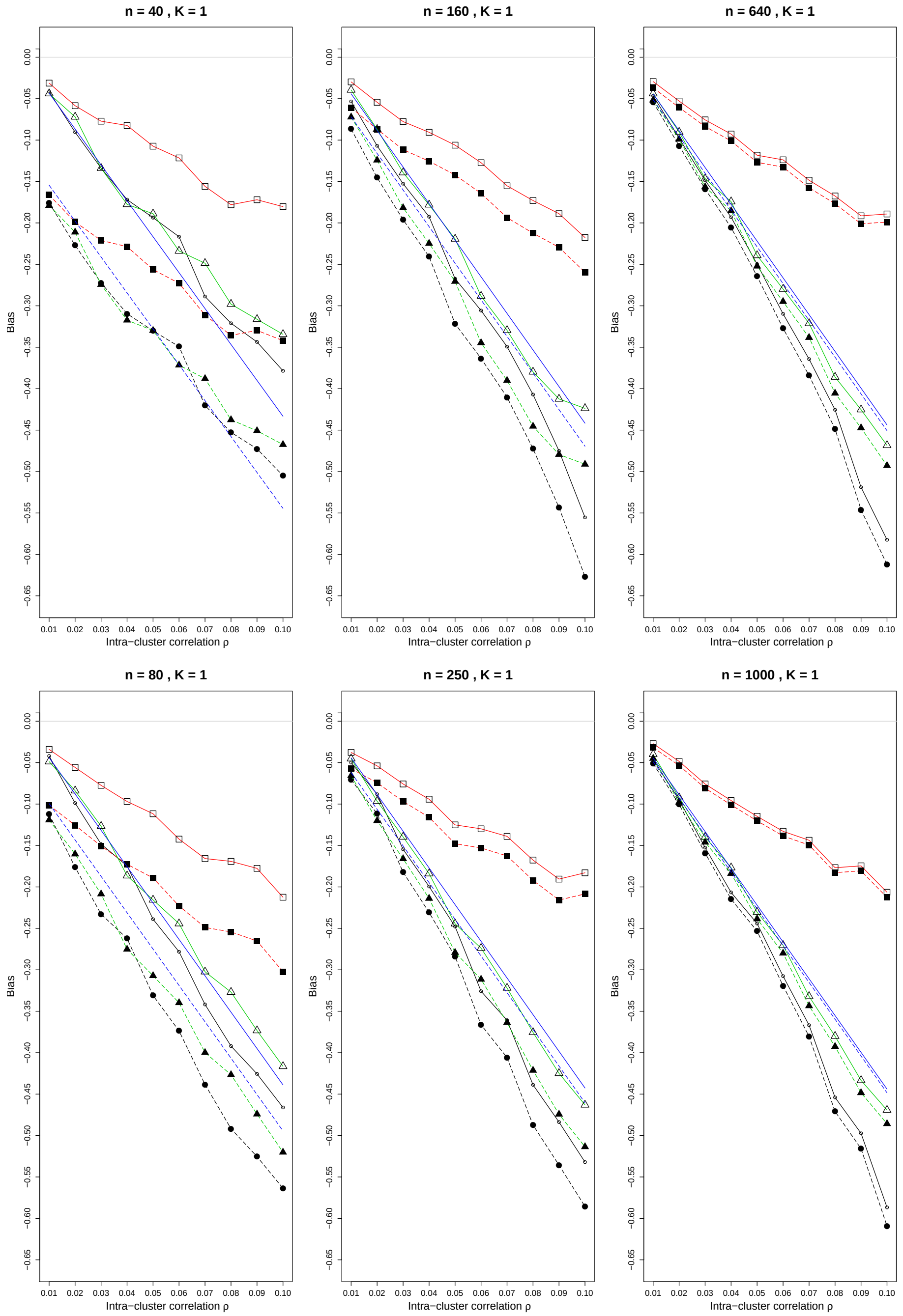

Figure A2 Bias on log-odds scale in overdispersed binomial model for $p = 0.1$ $(\log(p/(1-p)) = -2.20)$ and $0.01 \leq \rho \leq 0.1$. 10000 simulations for each value of ρ from the beta-binomial distribution (circles); from the Lunn and Davies (1998) model (squares); from the Gaussian copula Emrich and Piedmonte (1991) (triangles) and the first-order bias given by the first two terms of equation (equation (4)) with known values of p and ρ (blue), with and without the Gart et al. (1985) correction (solid and dashed lines, respectively). Light grey line at zero.

© 0 WILEY-VCH Verlag GmbH & Co. KGaA, Weinheim

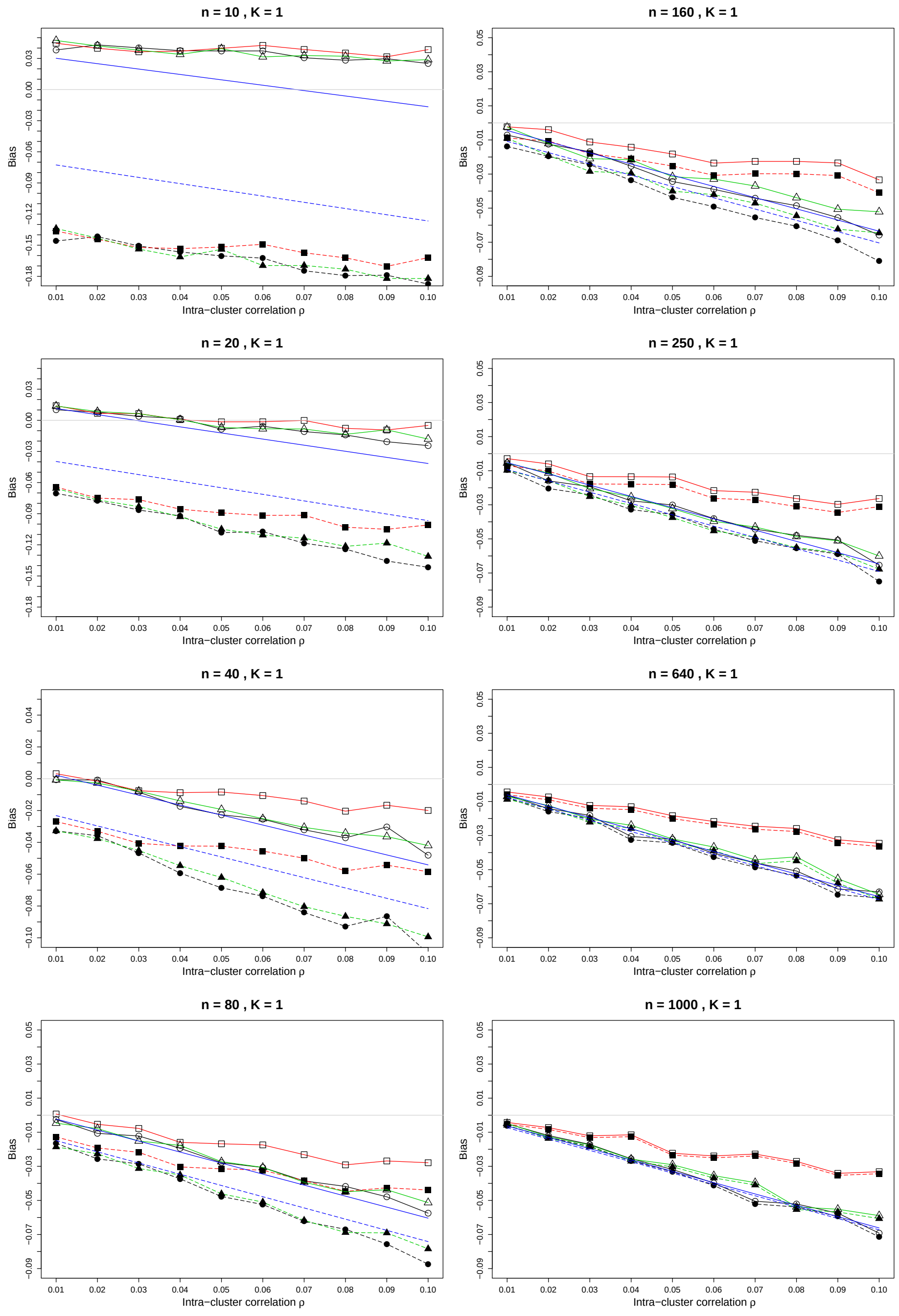

Figure A3 Bias on the arcsine scale of the arcsine transformation in overdispersed binomial model for $p = 0.1$ and $0.01 \leq \rho \leq 0.1$. 10000 simulations for each value of ρ from the beta-binomial distribution (circles), from the Lunn and Davies (1998) model (squares) and from the Gaussian copula Emrich and Piedmonte (1991) (triangles) with and without the Anscombe (1948) correction (solid and dashed lines, respectively). Also the first-order bias given by the first two terms of equations (equation (7)) and (equation (8)) and plotted for known p and ρ (solid or dashed blue lines). Light grey line at zero.

© 0 WILEY-VCH Verlag GmbH & Co. KGaA, Weinheim

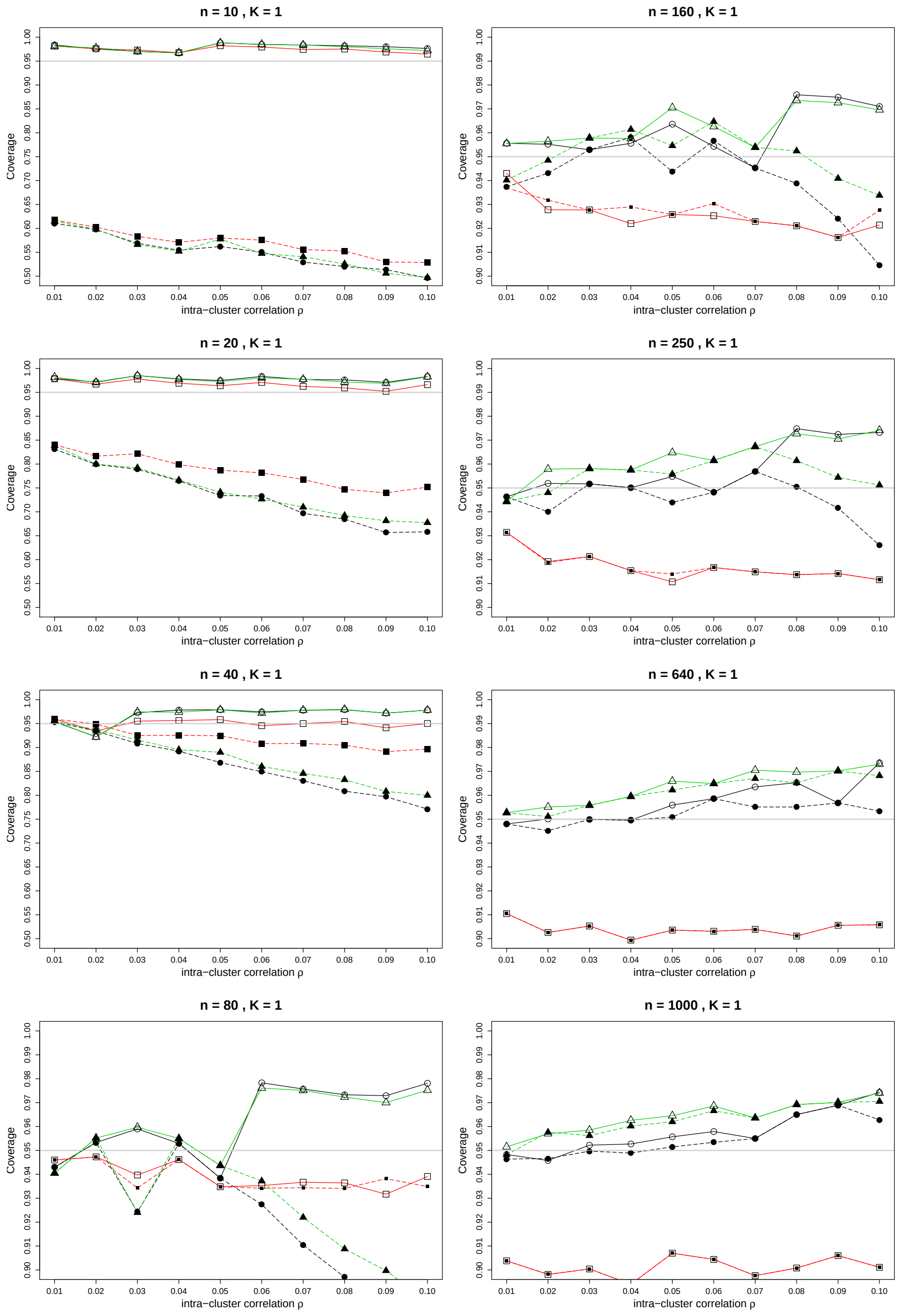

Figure A4 Coverage (for a known value of ρ) at the nominal 95% level of the true value of p using the arcsine transformation in overdispersed binomial model for $p = 0.1$ and $0.01 \leq \rho \leq 0.1$. 10000 simulations for each value of ρ from the beta-binomial distribution (circles), from the Lunn and Davies (1998) model (squares) and from the Gaussian copula of Emrich and Piedmonte (1991) (triangles) with and without the Anscombe (1948) correction (solid and dashed lines, respectively). Light grey line at 0.95.

© 0 WILEY-VCH Verlag GmbH & Co. KGaA, Weinheim

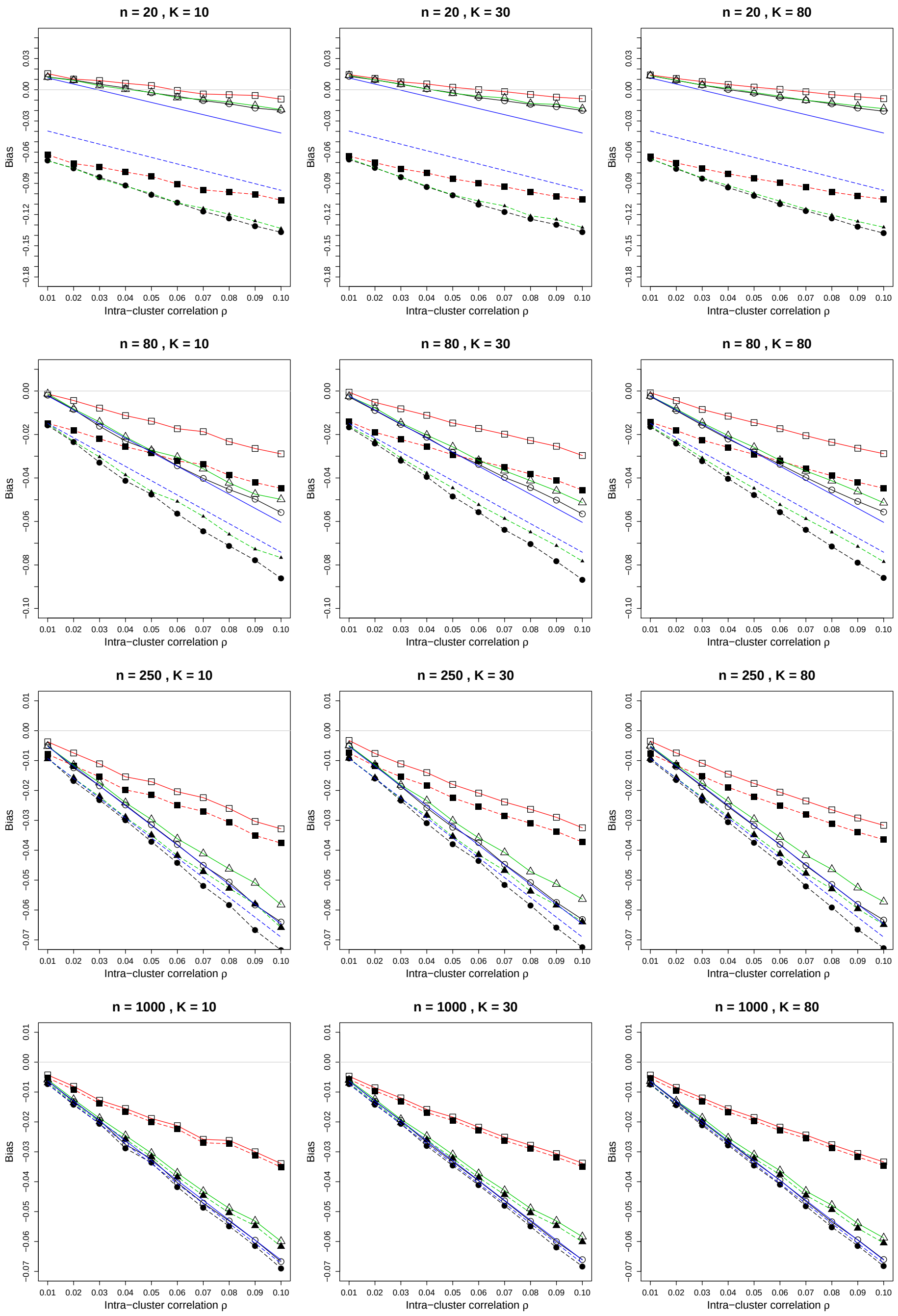

Figure A5 Bias on the arcsine scale of the meta-analysis of arcsine transformations from K studies in overdispersed binomial model for $p = 0.1$ and $0.01 \leq \rho \leq 0.1$. 10000 simulations for each value of ρ from the beta-binomial distribution (circles), from the Lunn and Davies (1998) model (squares) and from the GC model of Emrich and Piedmonte (1991) (triangles), with and without the Anscombe (1948) correction (solid and dashed lines, respectively). Also the first-order bias term given by the first two terms of equation (equation (4)) and plotted for known p and ρ (solid or dashed blue lines). Light grey line at zero.

© 0 WILEY-VCH Verlag GmbH & Co. KGaA, Weinheim

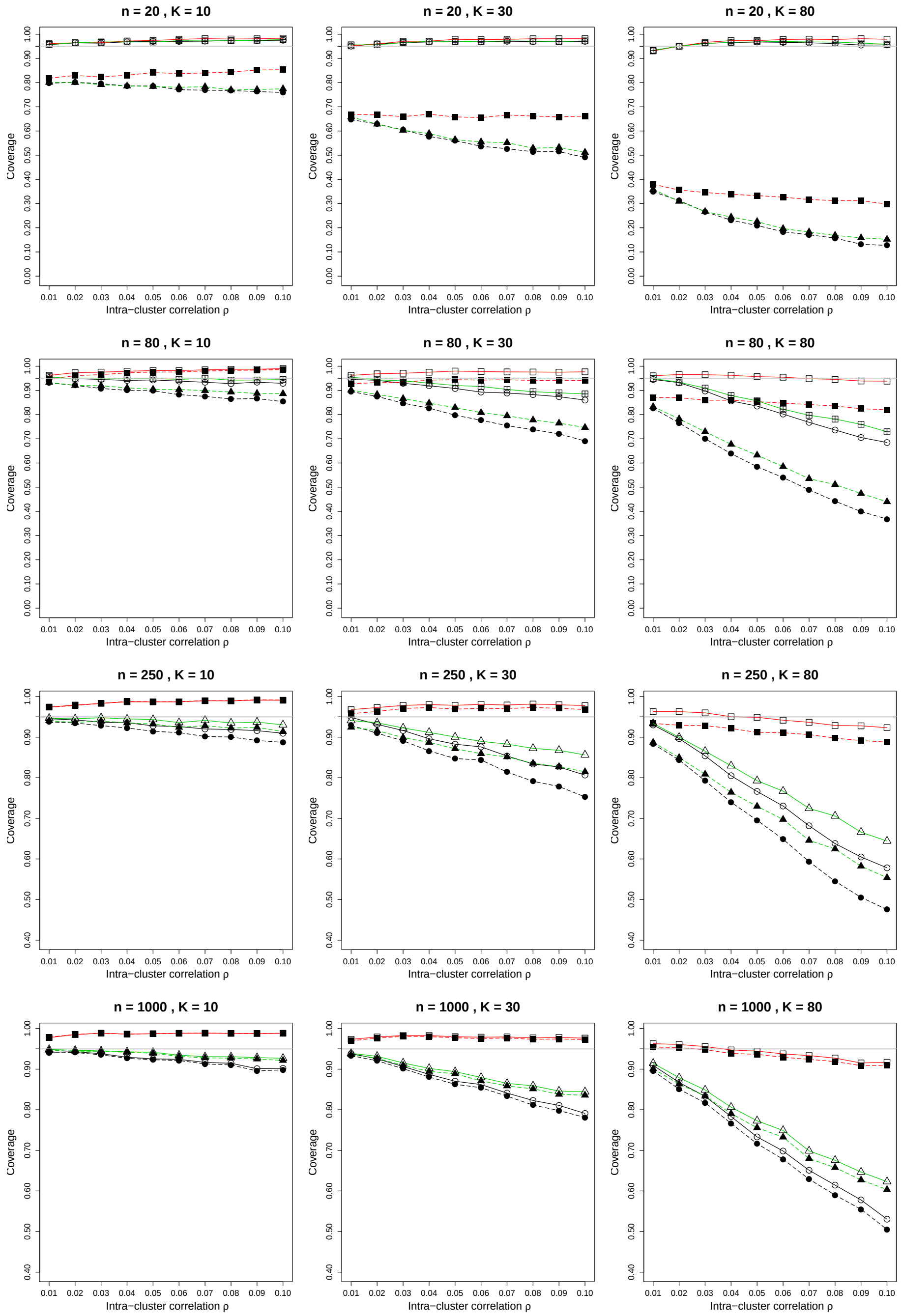

Figure A6 Coverage (for a known value of ρ) at the nominal 95% level of the true value of p using the meta-analysis of acrsine transformation from K studies in overdispersed binomial model for $p = 0.1$ and $0.01 \leq \rho \leq 0.1$. 10000 simulations for each value of ρ from the beta-binomial distribution (circles), from the Lunn and Davies (1998) model (squares) and from the GC model of Emrich and Piedmonte (1991) (triangles), with and without the Anscombe (1948) correction (solid and dashed lines, respectively). Light grey line at 0.95.

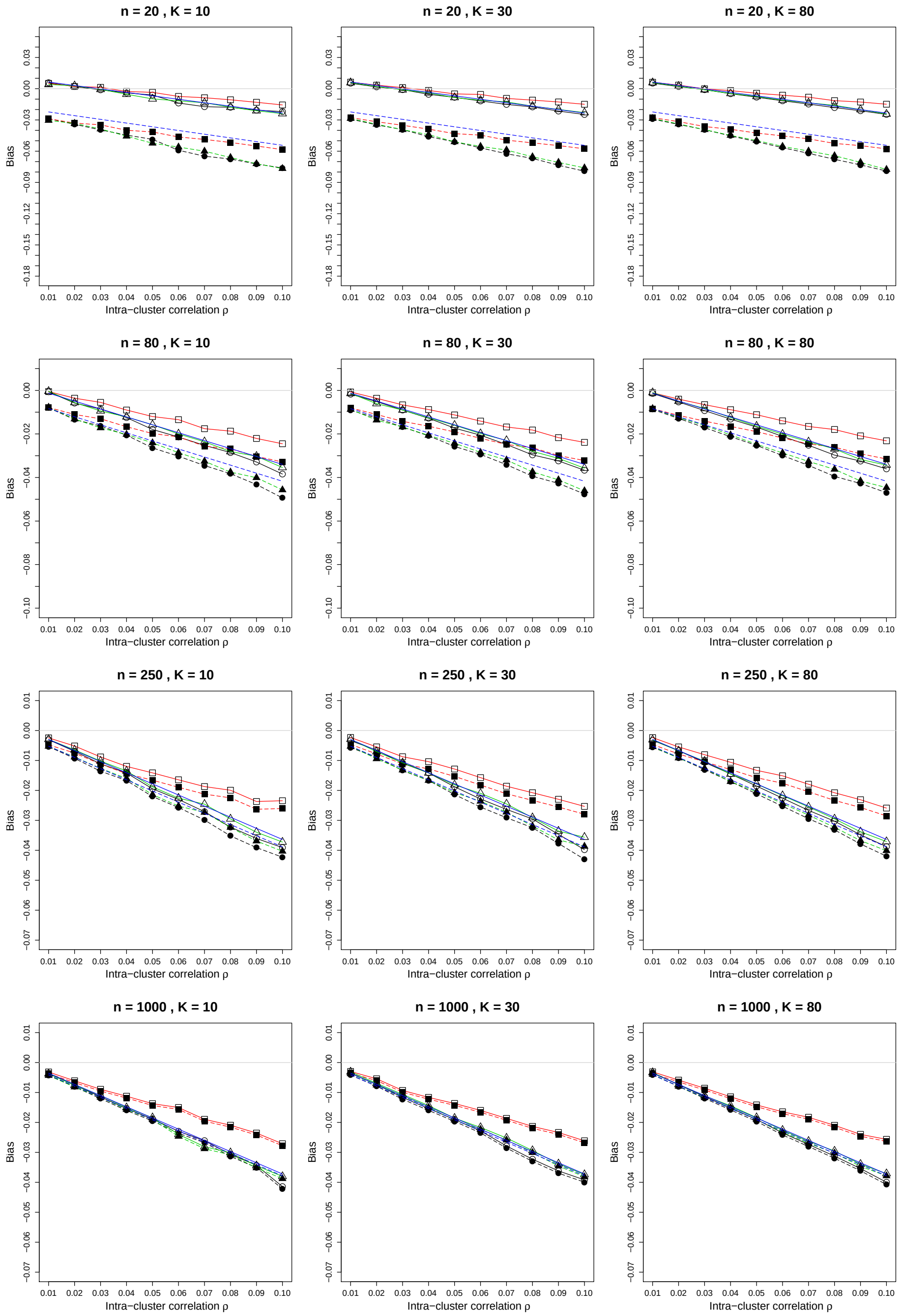

Figure A7 Bias on the arcsine scale of the meta-analysis of arcsine transformations from K studies in overdispersed binomial model for $p = 0.2$ and $0.01 \leq \rho \leq 0.1$. Simulations (10000 for each values of ρ) from beta-binomial distribution (circles), from Lunn and Davies (1998) model (squares) and from Gaussian copula of Emrich and Piedmonte (1991) (triangles) with and without the Anscombe (1948) correction (solid and dashed lines, respectively). Also the first order bias terms given by the first two terms of equation (4) and plotted for known p and ρ (solid or dashed blue lines). Light grey line at zero.

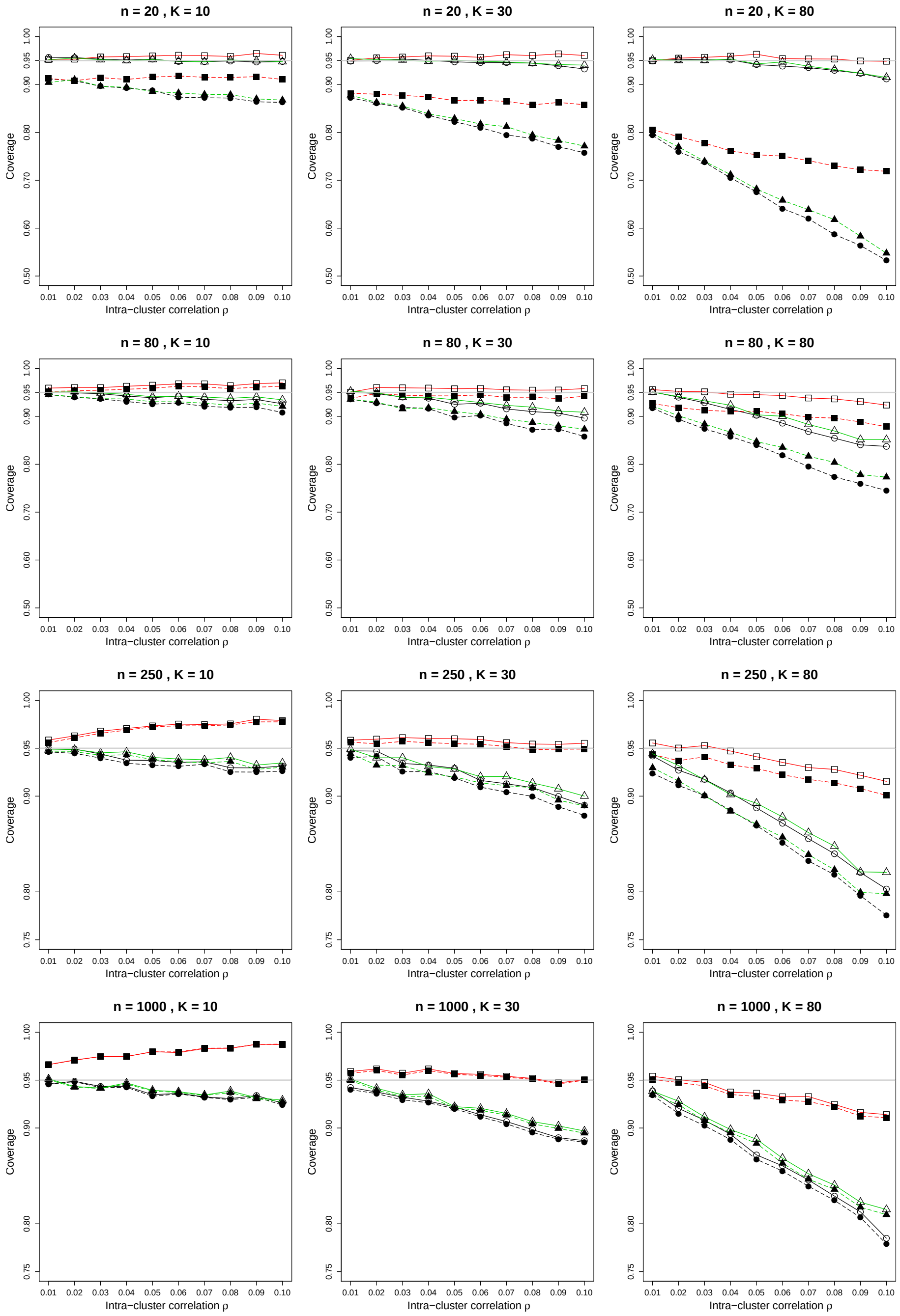

Figure A8 Coverage (for a known value of ρ) at the nominal 95% level of the true value of p using the meta-analysis of arcsine transformation from K studies in overdispersed binomial model for $p = 0.2$ and $0.01 \leq \rho \leq 0.1$. Simulations (10000 for each values of ρ) from beta-binomial distribution (circles), from Lunn and Davies (1998) model (squares) and from Gaussian copula of Emrich and Piedmonte (1991) (triangles) with and without the Anscombe (1948) correction (solid or dashed lines, respectively). Light grey line at 0.95.

© 0 WILEY-VCH Verlag GmbH & Co. KGaA, Weinheim

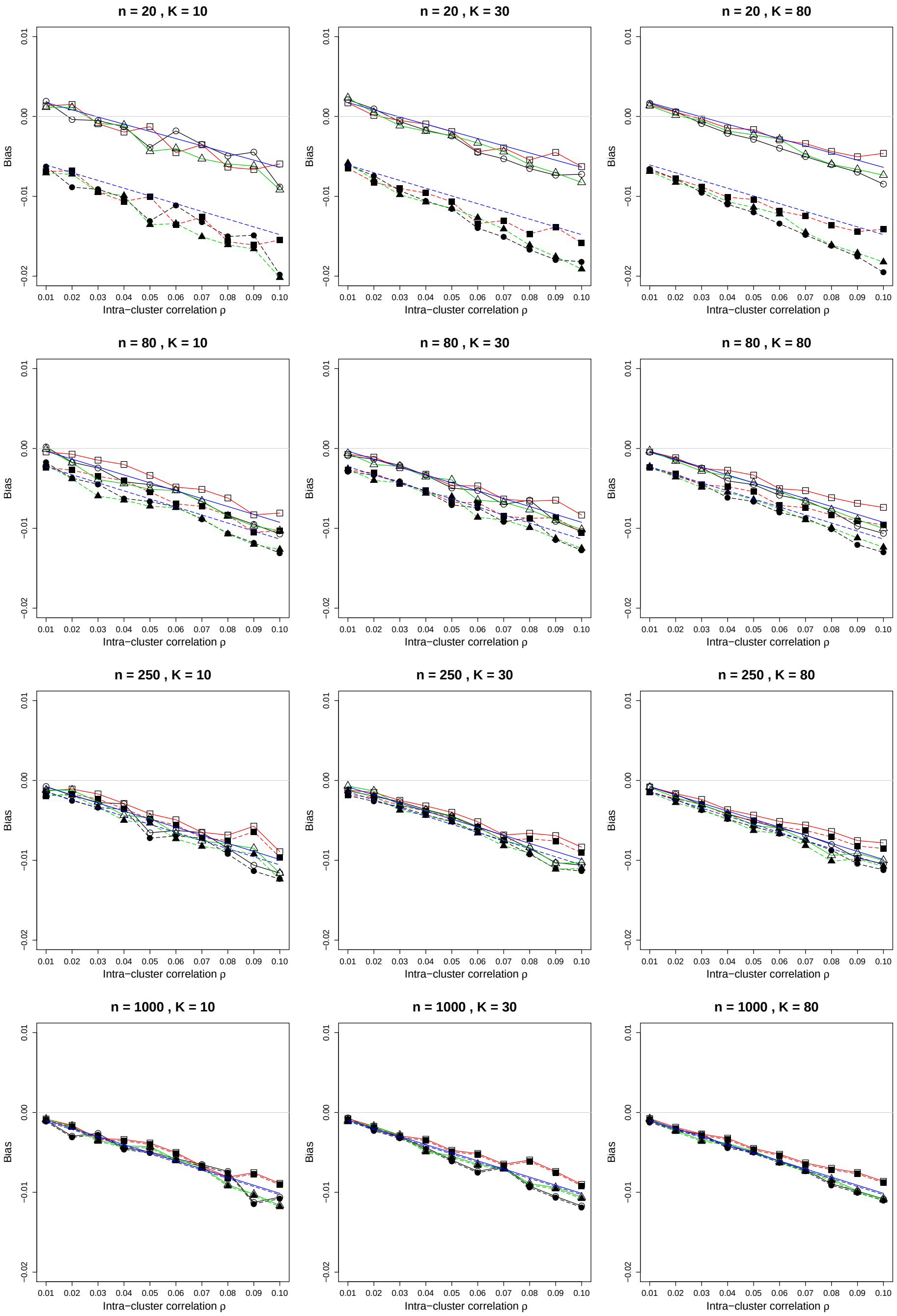

Figure A9 Bias on the arcsine scale of the meta-analysis of arcsine transformations from K studies in overdispersed binomial model for $p = 0.4$ and $0.01 \leq \rho \leq 0.1$. Simulations (10000 for each values of ρ) from beta-binomial distribution (circles), from Lunn and Davies (1998) model (squares) and from Gaussian copula of Emrich and Piedmonte (1991) (triangles) with and without the Anscombe (1948) correction (solid and dashed lines, respectively). Also the first order bias terms given by the first two terms of equation equation (4) and plotted for known p and ρ (solid or dashed blue lines). Light grey line at zero.

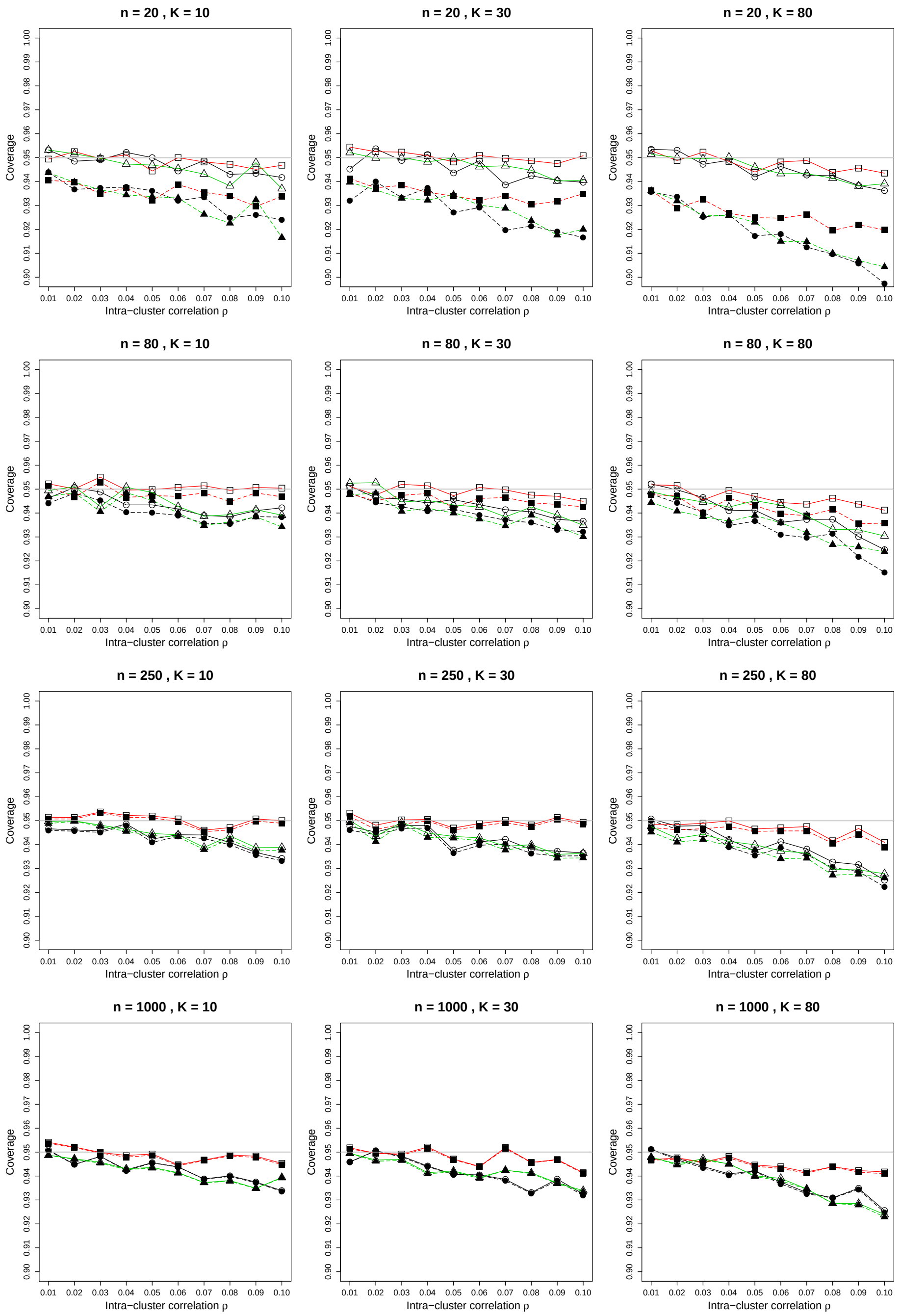

Figure A10 Coverage (for a known value of ρ) at the nominal 95% level of the true value of p using the meta-analysis of arcsine transformation from K studies in overdispersed binomial model for $p = 0.4$ and $0.01 \leq \rho \leq 0.1$. Simulations (10000 for each values of ρ) from beta-binomial distribution (circles), from Lunn and Davies (1998) model (squares) and from Gaussian copula of Emrich and Piedmonte (1991) (triangles) with and without the Anscombe (1948) correction (solid or dashed lines, respectively). Light grey line at 0.95.

© 0 WILEY-VCH Verlag GmbH & Co. KGaA, Weinheim

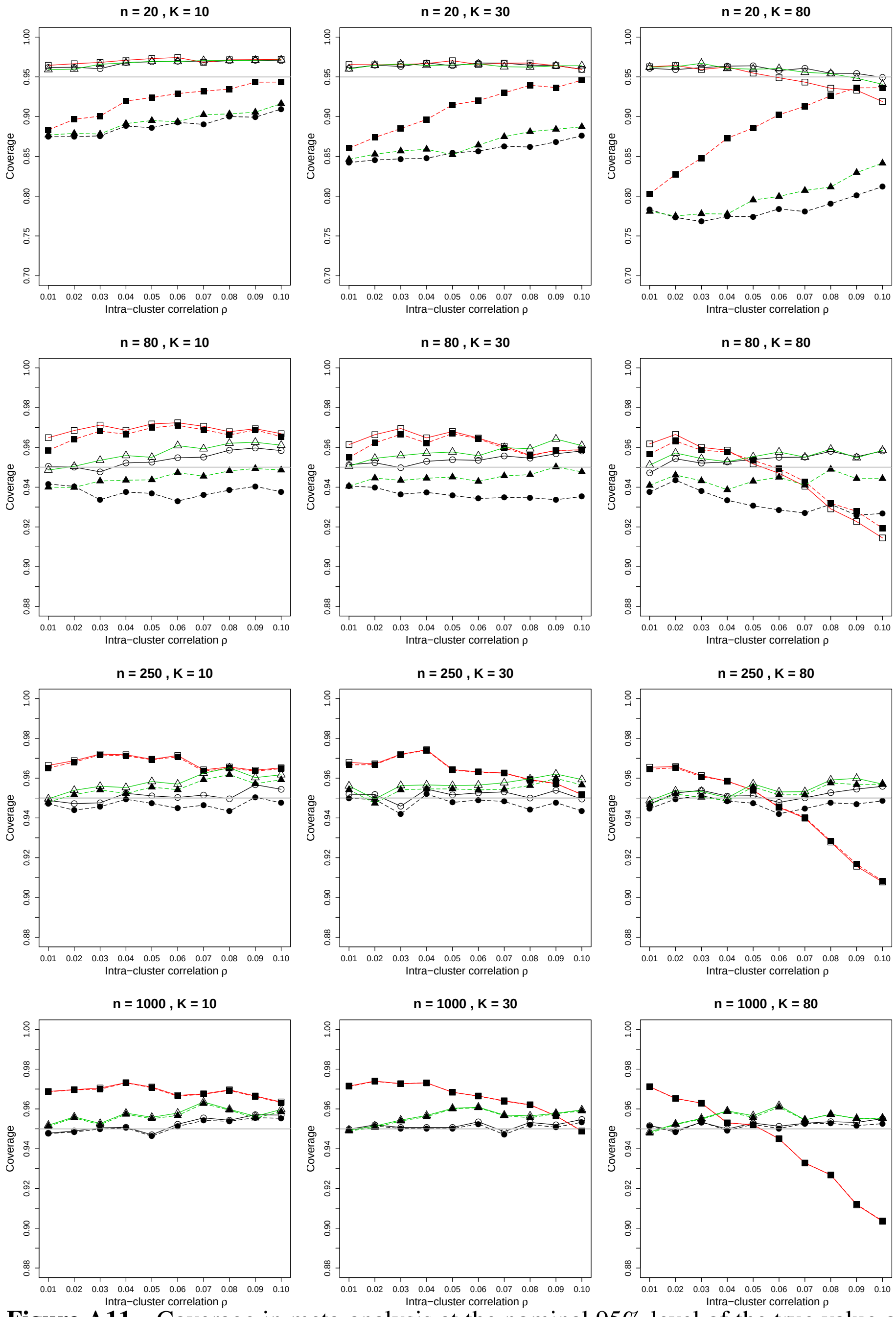

Figure A11 Coverage in meta-analysis at the nominal 95% level of the true value of p using the arcsine transformation with bias-correction in overdispersed binomial model for $p = 0.1$ and $0.01 \leq \rho \leq 0.1$; n sample size; k number of studies. Simulations (10000 for each values of ρ) from beta-binomial distribution (circles), from Lunn and Davies (1998) model (squares) and from model with Gaussian Copula of Emrich and Piedmonte (1991) (triangles) with and without the Anscombe (1948) correction (black, red and green colour, respectively). Light grey line at 0.95.

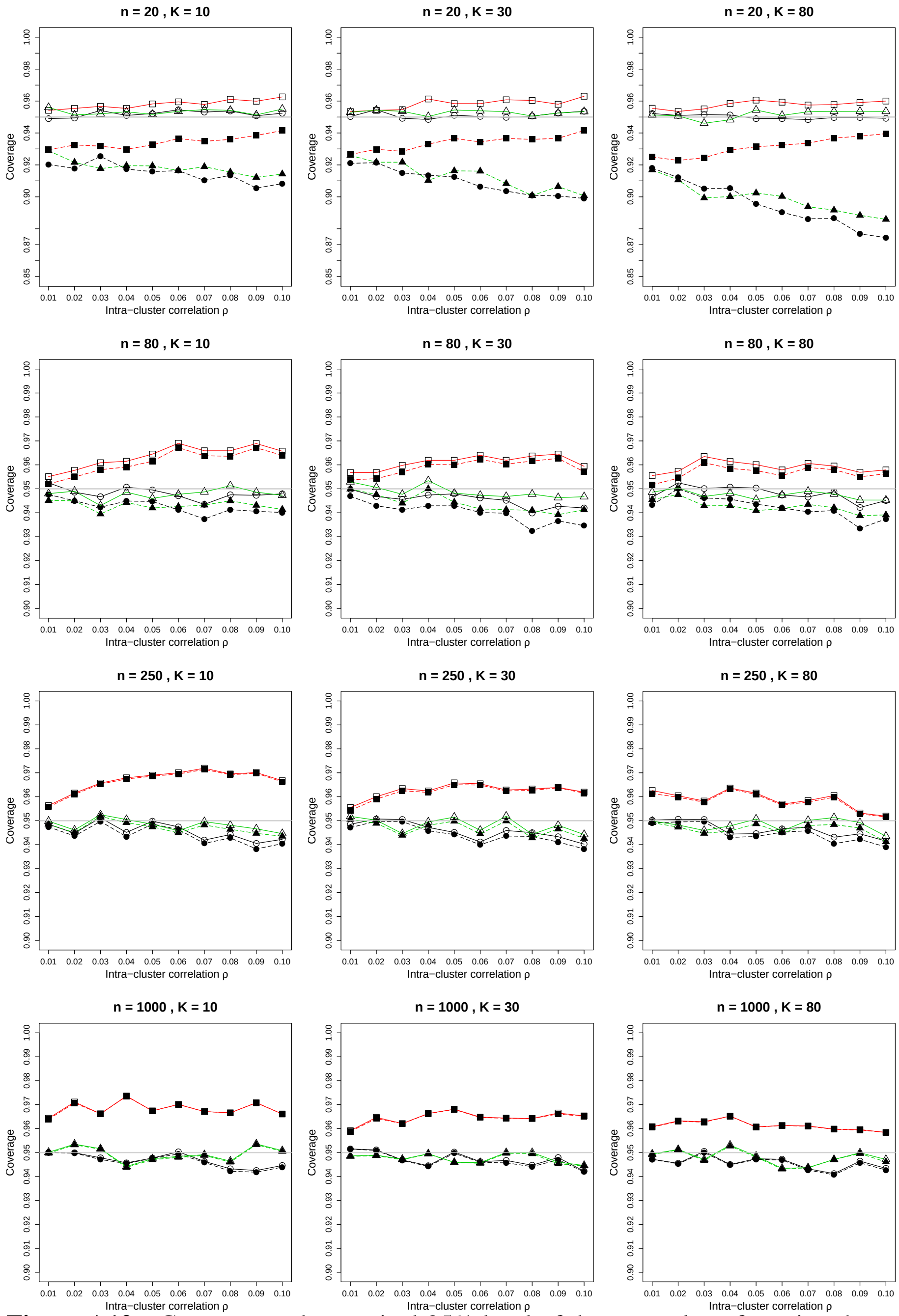

Figure A12 Coverage at the nominal 95% level of the true value of p using the acrsine transformation with bias-correction in overdispersed binomial model for $p = 0.2$ and $0.01 \leq \rho \leq 0.1$; n sample size; k number of studies. Simulations (10000 for each values of ρ) from beta-binomial distribution (circles), from Lunn and Davies (1998) model (squares) and from model with Gaussian Copula of Emrich and Piedmonte (1991) (triangles) with and without the Anscombe (1948) correction (black, red and green colour respectively). Light grey line at 0.95.

© 0 WILEY-VCH Verlag GmbH & Co. KGaA, Weinheim

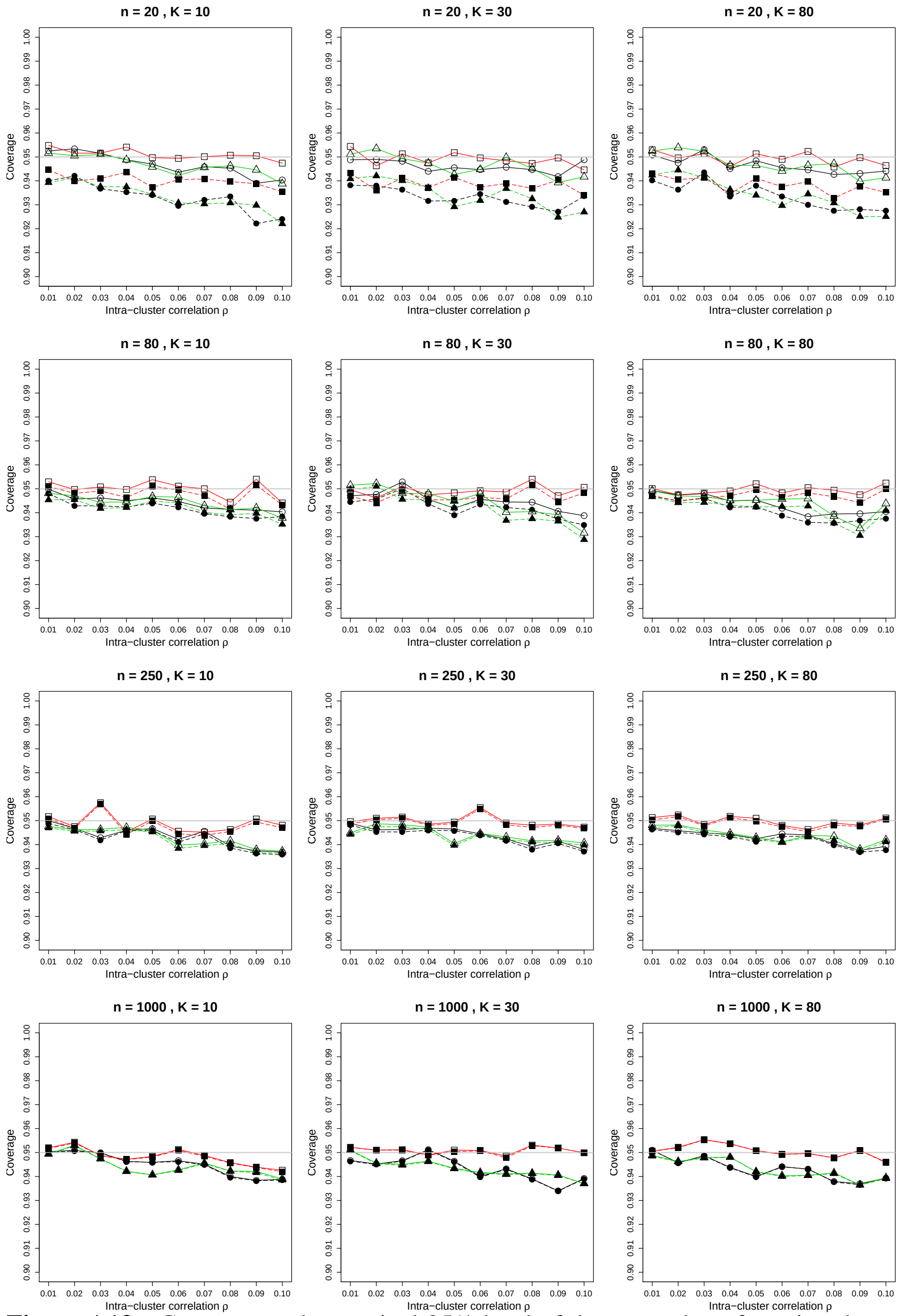

Figure A13 Coverage at the nominal 95% level of the true value of p using the acrsine transformation with bias-correction in overdispersed binomial model for $p = 0.4$ and $0.01 \leq \rho \leq 0.1$; n sample size; k number of studies. Simulations (10000 for each values of ρ) from beta-binomial distribution (circles), from Lunn and Davies (1998) model (squares) and from model with Gaussian Copula of Emrich and Piedmonte (1991) (triangles) with and without the Anscombe (1948) correction (black, red and green colour, respectively). Light grey line at 0.95.

© 0 WILEY-VCH Verlag GmbH & Co. KGaA, Weinheim

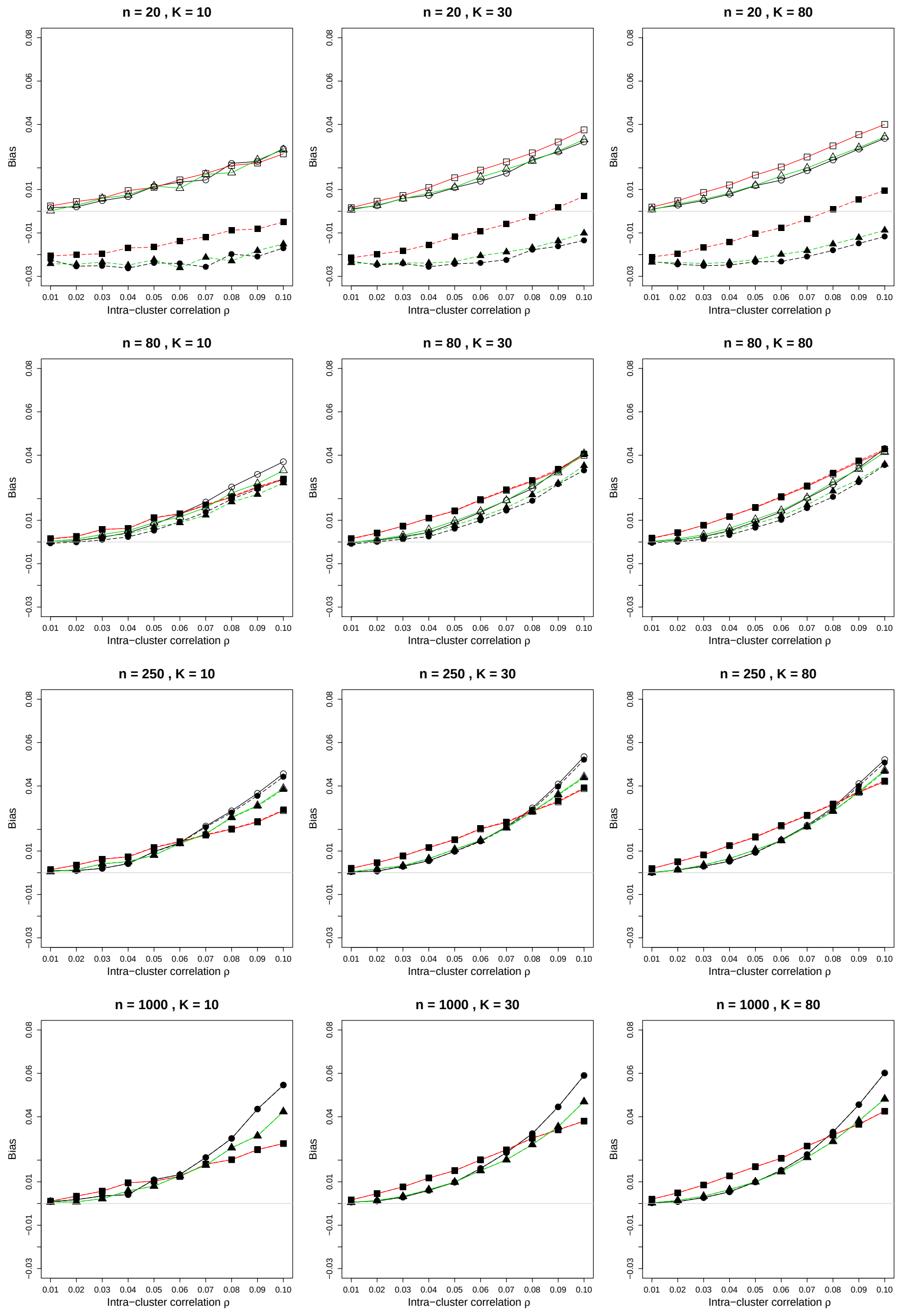

Figure A14 Bias on the arcsine scale in the meta-analysis of bias-corrected arcsine transformations from K studies in overdispersed binomial model for $p = 0.1$ and $0.01 \leq \rho \leq 0.1$ with estimated probabilities $\hat{p}_i$ and $\hat{\rho}_{AOV}$ in the bias correction terms. 10000 simulations for each value of ρ from the beta-binomial distribution (circles), from the Lunn and Davies (1998) model (squares) and from the GC model of Emrich and Piedmonte (1991) (triangles), with and without the Anscombe (1948) correction (solid and dashed lines, respectively). Light grey line at zero.

© 0 WILEY-VCH Verlag GmbH & Co. KGaA, Weinheim

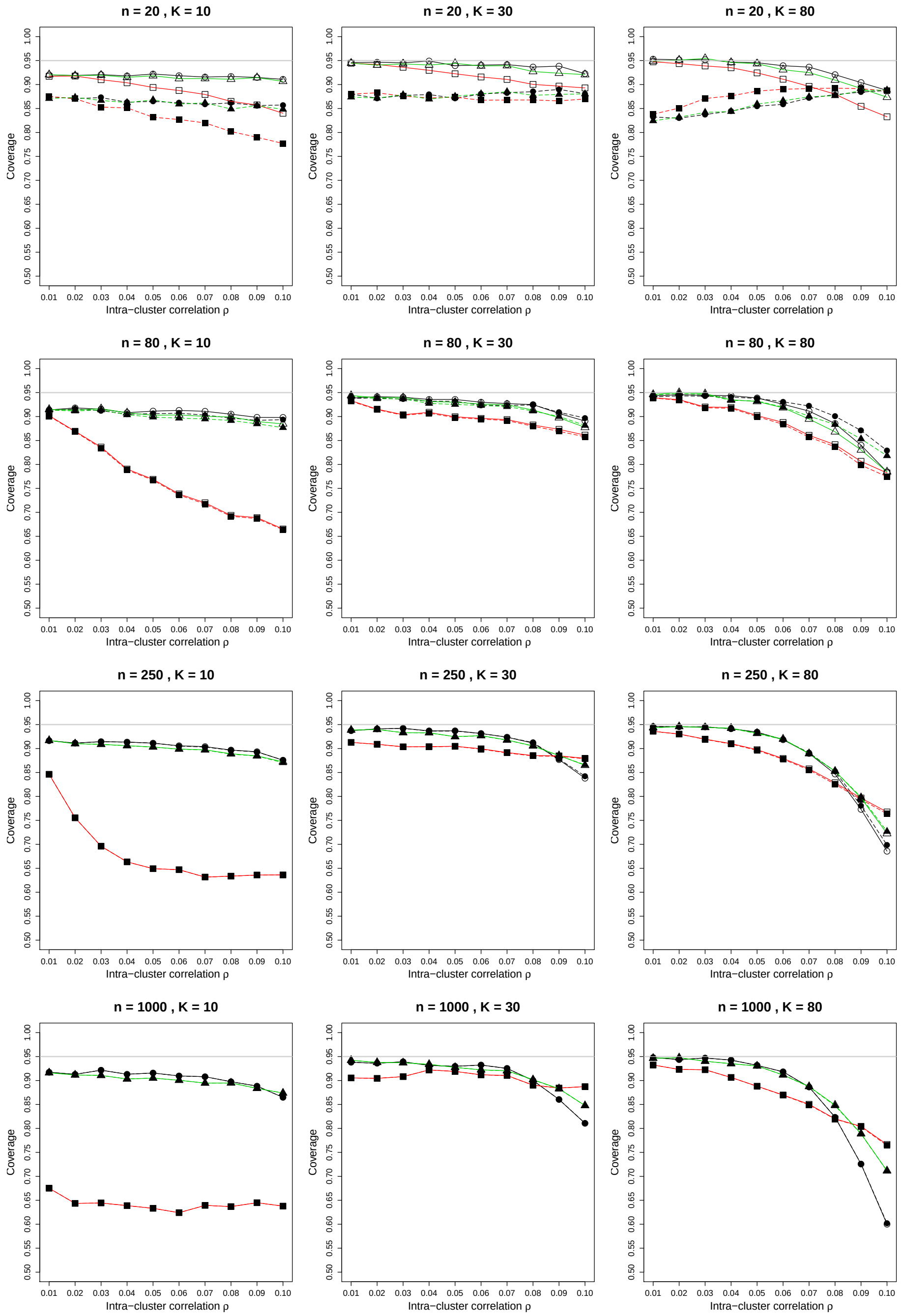

Figure A15 Coverage at the nominal 95% level of the true value of p in the meta-analysis of bias-corrected arcsine transformations from K studies in overdispersed binomial model for $p = 0.1$ and $0.01 \leq \rho \leq 0.1$ with estimated probabilities $\hat{p}_i$ and $\hat{\rho}_{AOV}$ in the bias correction terms. 10000 simulations for each value of ρ from the beta-binomial distribution (circles), from the Lunn and Davies (1998) model (squares) and from the GC model of Emrich and Piedmonte (1991) (triangles), with and without the Anscombe (1948) correction (solid and dashed lines, respectively). Light grey line at 0.95.

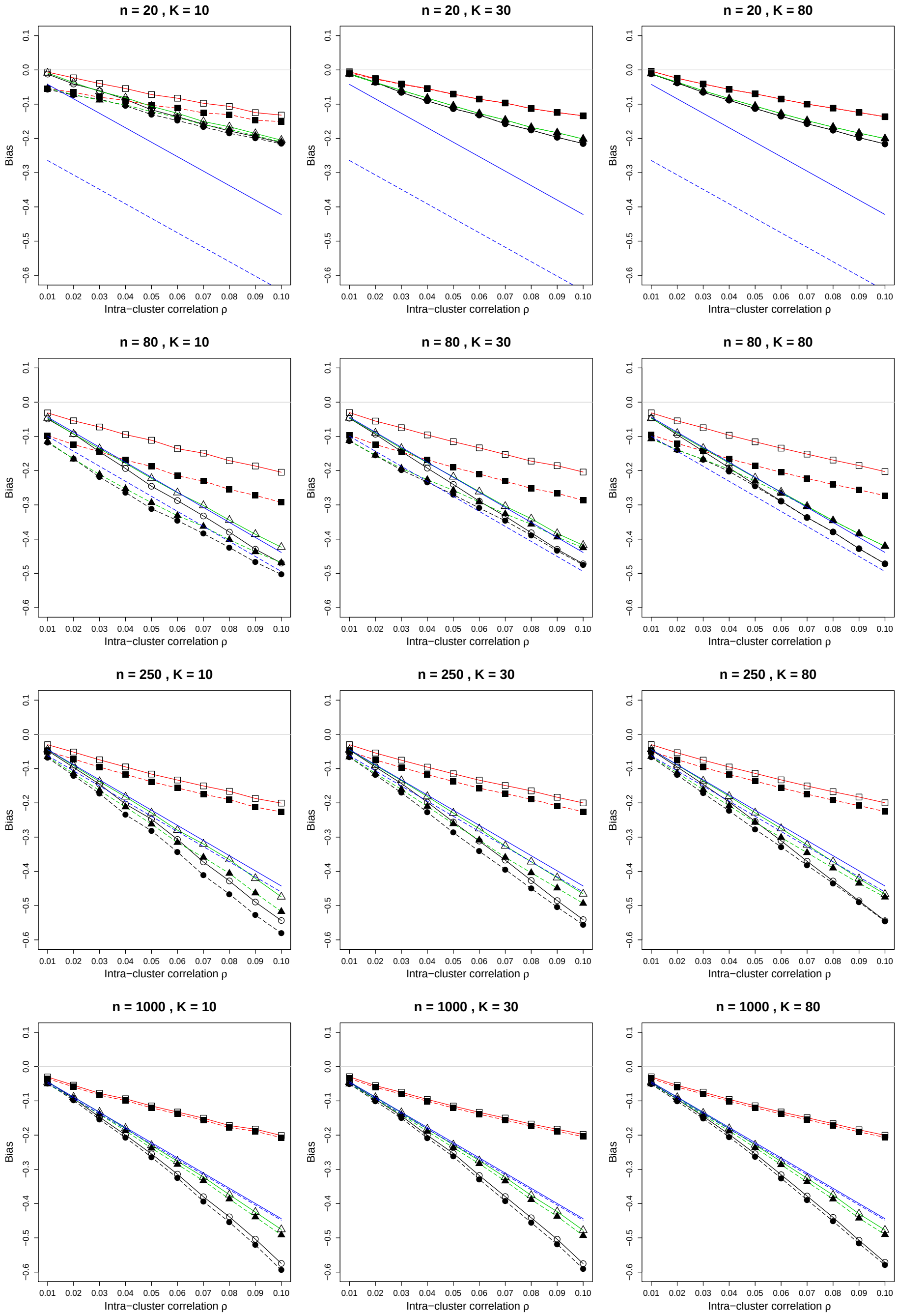

Figure A16 Bias on log-odds scale in the meta-analysis of log-odds from K studies in overdispersed binomial model for $p = 0.1$ $(\log(p/(1-p)) = -2.20)$ and $0.01 \leq \rho \leq 0.1$ with known p and ρ in the weights. Simulations (10000 for each values of ρ) from the beta-binomial distribution (circles); from the Lunn and Davies (1998) model (squares); from the GC model of Emrich and Piedmonte (1991) (triangles); and the first order bias term given by the first two terms of equation equation (4) and plotted for known values of p and ρ (blue) with and without the Gart et al. (1985) correction (solid and dashed lines, respectively). Light grey line at zero.

© 0 WILEY-VCH Verlag GmbH & Co. KGaA, Weinheim

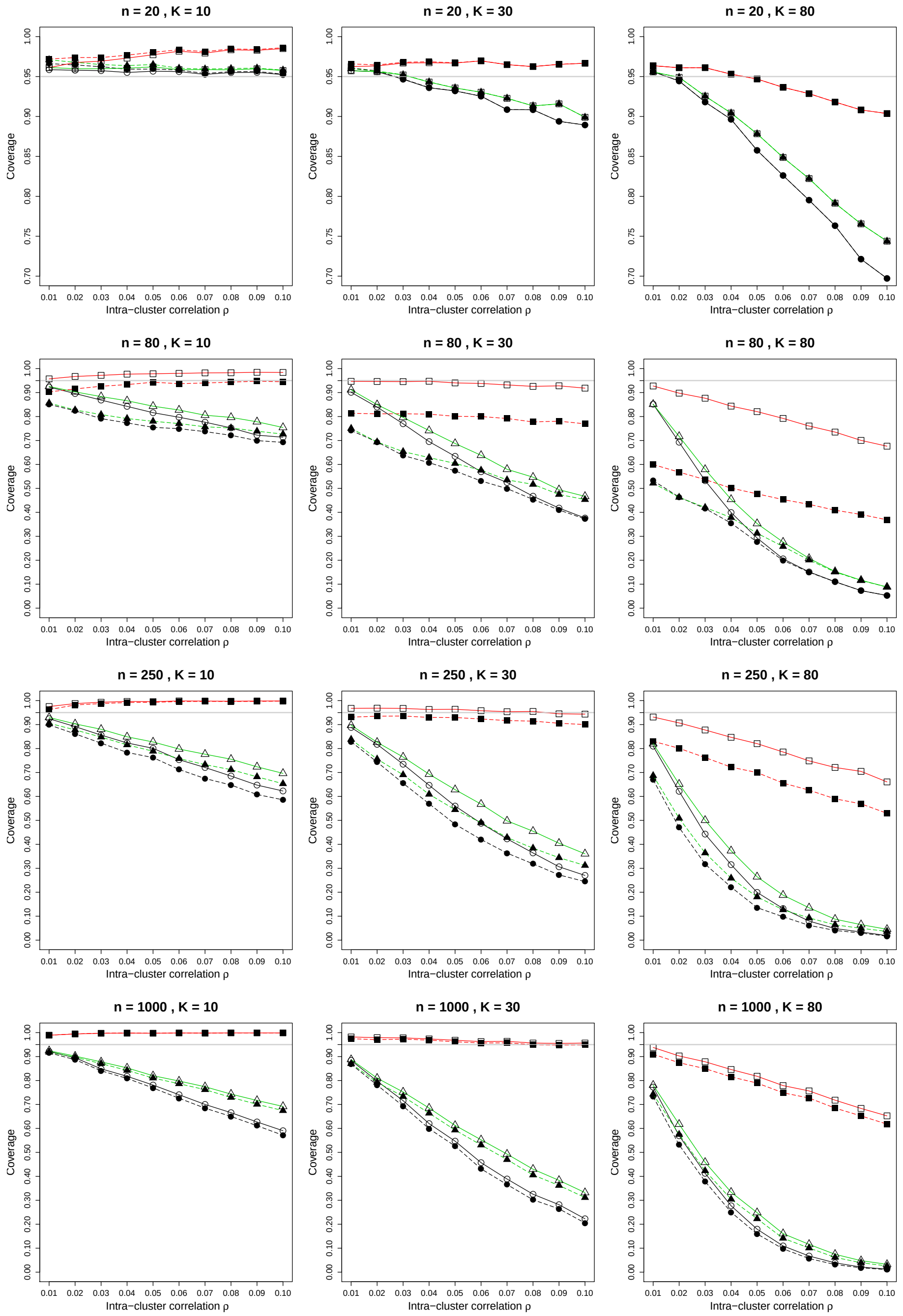

Figure A17 Coverage of the combined effect on log-odds scale in the meta-analysis of log-odds from K studies in overdispersed binomial model for $p = 0.1$ ($\log(p/(1-p)) = -2.20$) and $0.01 \leq \rho \leq 0.1$ using known p and ρ in the weights. 10000 simulations for each value of ρ from the beta-binomial distribution (circles); from the Lunn and Davies (1998) model (squares) and from the GC model of Emrich and Piedmonte (1991) (triangles), with and without the Gart et al. (1985) correction (solid and dashed lines, respectively). Light grey line at 0.95.

© 0 WILEY-VCH Verlag GmbH & Co. KGaA, Weinheim

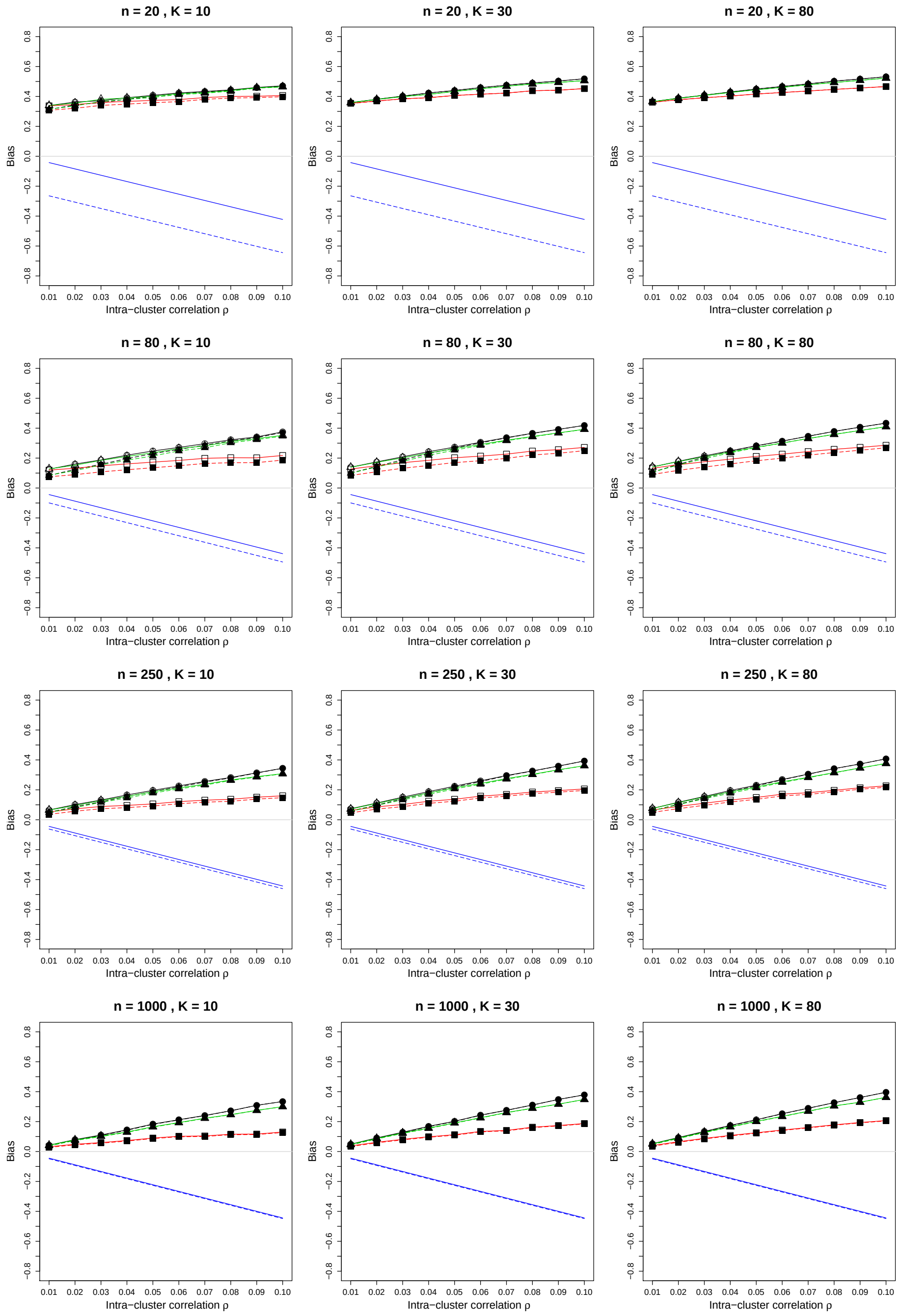

Figure A18 Bias on log-odds scale in the meta-analysis of log-odds from K studies in overdispersed binomial model for $p = 0.1$ ($\log(p/(1-p)) = -2.20$) and $0.01 \leq \rho \leq 0.1$ using estimated p and known ρ in the weights. 10000 simulations for each value of ρ from the beta-binomial distribution (circles); from the Lunn and Davies (1998) model (squares); from the GC model of Emrich and Piedmonte (1991) (triangles) and the first order bias term given by the first two terms of equation (equation (4)) and plotted for known values of p and ρ (blue), with and without the Gart et al. (1985) correction (solid and dashed lines, respectively). Light grey line at zero.

© 0 WILEY-VCH Verlag GmbH & Co. KGaA, Weinheim

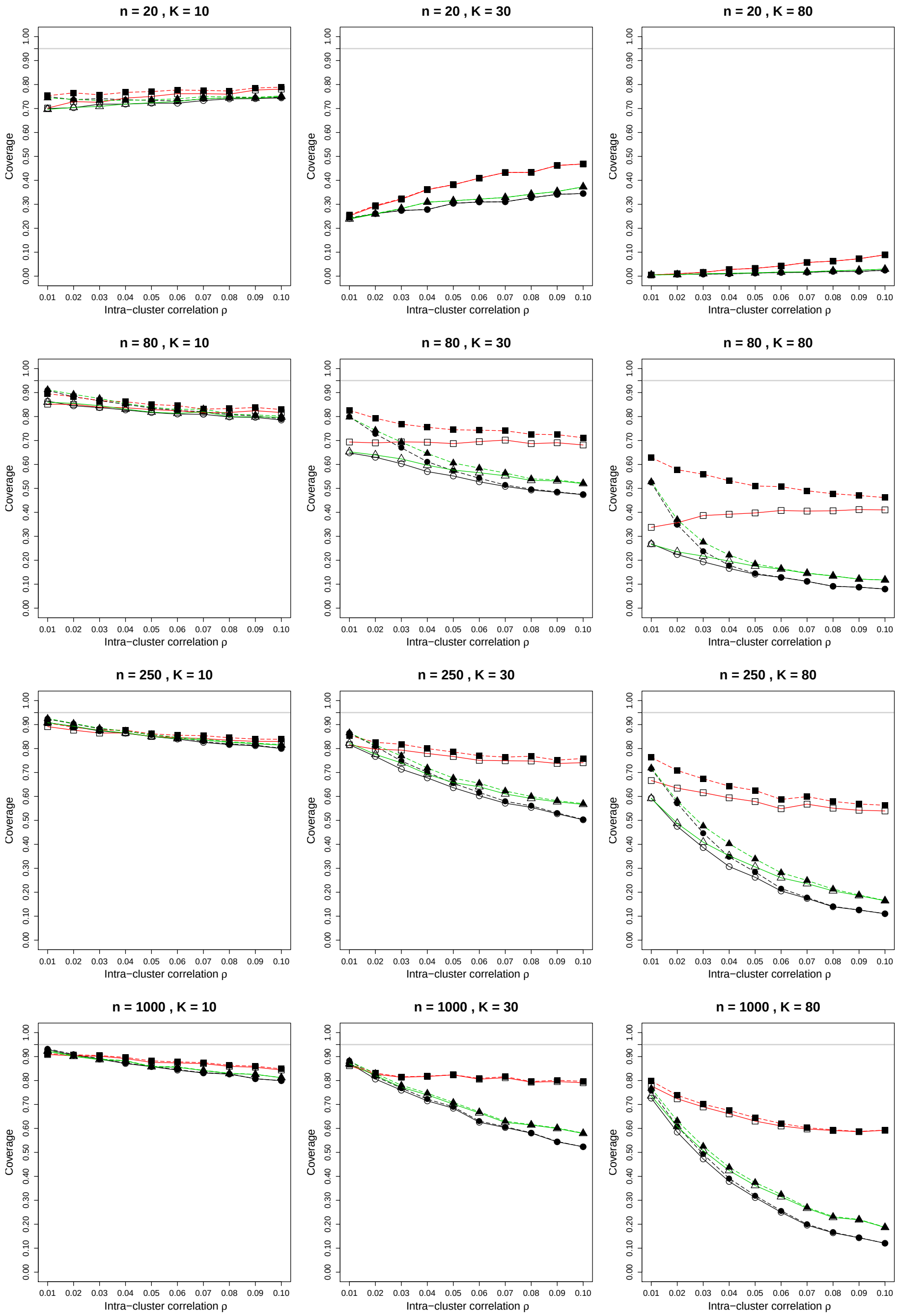

Figure A19 Coverage of the combined effect on log-odds scale in the meta-analysis of log-odds from K studies in overdispersed binomial model for $p = 0.1$ $(\log(p/(1-p)) = -2.20)$ and $0.01 \leq \rho \leq 0.1$ using estimated p and known ρ in the weights. 10000 simulations for each value of ρ from the beta-binomial distribution (circles); from the Lunn and Davies (1998) model (squares) and from the GC model of Emrich and Piedmonte (1991) (triangles), with and without the Gart et al. (1985) correction (solid and dashed lines, respectively). Light grey line at 0.95.

© 0 WILEY-VCH Verlag GmbH & Co. KGaA, Weinheim

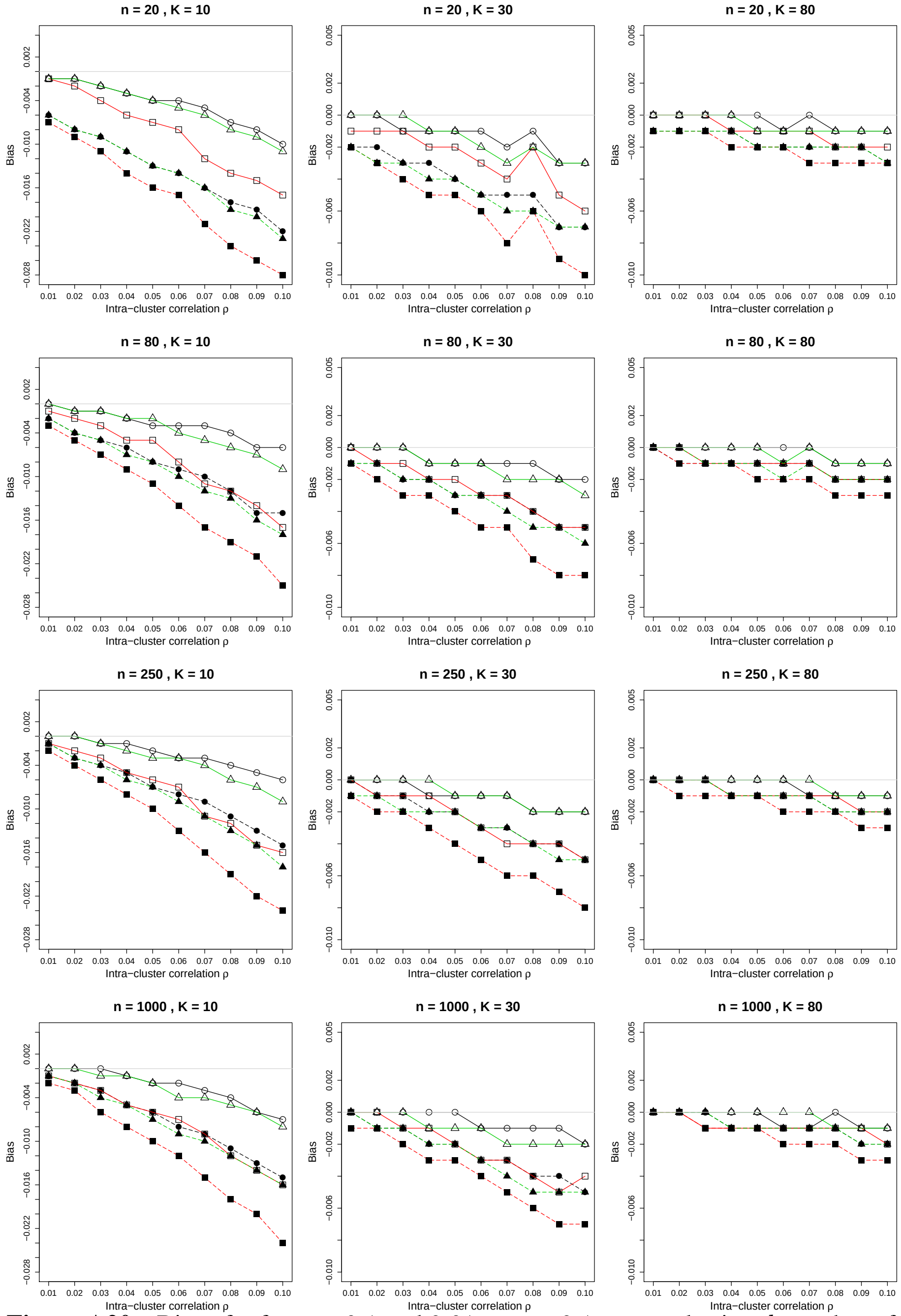

Figure A20 Bias of ρ for $p = 0.1$ and $0.01 \leq \rho \leq 0.1$; n sample size; k number of studies. Simulations (10000 for each values of ρ) from beta-binomial distribution (circles), from Lunn and Davies (1998) model (squares) and from model with Gaussian Copula of Emrich and Piedmonte (1991) (triangles) with $\hat{\rho}_{AOV}$ and $\hat{\rho}_{PPR}$ (solid and dashed lines, respectively). Light grey line at 0.

© 0 WILEY-VCH Verlag GmbH & Co. KGaA, Weinheim

Study	No of events	No of participants	$\hat{p}$	**Var**$(\hat{p})$	2**arcsin**$(\sqrt{\hat{p}})$	**Var**(2**arcsin**$(\sqrt{\hat{p}})$)
Craven et al., 1987	8	99	0.0808	0.0008	0.5765	0.0101
Moura et al., 2006	21	244	0.0861	0.0003	0.5955	0.0041
Cohen et al., 2002	2	22	0.0909	0.0038	0.6126	0.0455
Preljevic et al., 2011	3	25	0.12	0.0042	0.7075	0.04
Jouet et al., 1994	5	40	0.125	0.0027	0.7227	0.025
Huang et al.,1995	15	107	0.1402	0.0011	0.7675	0.0093
Alsuwaida et al., 2006	4	26	0.1538	0.005	0.8061	0.0385
Preljevic et al., 2011	13	84	0.1548	0.0016	0.8086	0.0119
Chan et al., 2011	23	141	0.1631	0.001	0.8315	0.0071
Lowry et al., 1980	15	83	0.1807	0.0018	0.8782	0.012
Eltayeb et al., 2010	55	300	0.1833	0.0005	0.8849	0.0033
Wuerth et al., 2005	70	380	0.1842	0.0004	0.8872	0.0026
Birmele et al., 2012	53	238	0.2227	0.0007	0.9829	0.0042
Chilcot et al., 2008	9	40	0.225	0.0044	0.9884	0.025
Chen et al., 2010	47	200	0.235	0.0009	1.0122	0.005
Soykan et al., 2004	12	50	0.24	0.0036	1.0239	0.02
Hinrichsen et al., 1989	30	124	0.2419	0.0015	1.0285	0.0081
Kalender et al., 2007	11	42	0.2619	0.0046	1.0745	0.0238
Hedayati et al., 2006	26	98	0.2653	0.002	1.0822	0.0102
Drayer et al., 2006	17	62	0.2742	0.0032	1.1022	0.0161
Cukor et al., 2008	20	70	0.2857	0.0029	1.1279	0.0143
Kweon et al., 2011	15	50	0.3	0.0042	1.1593	0.02
Taskapan et al., 2003	9	30	0.3	0.007	1.1593	0.0333
Loosman et al., 2010	21	62	0.3387	0.0036	1.2423	0.0161
Hong et al., 2006	22	64	0.3438	0.0035	1.253	0.0156
Cruz et al., 2010	25	70	0.3571	0.0033	1.281	0.0143
Ceyhun et al., 2010	22	42	0.5238	0.0059	1.6184	0.0238
Koo et al., 2003	34	62	0.5484	0.004	1.6677	0.0161

Table A1 Data for Example 1: Prevalence of syndromal depression diagnosed by clinical interview with chronic kidney disease at the stage of dialysis , Palmer et al. (2013)

References

Anscombe, F. J. (1948). The transformation of Poisson, binomial and negative-binomial data. *Biometrika*, 35:246–254.

Demirtas, H., Hedeker, D., and Kapur, K. (2009). A comparative study on most commonly used correlated binary data generation methods. *Advances and Applications in Statistical Sciences*, 1(1):44–55.

Emrich, L. J. and Piedmonte, M. R. (1991). A method for generating high-dimensional multivariate binary variates. *The American Statistician*, 45(4):302–304.

Lunn, A. D. and Davies, S. J. (1998). A note on generating correlated binary variables. *Biometrika*, 85(2):487–490.

Madsen, L. and Birkes, D. (2013). Simulating dependent discrete data. *Journal of Statistical Computation and Simulation*, 83(4):677–691.

Qaqish, B. F., Zink, R. C., and Preisser, J. S. (2012). Orthogonalized residuals for estimation of marginally specified association parameters in multivariate binary data. *Scandinavian Journal of Statistics*, 39(3):515–527.

Rao Chaganty, N. and Joe, H. (2004). Efficiency of generalized estimating equations for binary responses. *Journal of the Royal Statistical Society: Series B (Statistical Methodology)*, 66(4):851–860.

© 0 WILEY-VCH Verlag GmbH & Co. KGaA, Weinheim

Country	Study	Study size	$\hat{p}$	Var$(\hat{p})$	2arcsin$(\sqrt{\hat{p}})$	Var(2arcsin$(\sqrt{\hat{p}})$)
USA	Zolopa et. al., 1994	1005	0.1	0.0000896	0.6435011	0.0009950
USA	Paris et. al., 1996	331	0.11	0.0002958	0.6761305	0.0030211
USA	Magura et. al., 2000	90	0.13	0.0012567	0.7377260	0.0111111
USA	Hahn et. al., 2004	639	0.01	0.0000155	0.2003348	0.0015649
USA	Robertson et. At., 2004	1958	0.12	0.0000539	0.7074832	0.0005107
France	Brouqui et al., 2005	848	0	0.0000000	0.0000000	0.0011792
USA	Grimpley et. al.,2006	285	0.01	0.0000347	0.2003348	0.0035088
Brazil	Brito et al, 2007	267	0.02	0.0000734	0.2837941	0.0037453
India	Talukdar et. Al, 2007	493	0.05	0.0000963	0.4510268	0.0020284
Sweden	Burstrom et, al, 2007	123	0.08	0.0005984	0.5735131	0.0081301
Sweden	Beijer, 2007	1757	0.02	0.0000112	0.2837941	0.0005692
USA	Forney et. Al. 2007	161	0.05	0.0002950	0.4510268	0.0062112
Iran	Vahdani et al 2009	2002	0.07	0.0000325	0.5355267	0.0004995
France	Laporte et al 2010	402	0.01	0.0000246	0.2003348	0.0024876
France	Colson et al 2011	220	0.01	0.0000450	0.2003348	0.0045455
USA	Wenzel et al 2011	305	0.08	0.0002413	0.5735131	0.0032787

Table A2 Data for Example 2: estimated prevalence of HIV infection in homeless people, Beijer et al. (2012)

© 0 WILEY-VCH Verlag GmbH & Co. KGaA, Weinheim
