# Supplementary figures and images for: Inference for binomial probability based on dependent Bernoulli random variables with applications to meta‐analysis and group level studies
Source: Biom J. 2016 May 18;58(4):896–914. doi: 10.1002/bimj.201500115 (PMC4999030; doi:10.1002/bimj.201500115)

**n = 40 , K = 1**

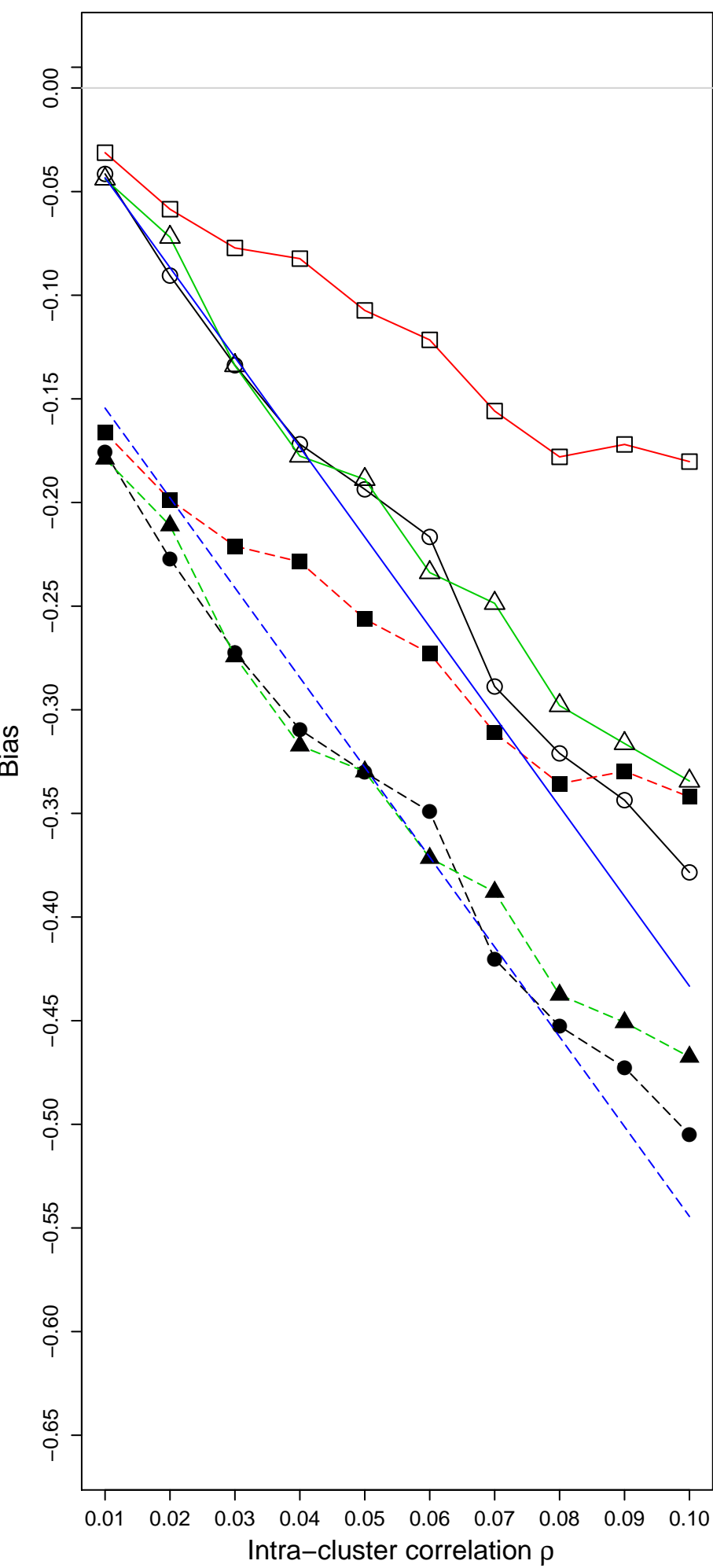

**n = 160 , K = 1**

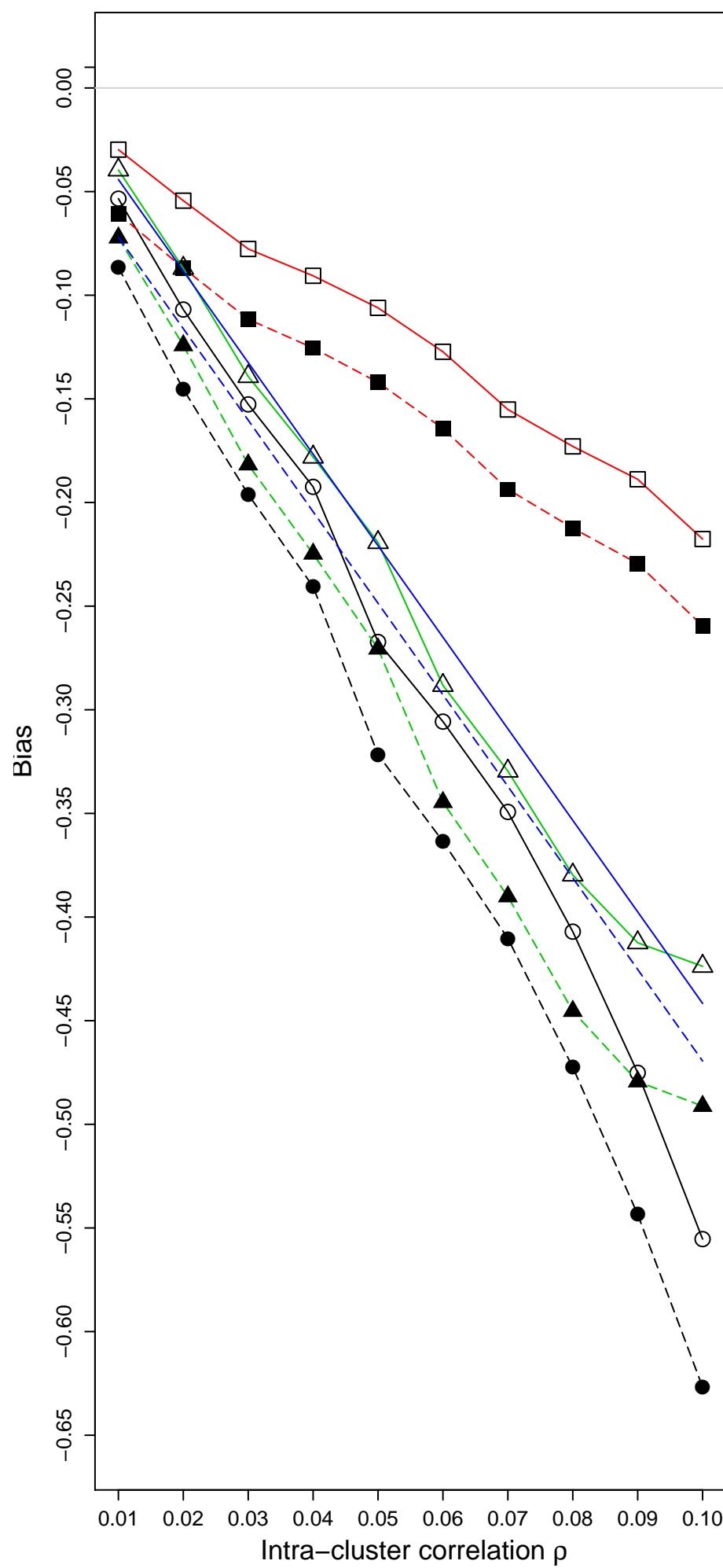

**n = 640 , K = 1**

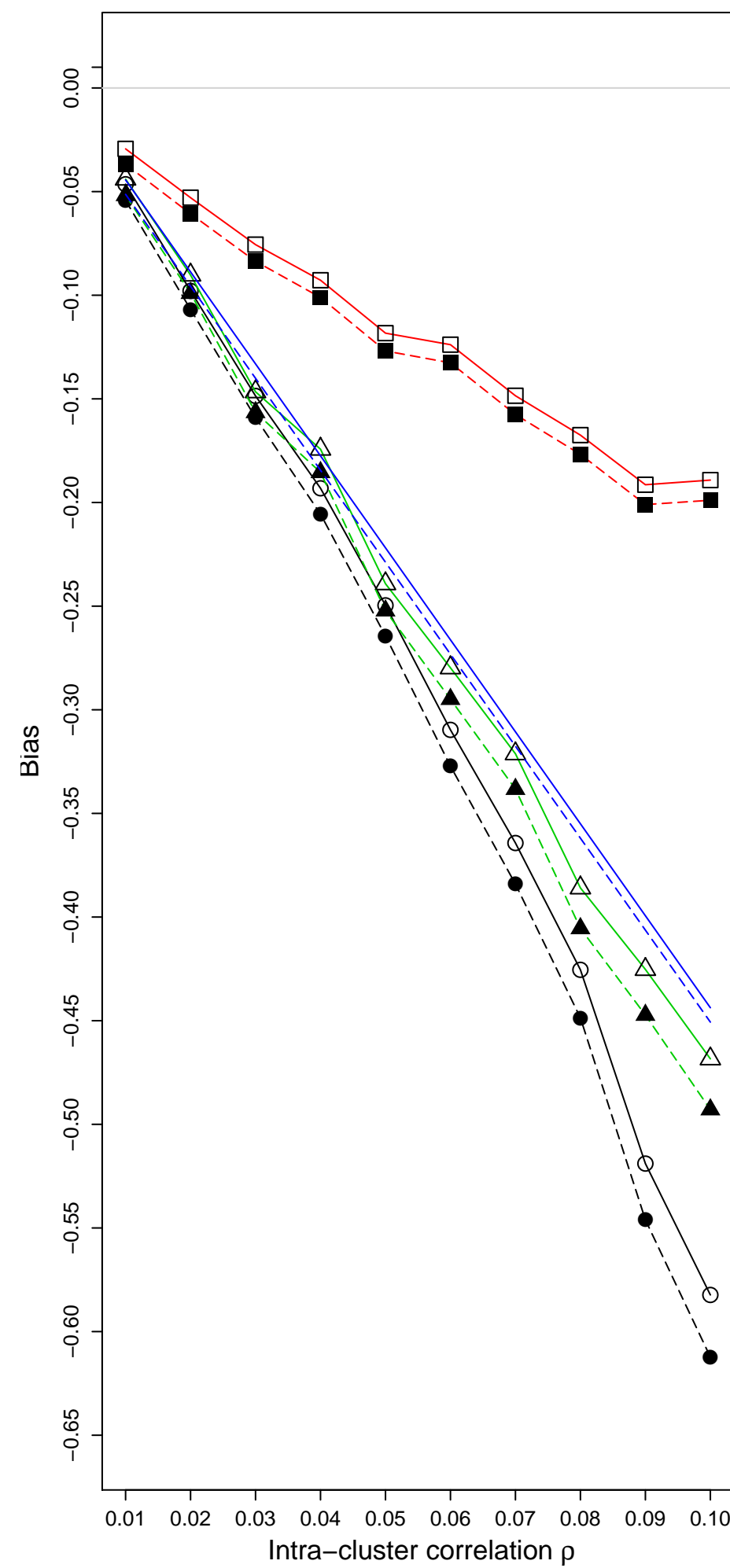

**n = 40 , K = 1**

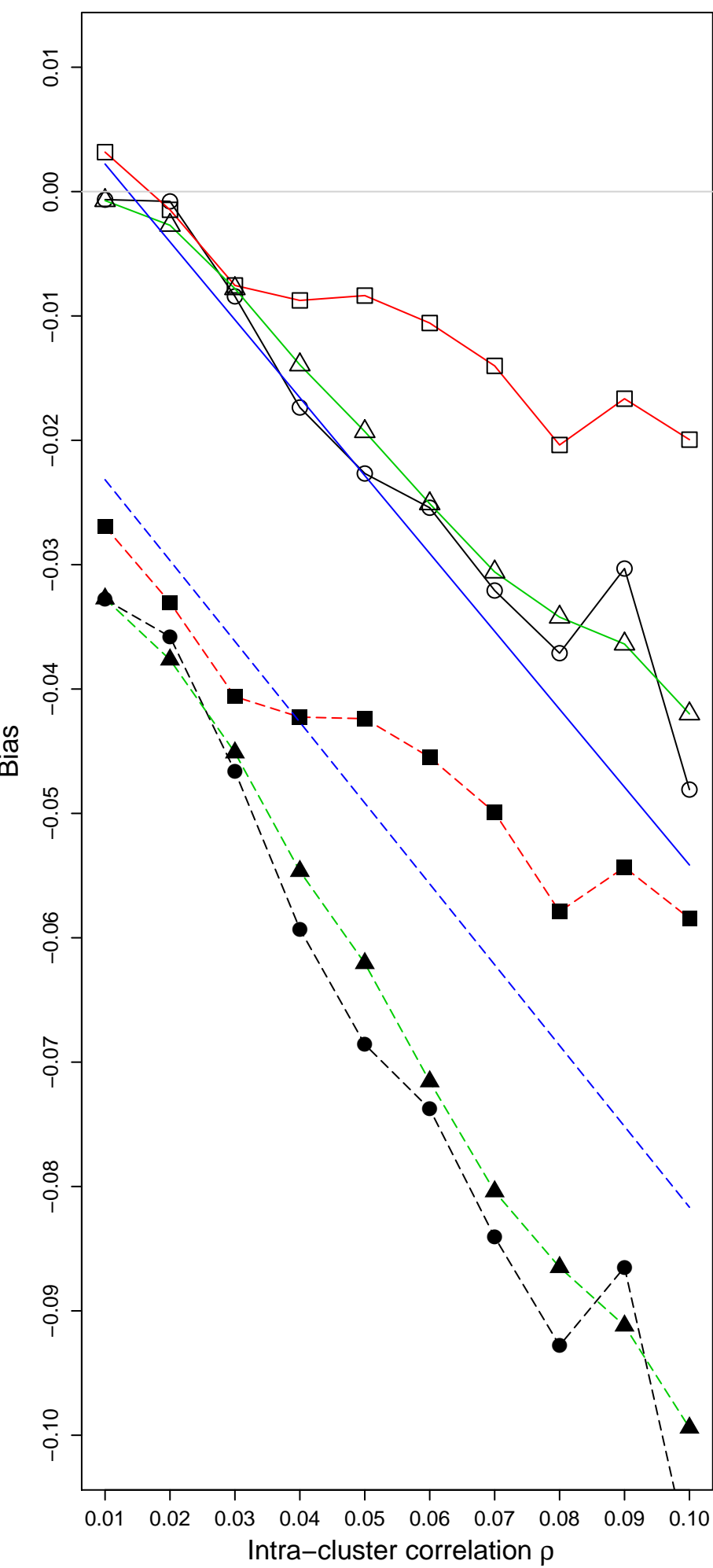

**n = 160 , K = 1**

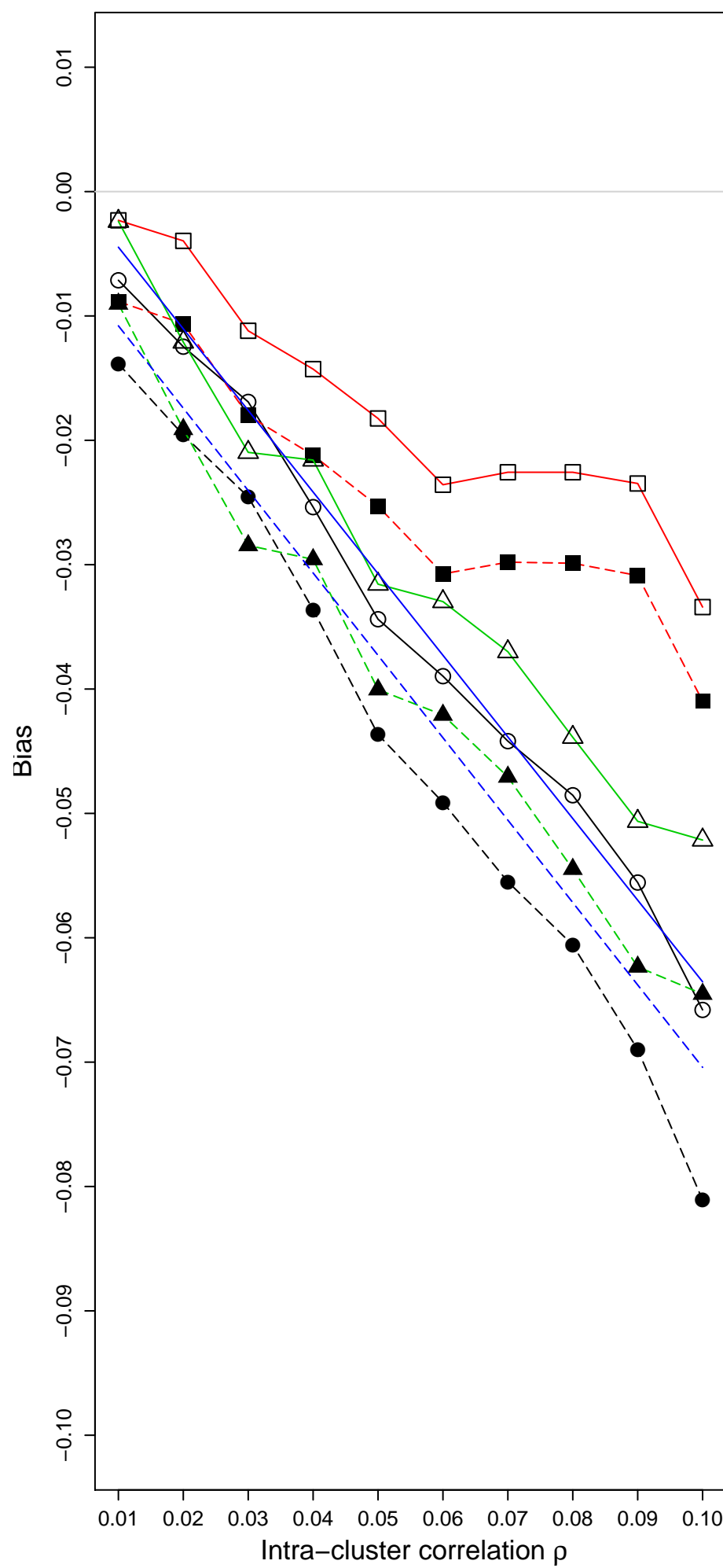

**n = 640 , K = 1**

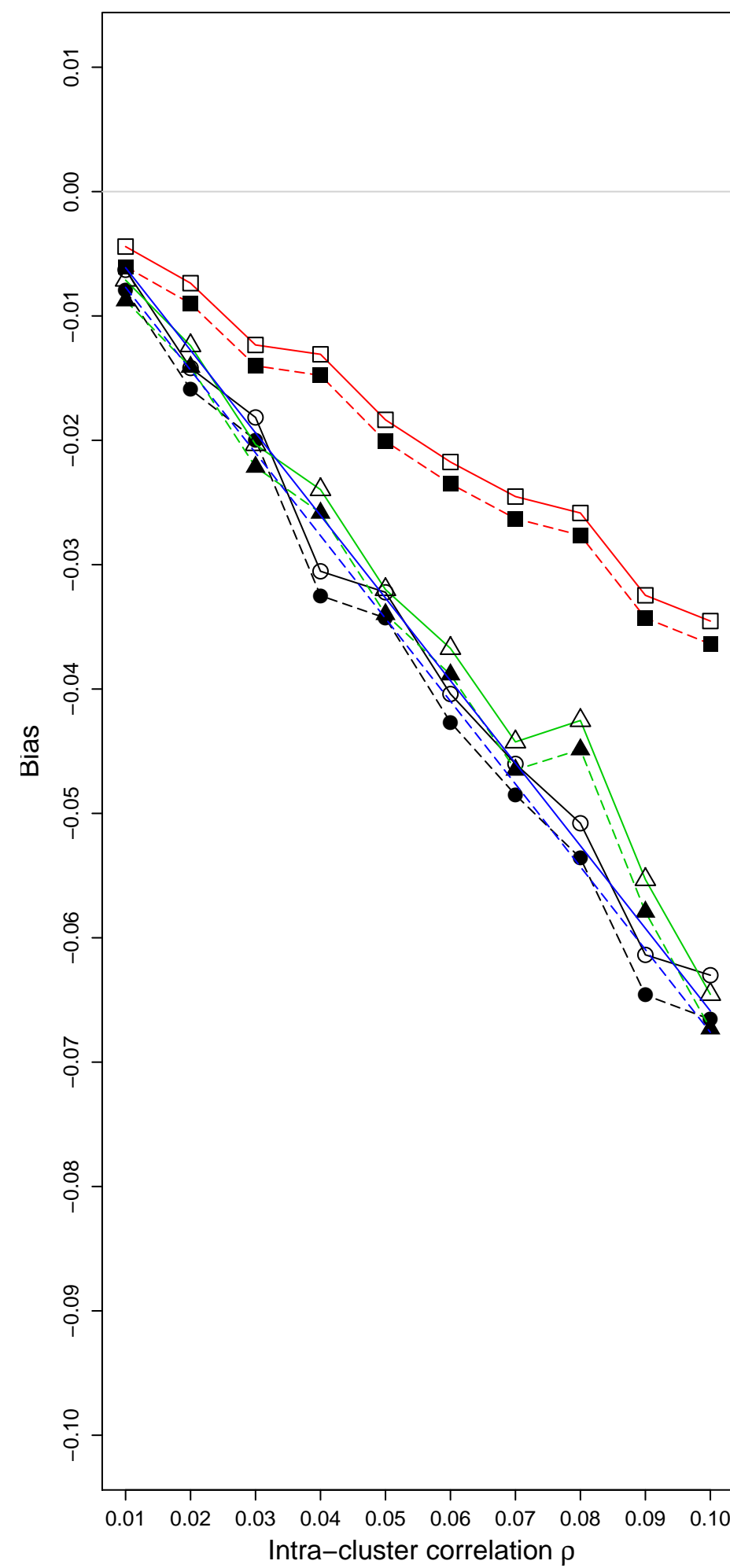

Supplement: Supplementary file 2 — Supporting Information [file BIMJ-58-896-s002.zip › README/FIGURE1/BiasPlotLogOddsAsinBBandLDandNCp01Kis1.pdf]

**n = 20 , K = 10**

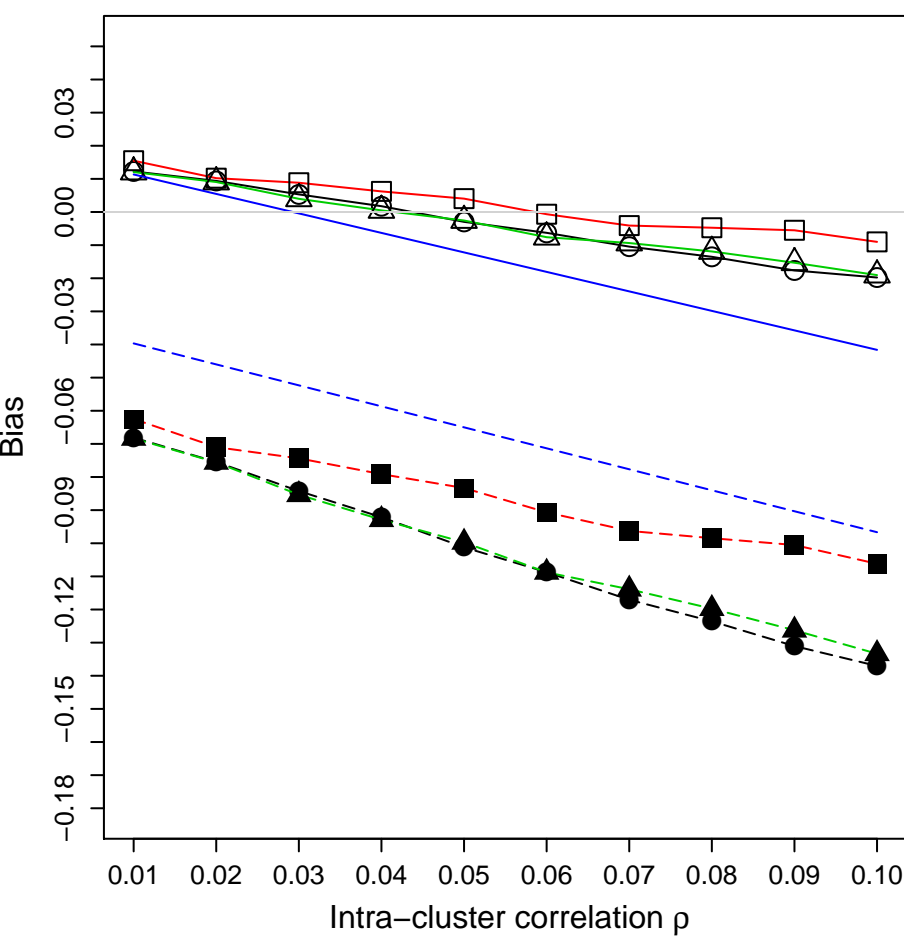

**n = 20 , K = 30**

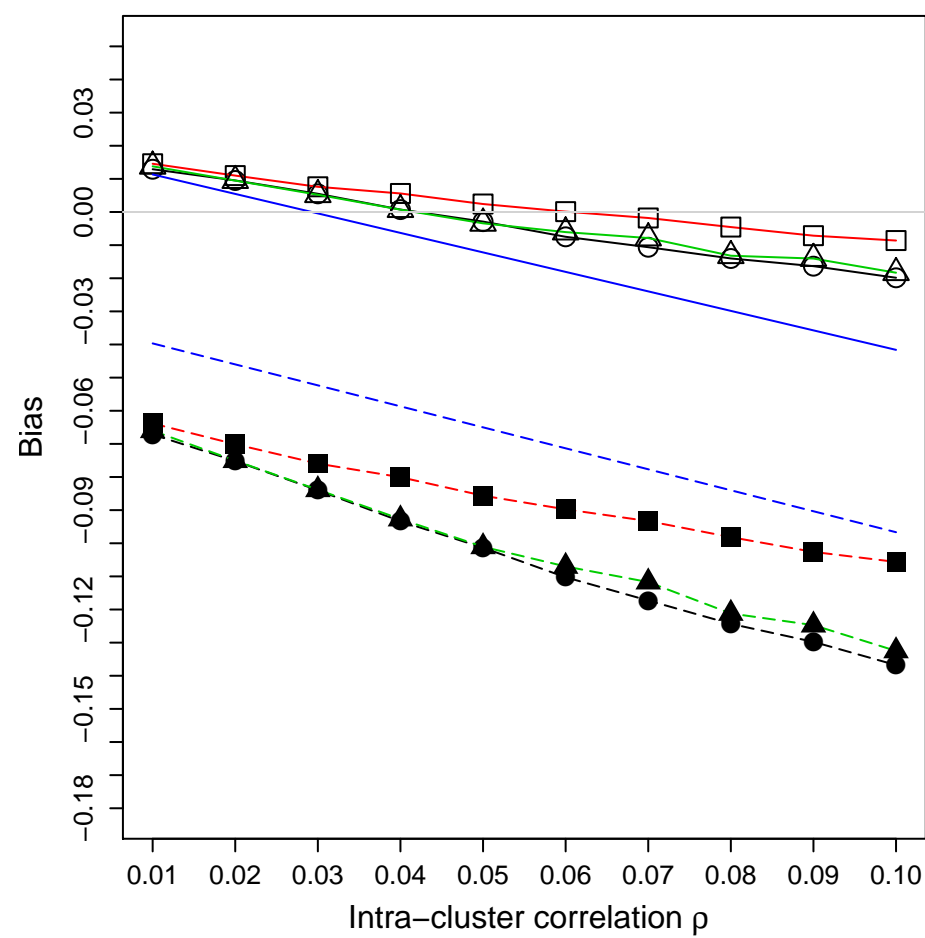

**n = 20 , K = 80**

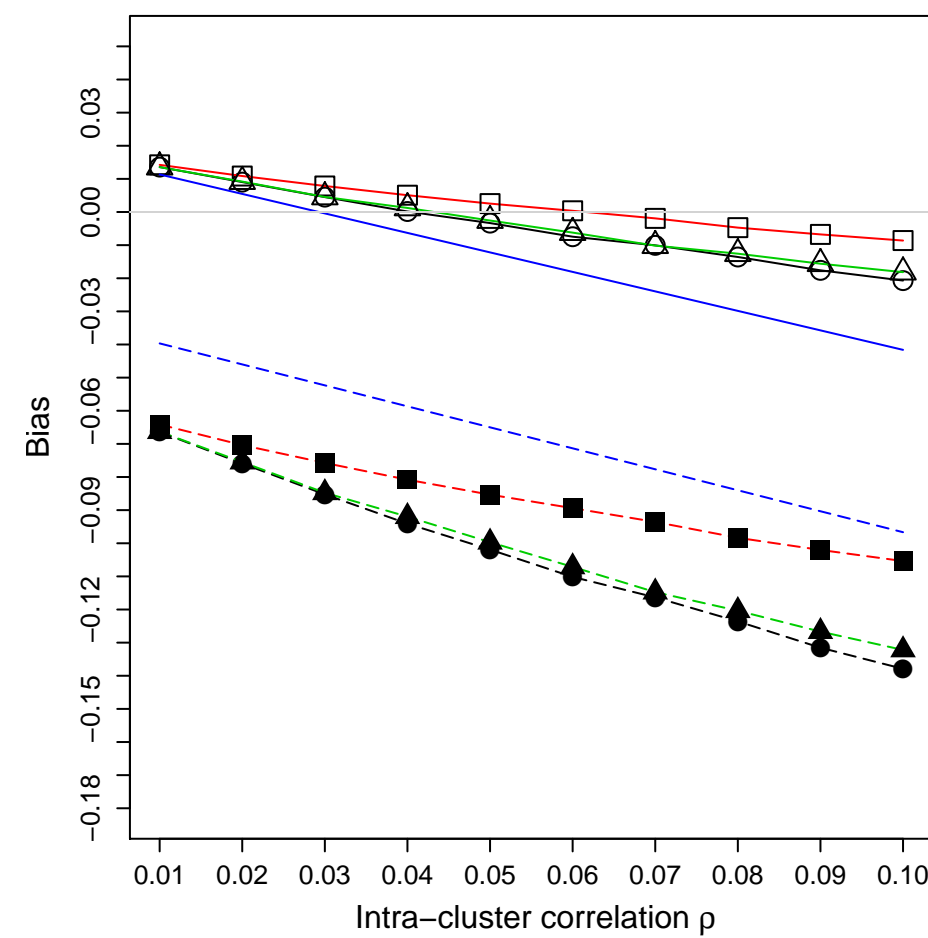

**n = 250 , K = 10**

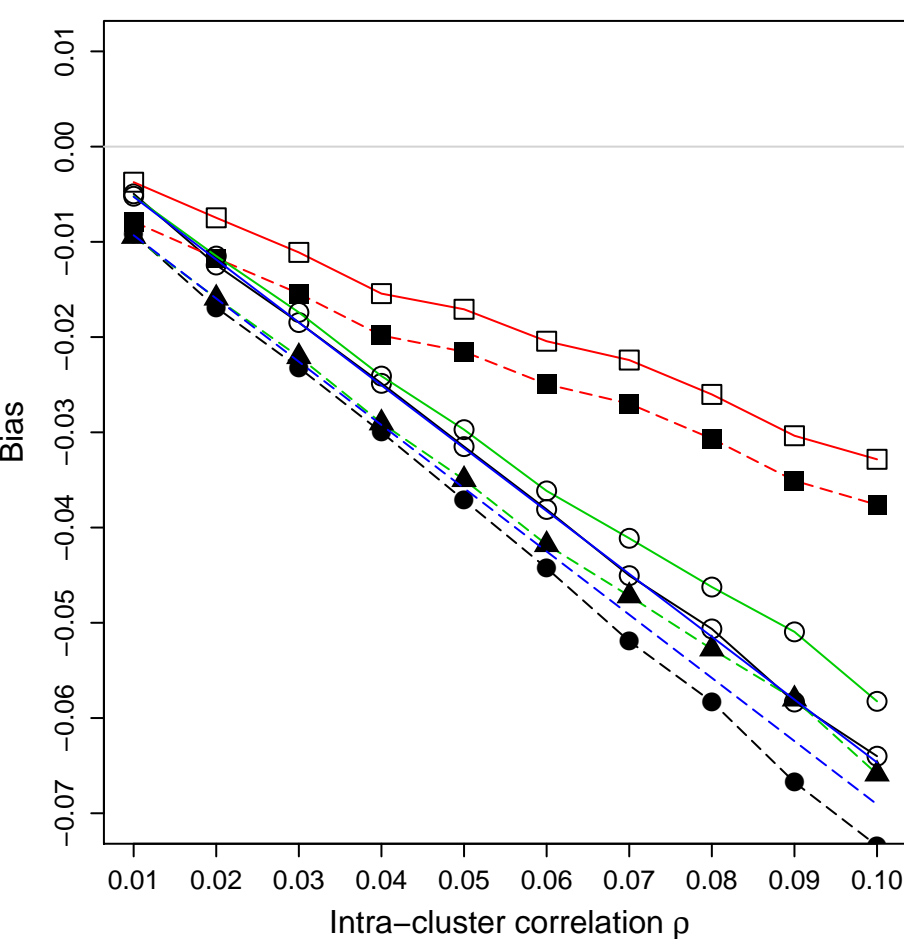

**n = 250 , K = 30**

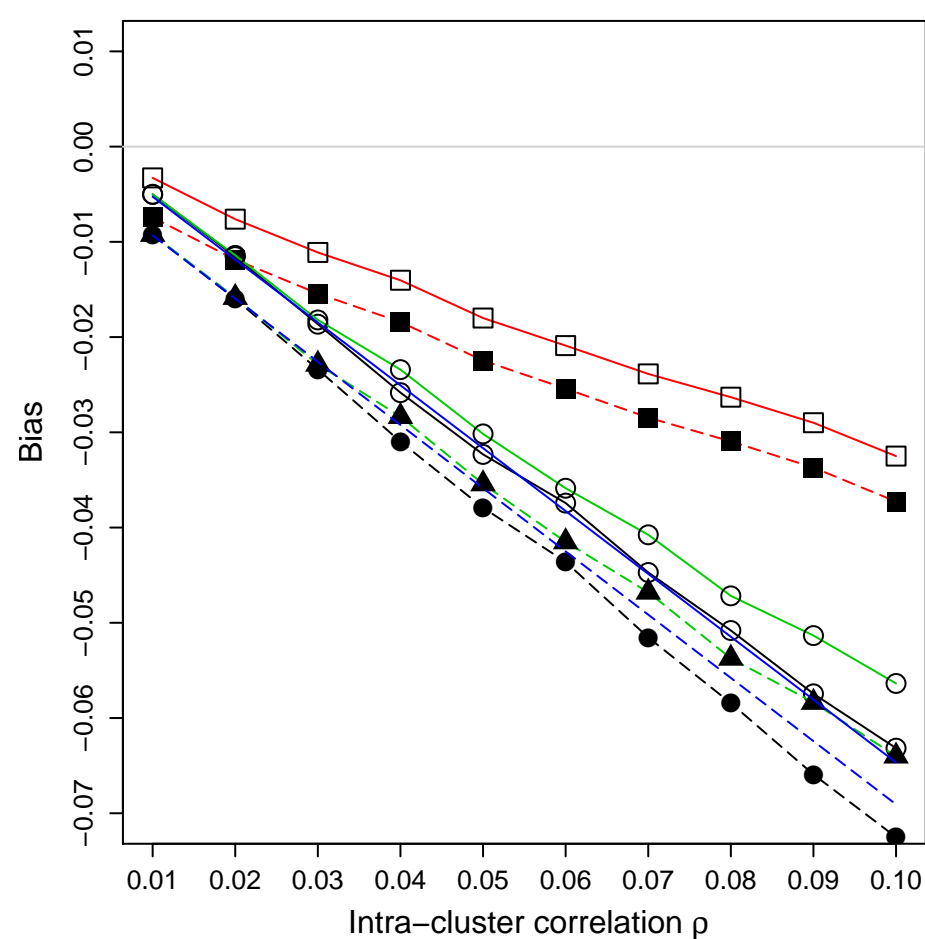

**n = 250 , K = 80**

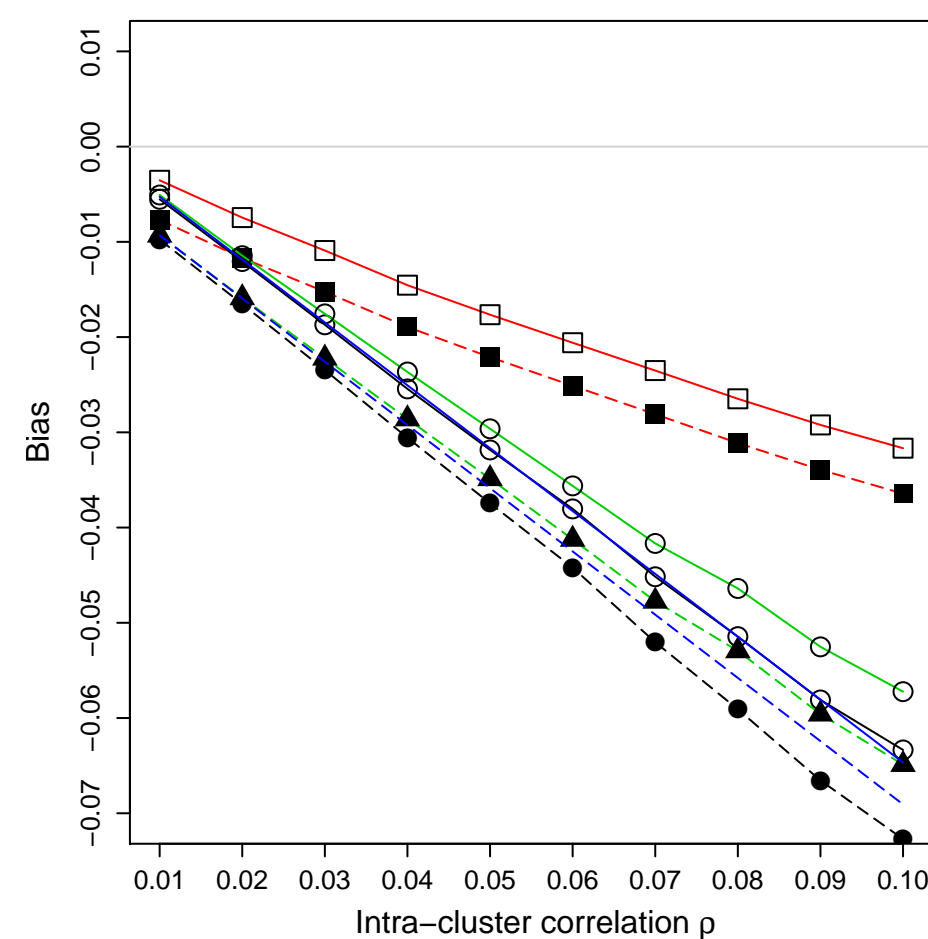

**n = 20 , K = 10**

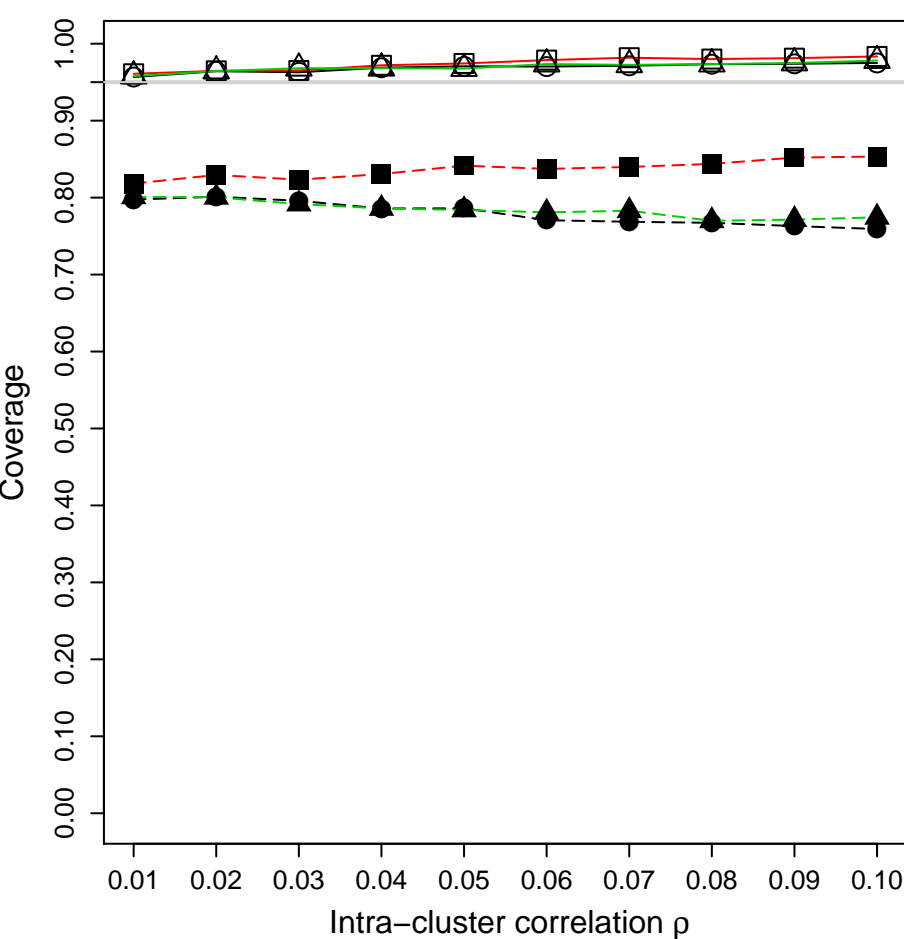

**n = 20 , K = 30**

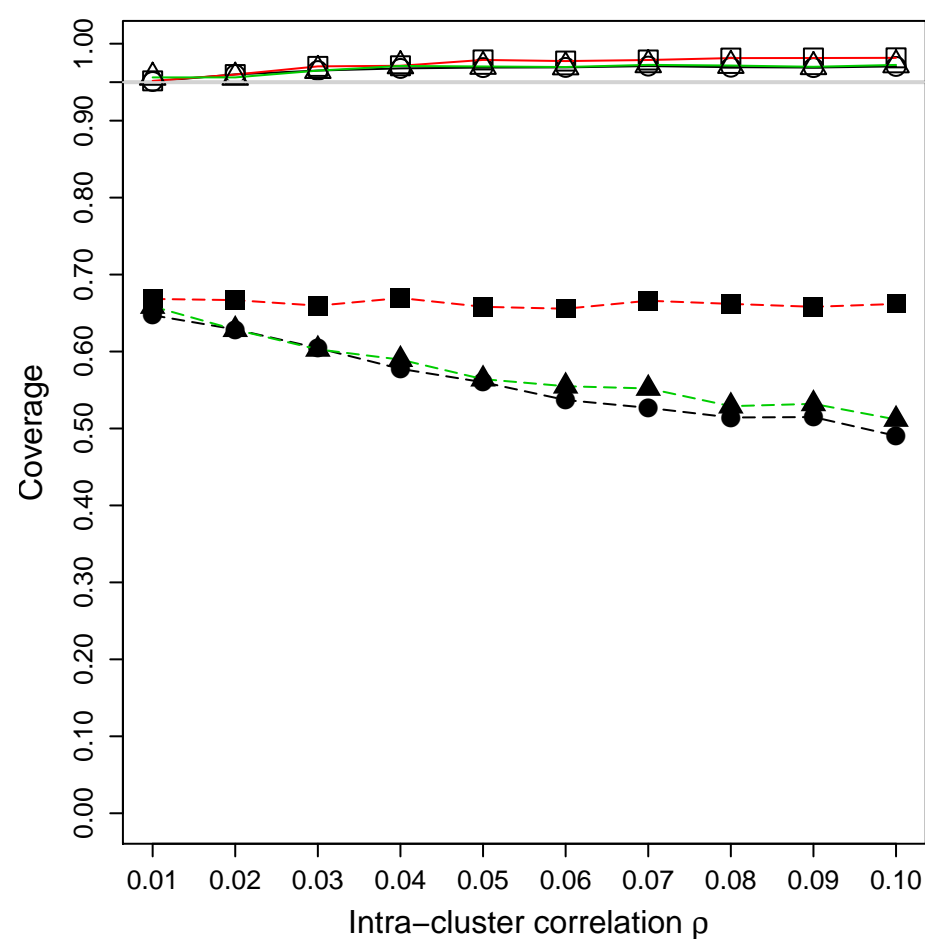

**n = 20 , K = 80**

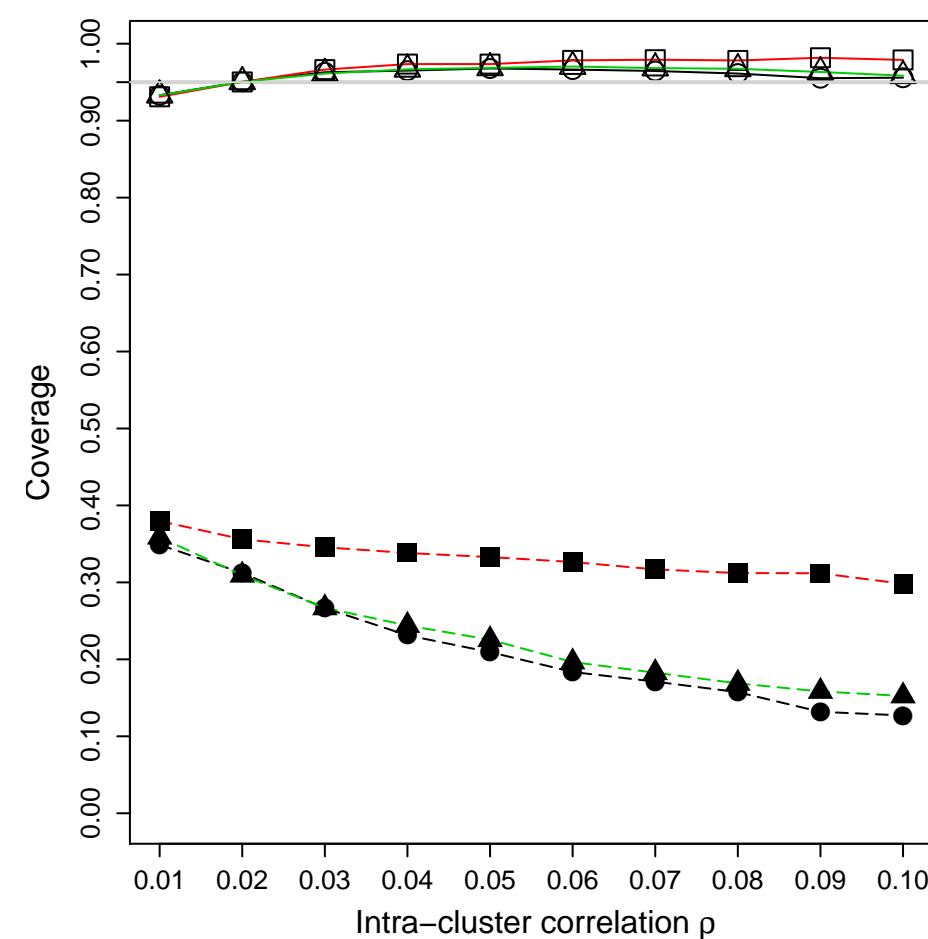

**n = 250 , K = 10**

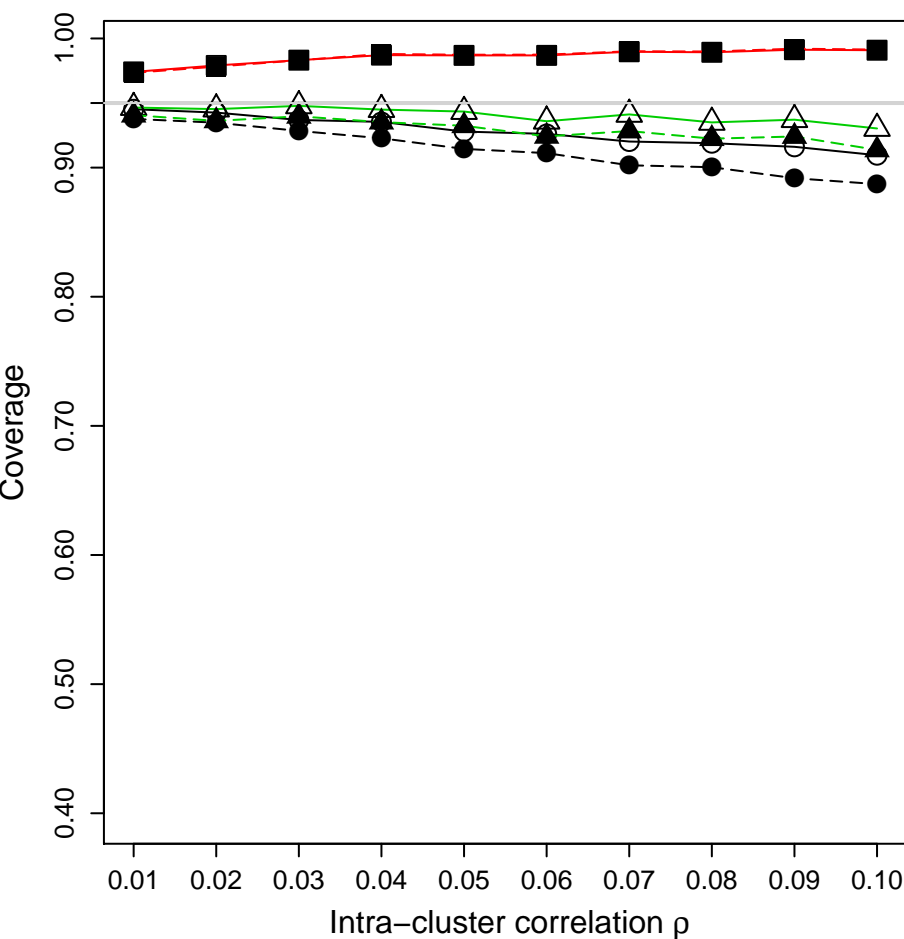

**n = 250 , K = 30**

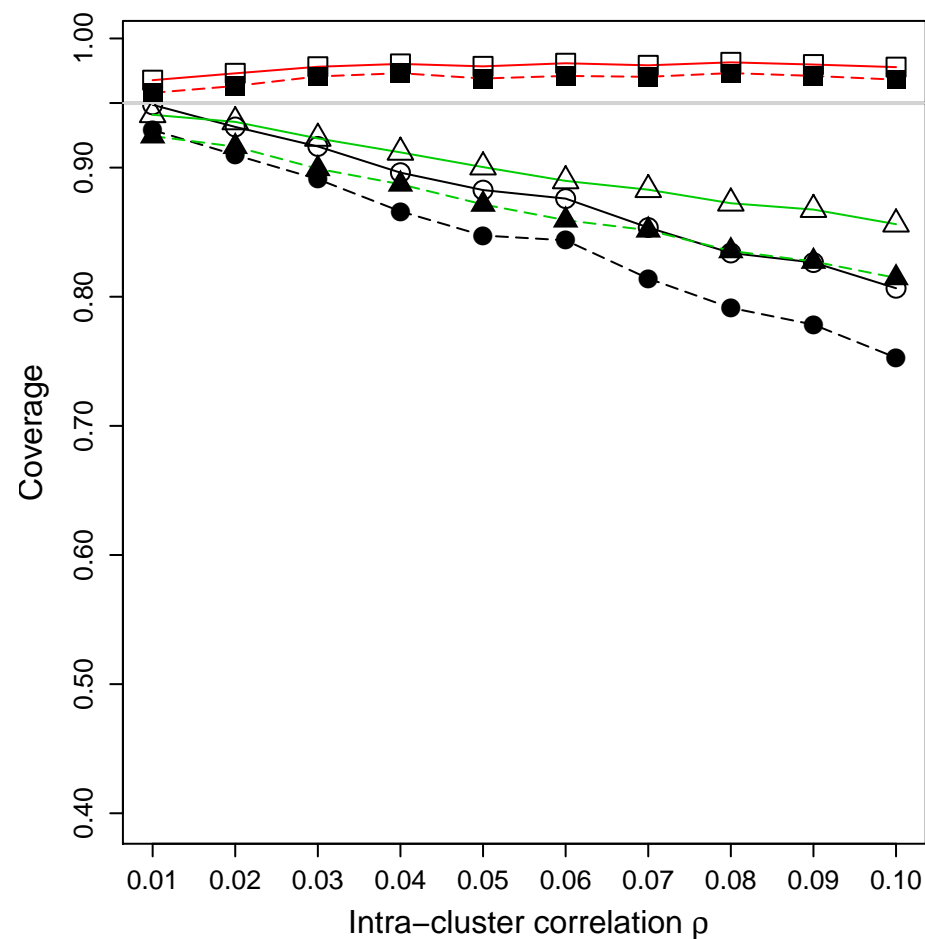

**n = 250 , K = 80**

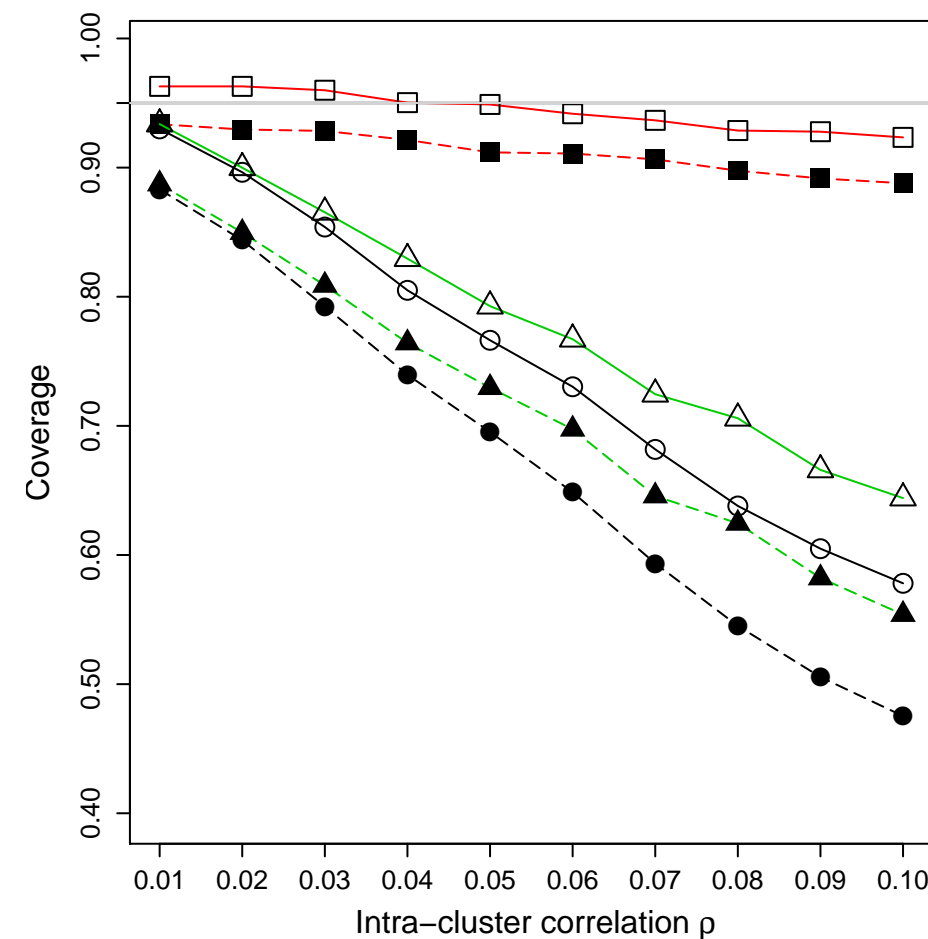

Supplement: Supplementary file 2 — Supporting Information [file BIMJ-58-896-s002.zip › README/FIGURE2/BiasAndCovPlotAsinBBandLDandNCp01KisNot1MA.pdf]

**n = 20 , K = 10**

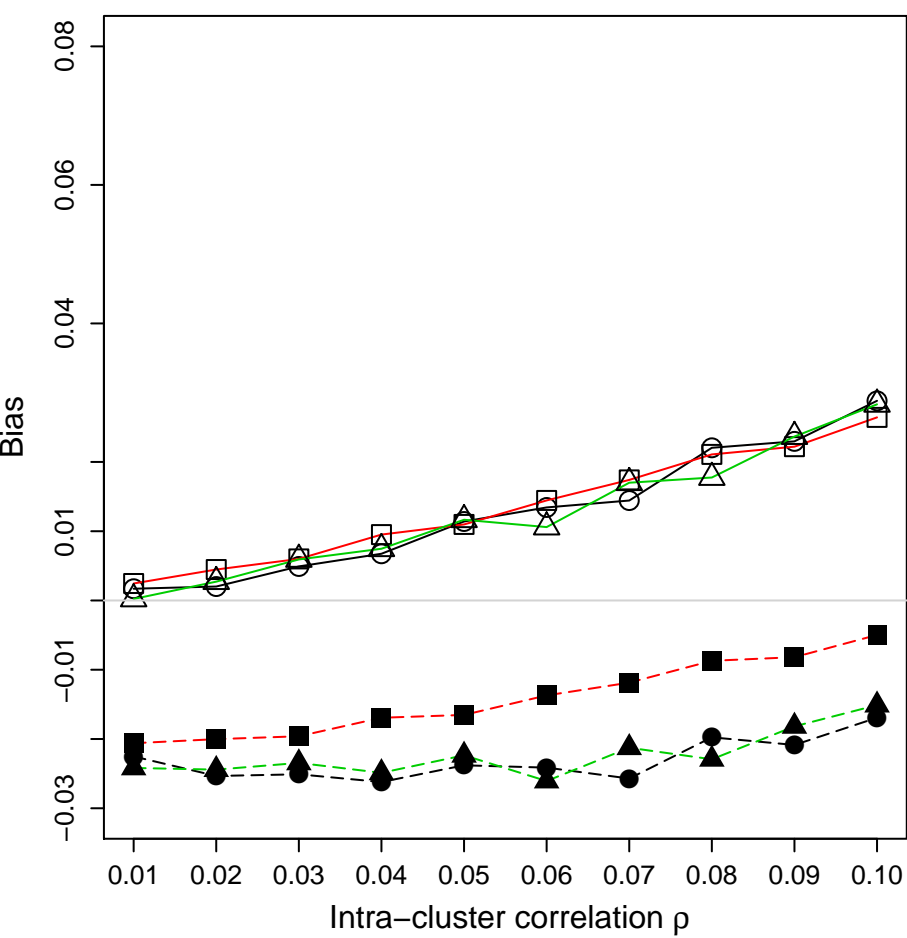

**n = 20 , K = 30**

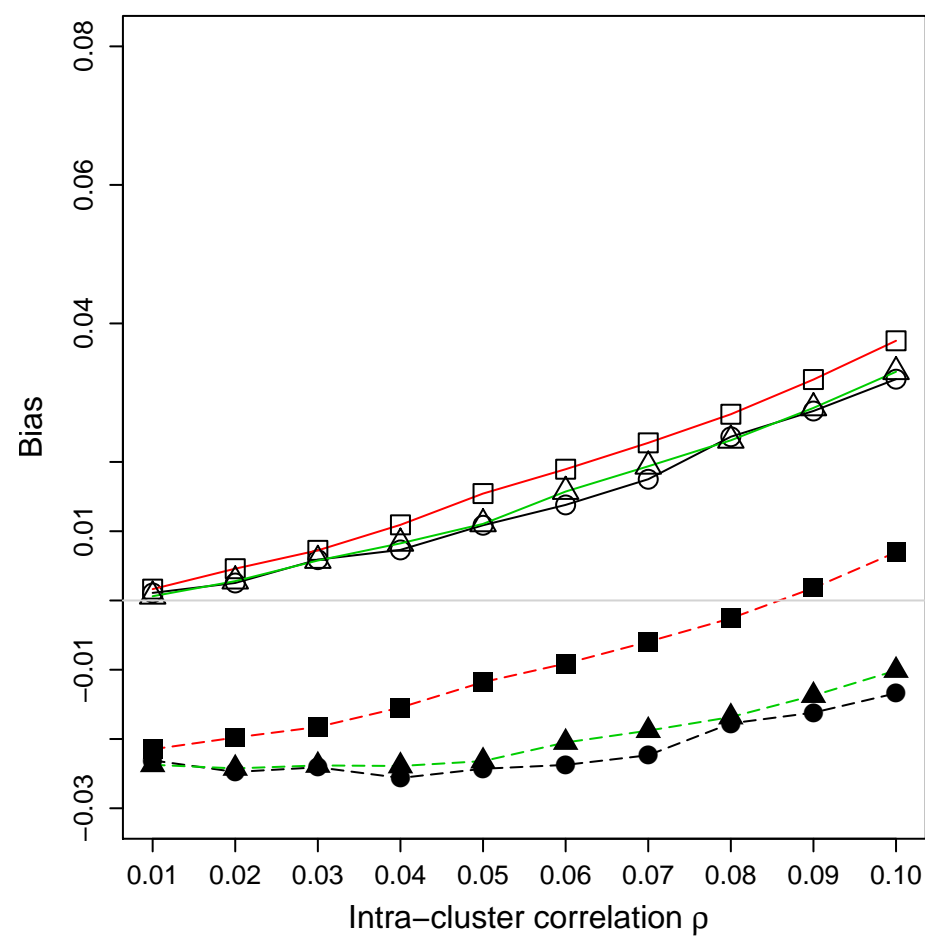

**n = 20 , K = 80**

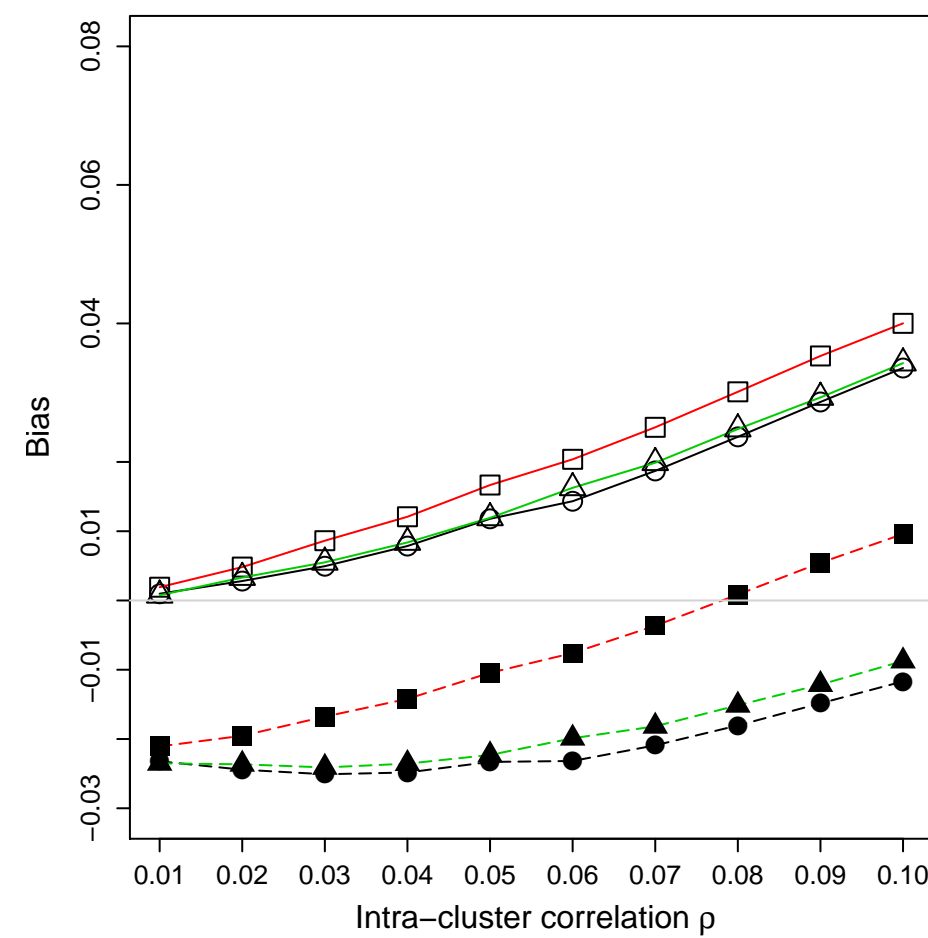

**n = 250 , K = 10**

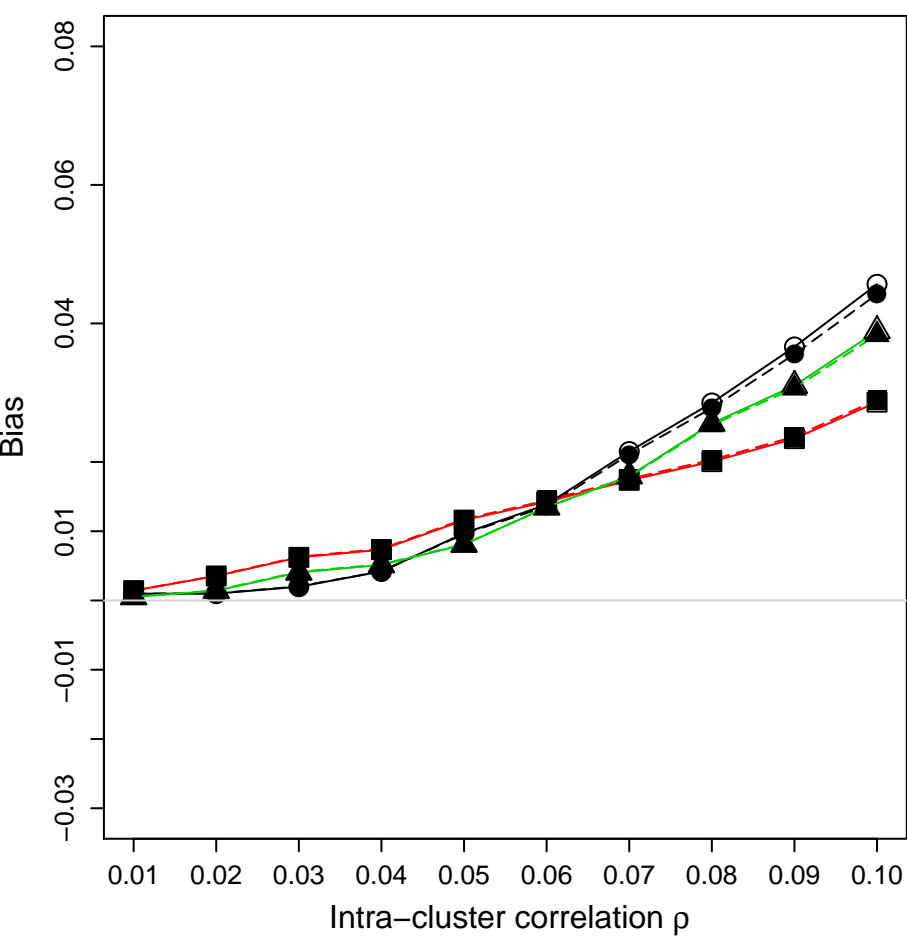

**n = 250 , K = 30**

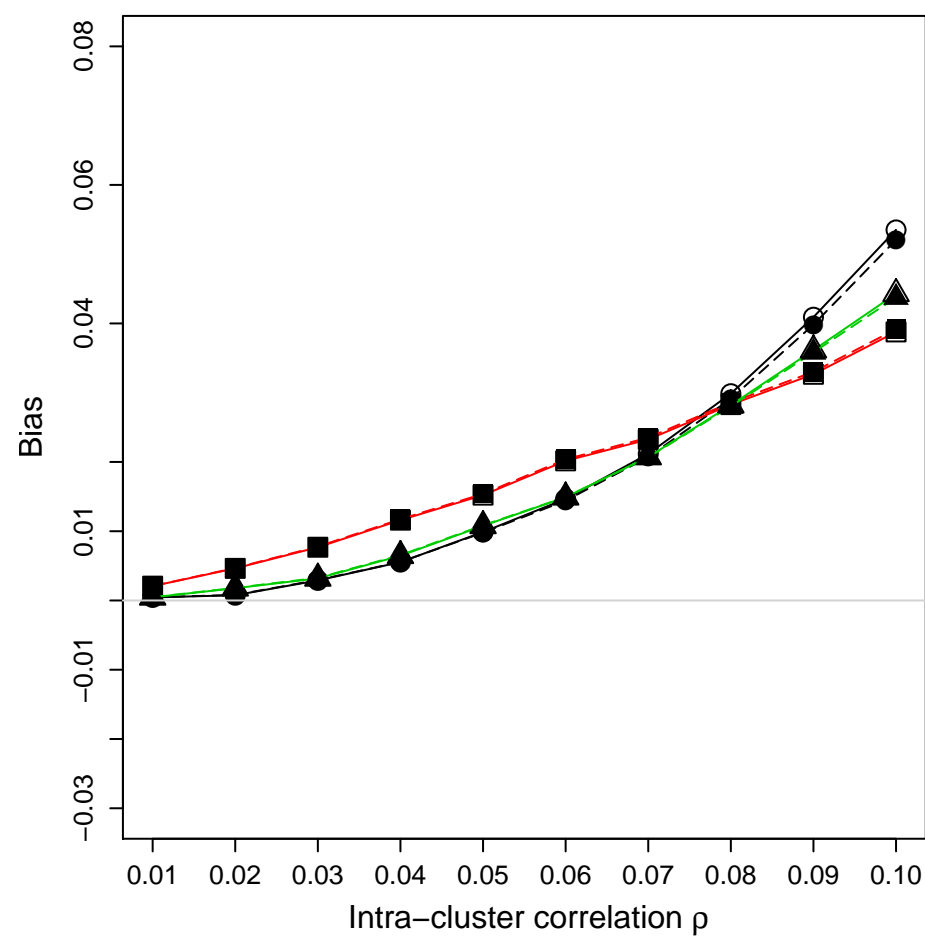

**n = 250 , K = 80**

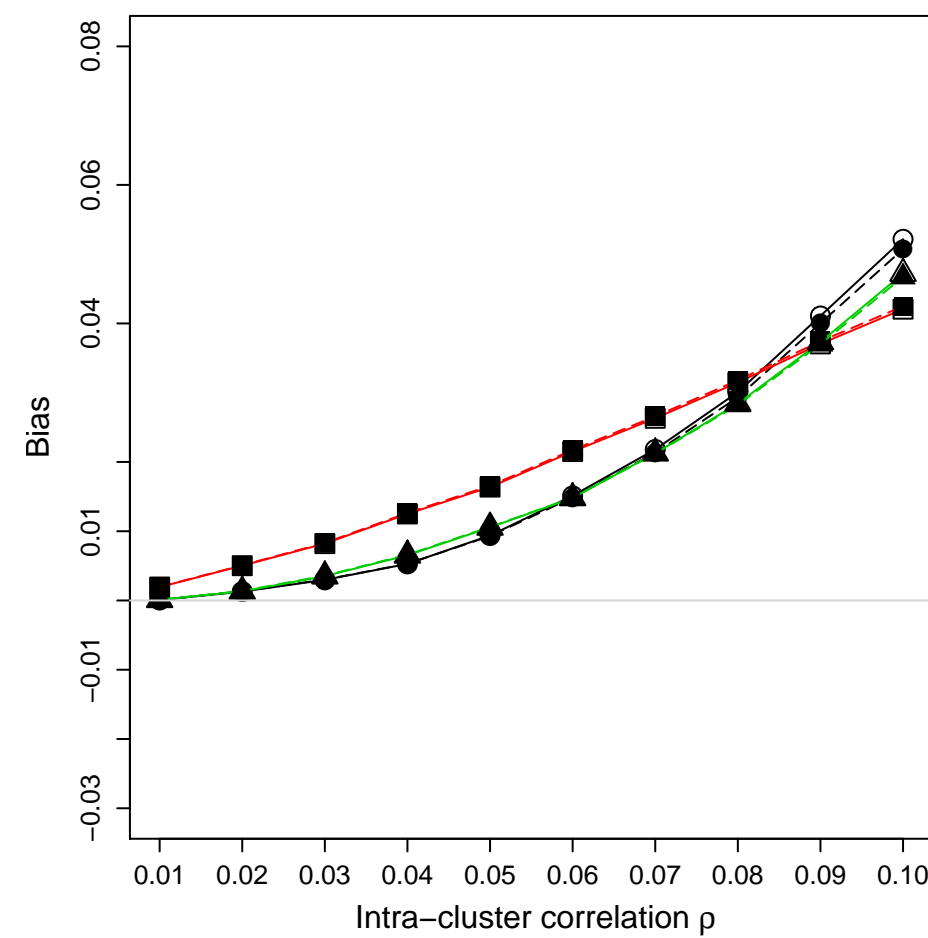

**n = 20 , K = 10**

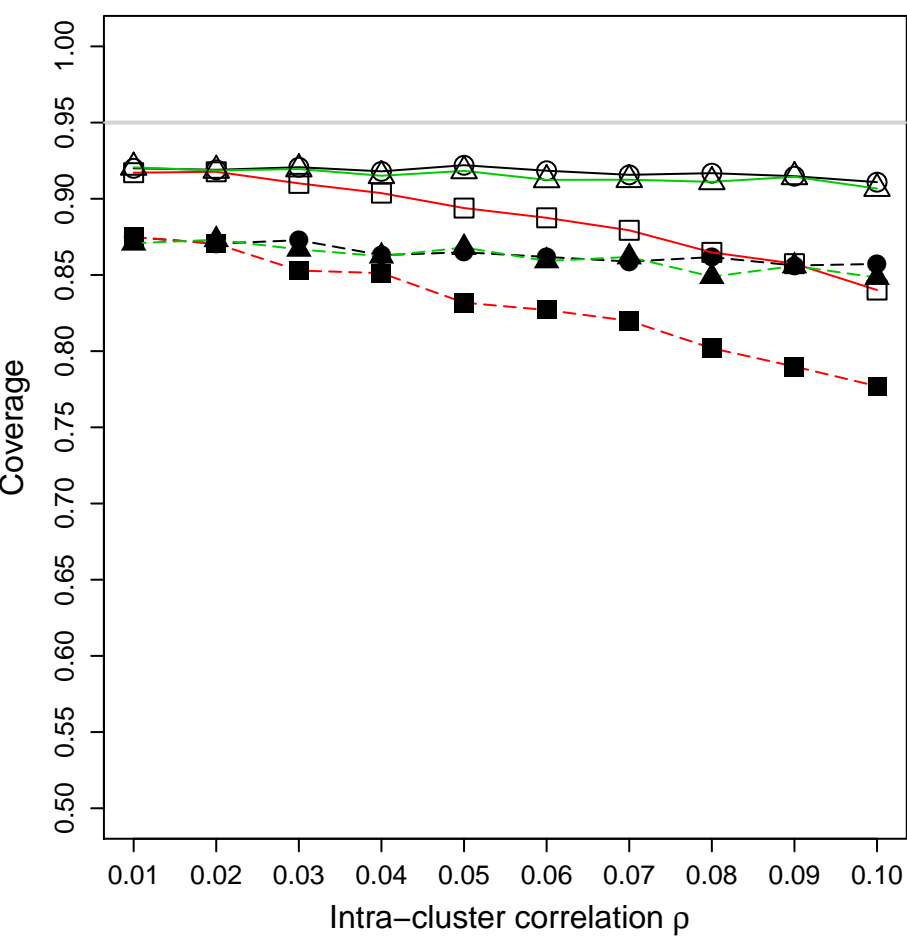

**n = 20 , K = 30**

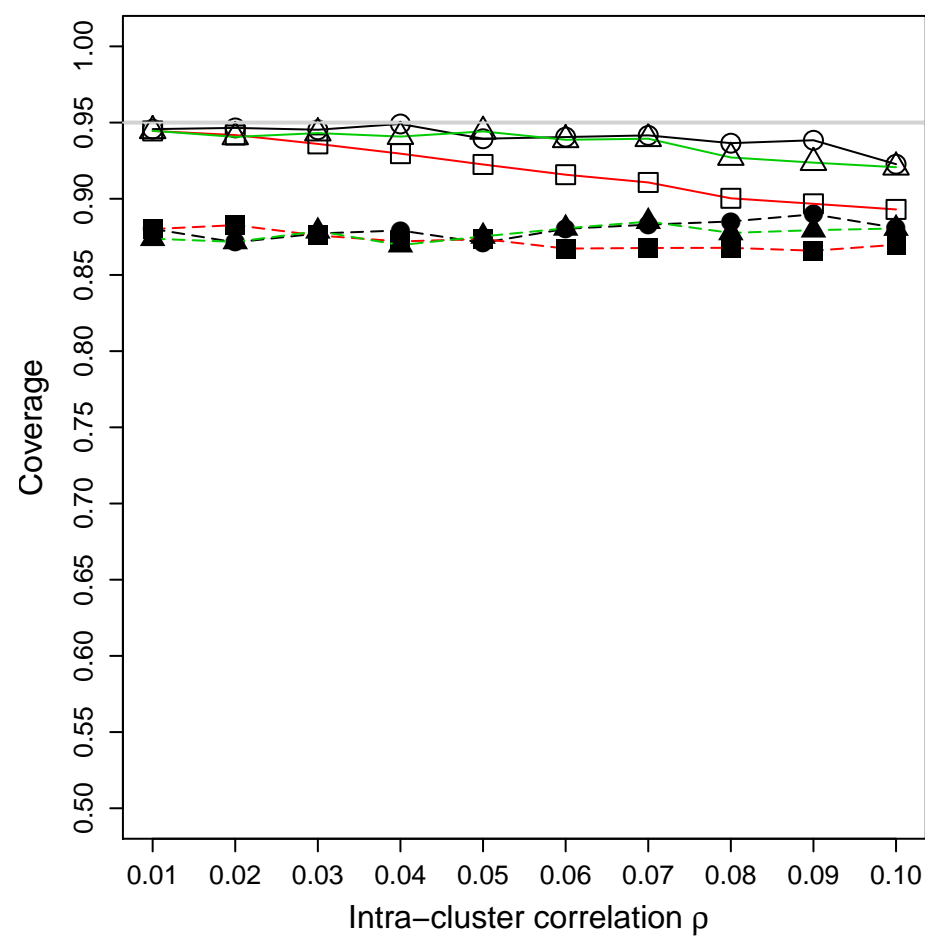

**n = 20 , K = 80**

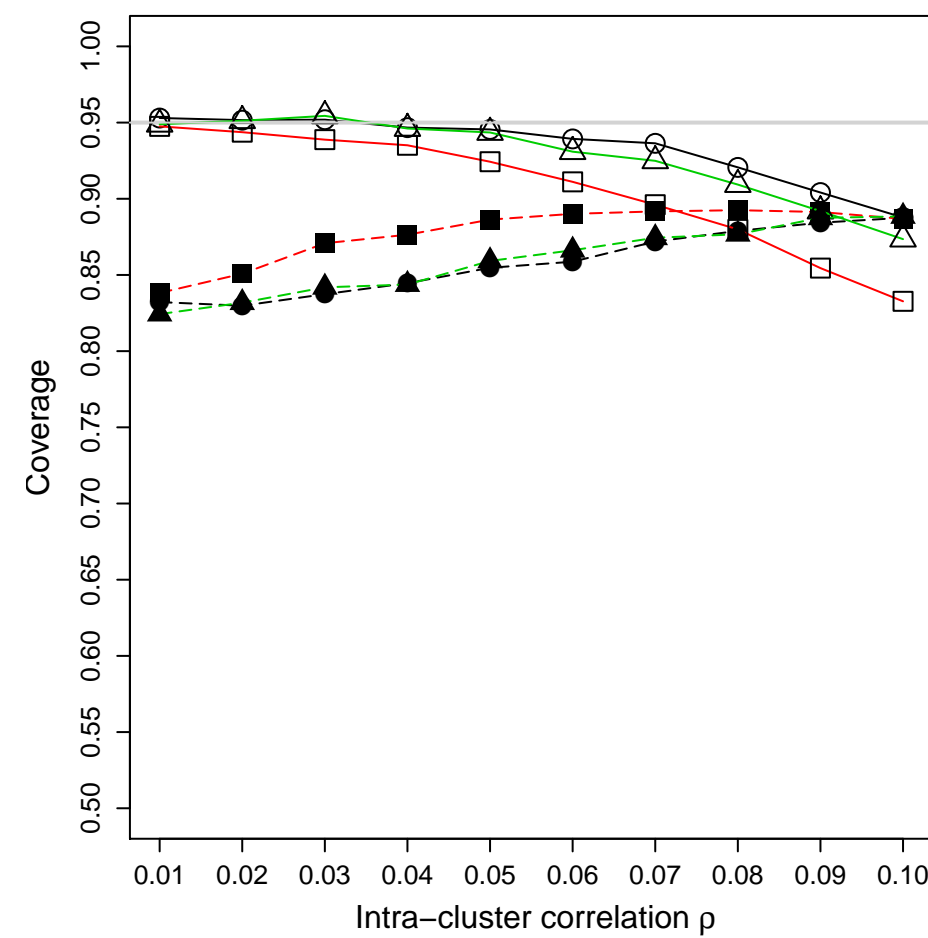

**n = 250 , K = 10**

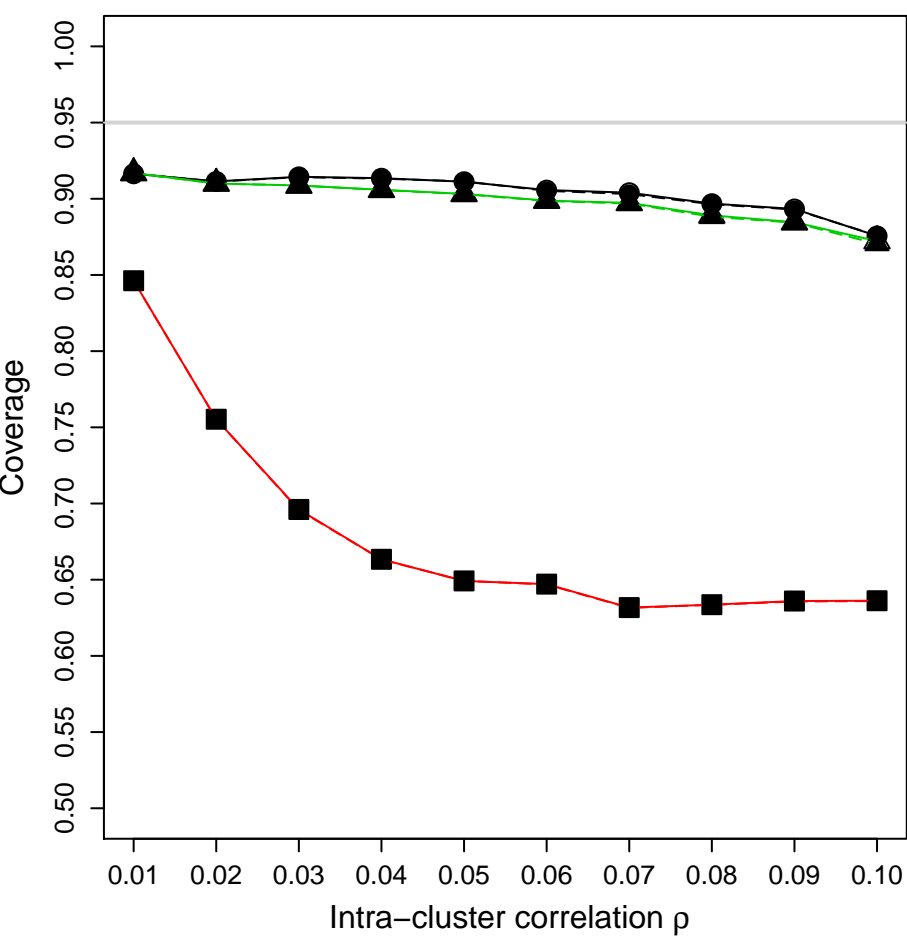

**n = 250 , K = 30**

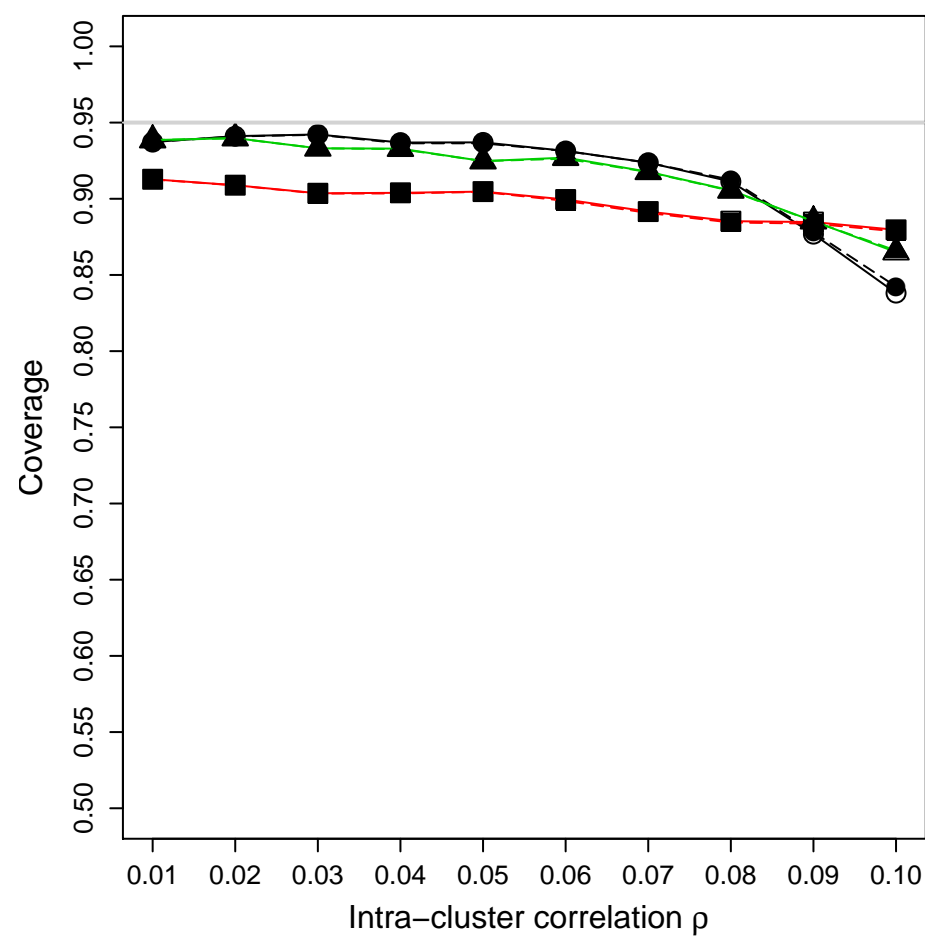

**n = 250 , K = 80**

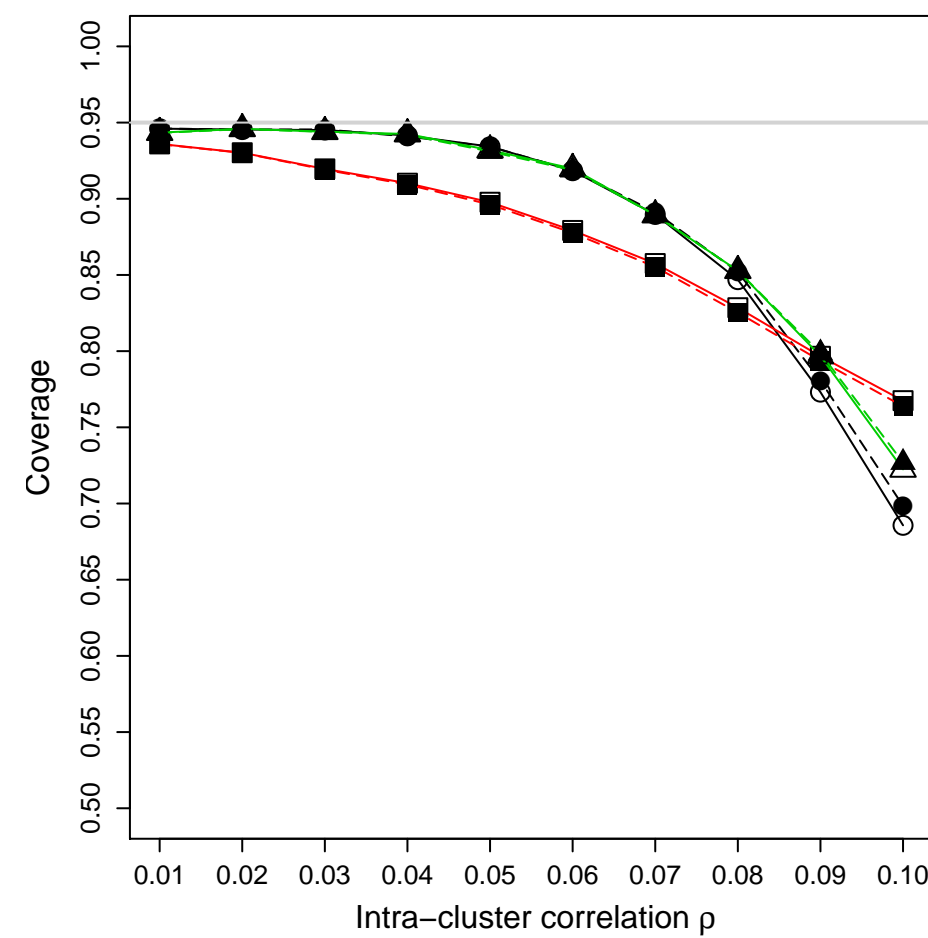

Supplement: Supplementary file 2 — Supporting Information [file BIMJ-58-896-s002.zip › README/FIGURE3/BiasAndCovPlotAsinBBandLDandNCp01KisNot1MAWithBiasCorrection.pdf]

**n = 20 , K = 10**

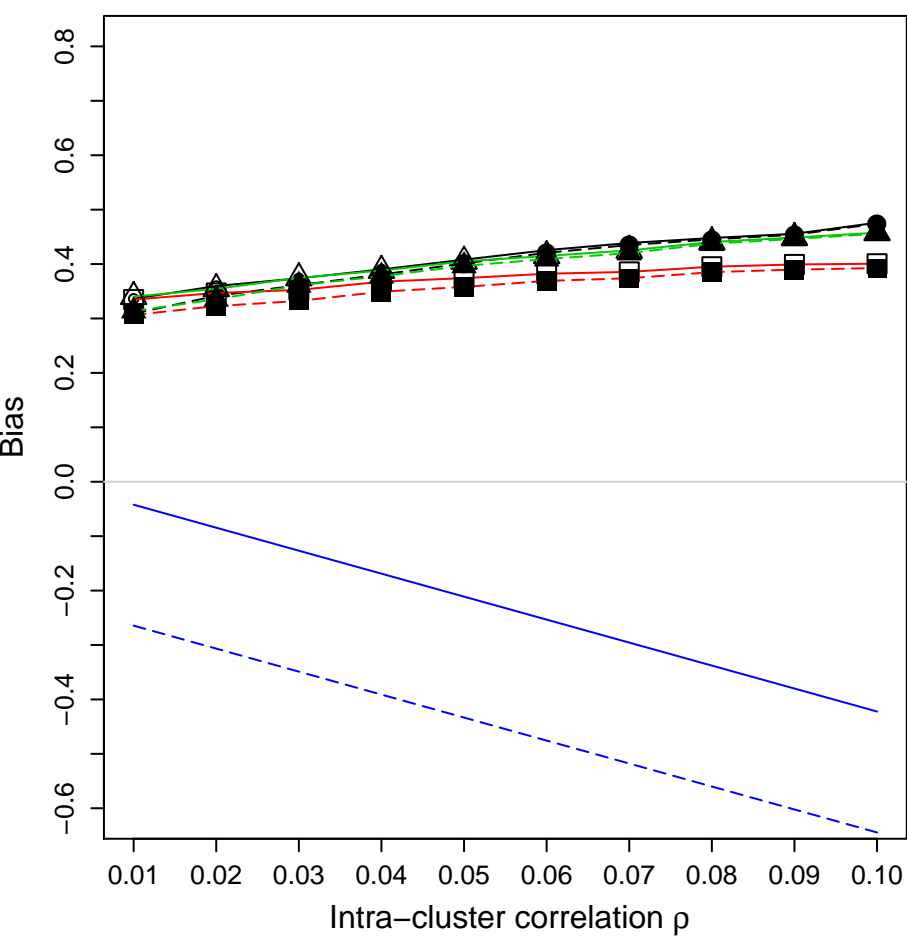

**n = 20 , K = 30**

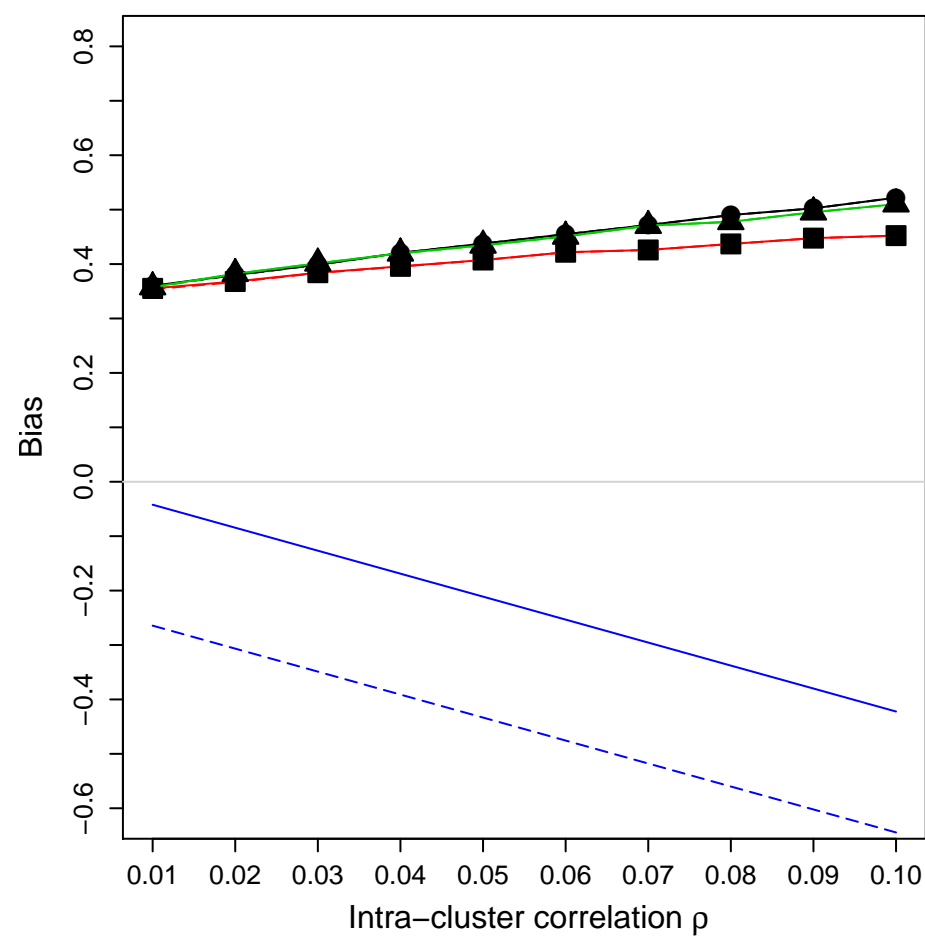

**n = 20 , K = 80**

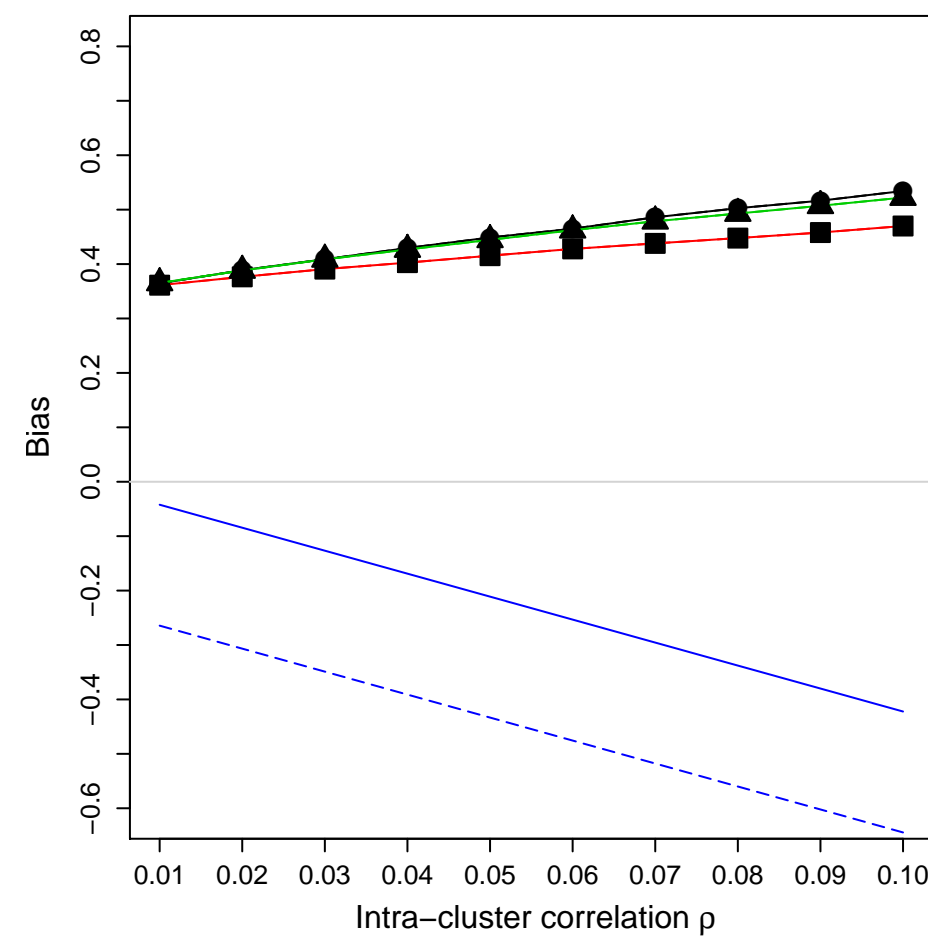

**n = 250 , K = 10**

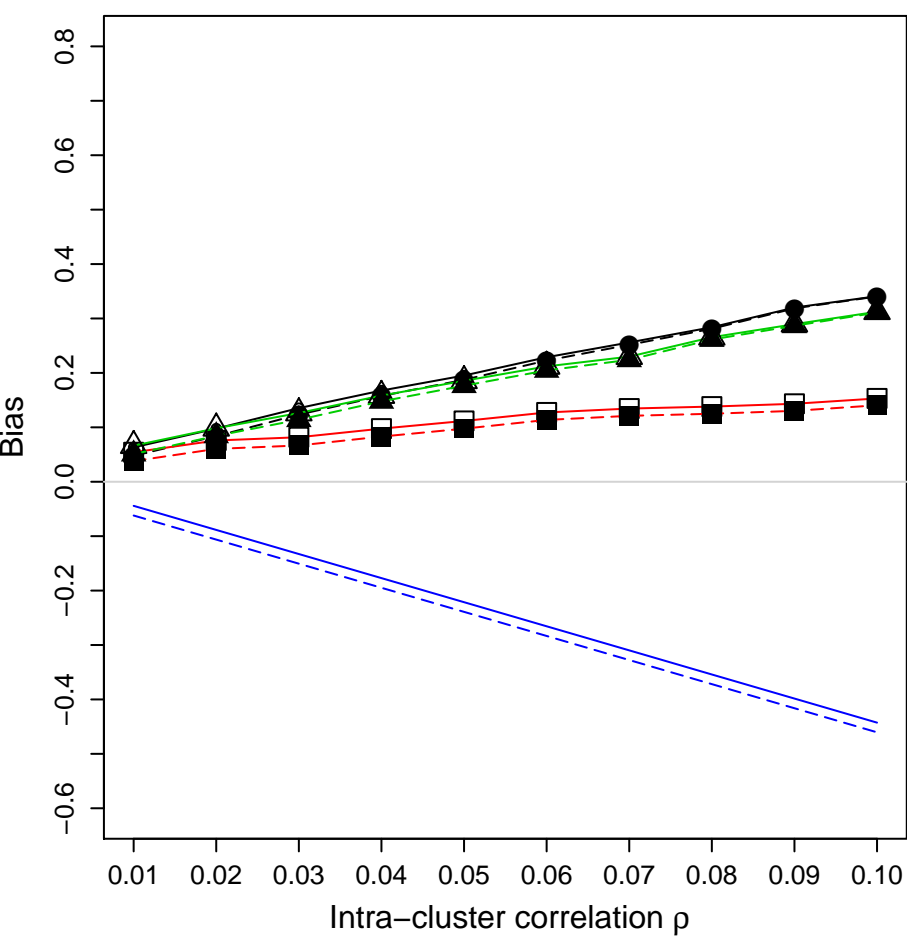

**n = 250 , K = 30**

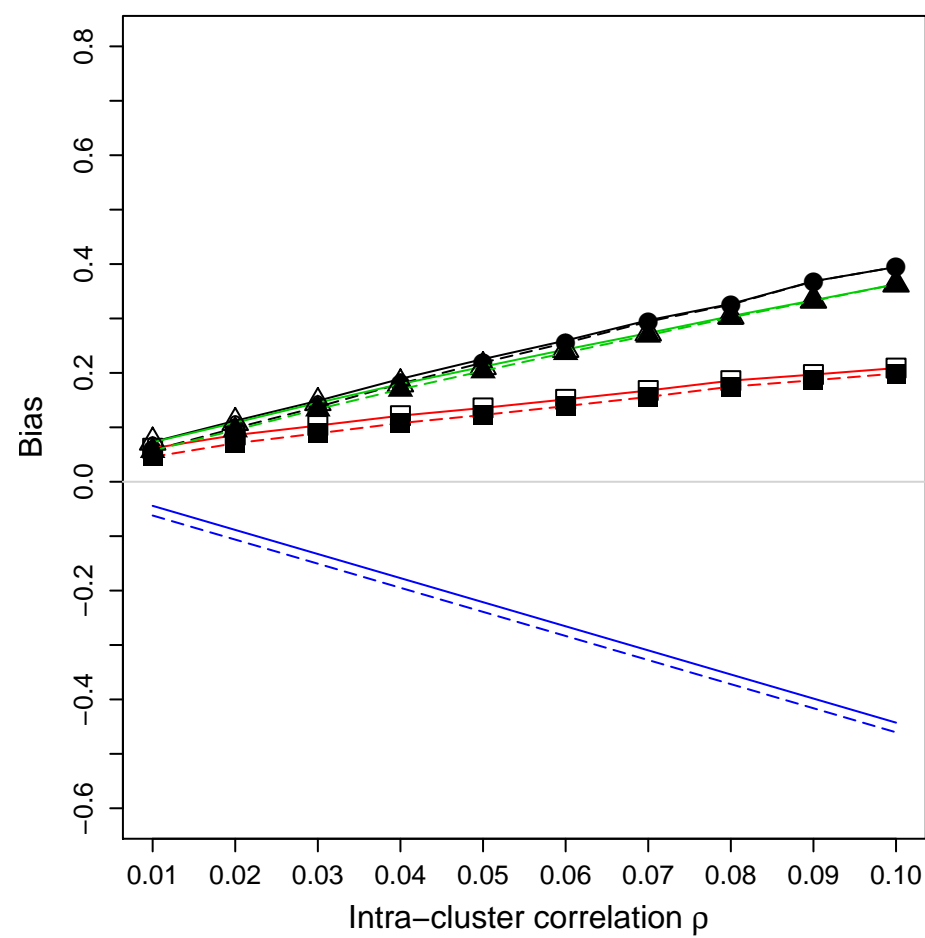

**n = 250 , K = 80**

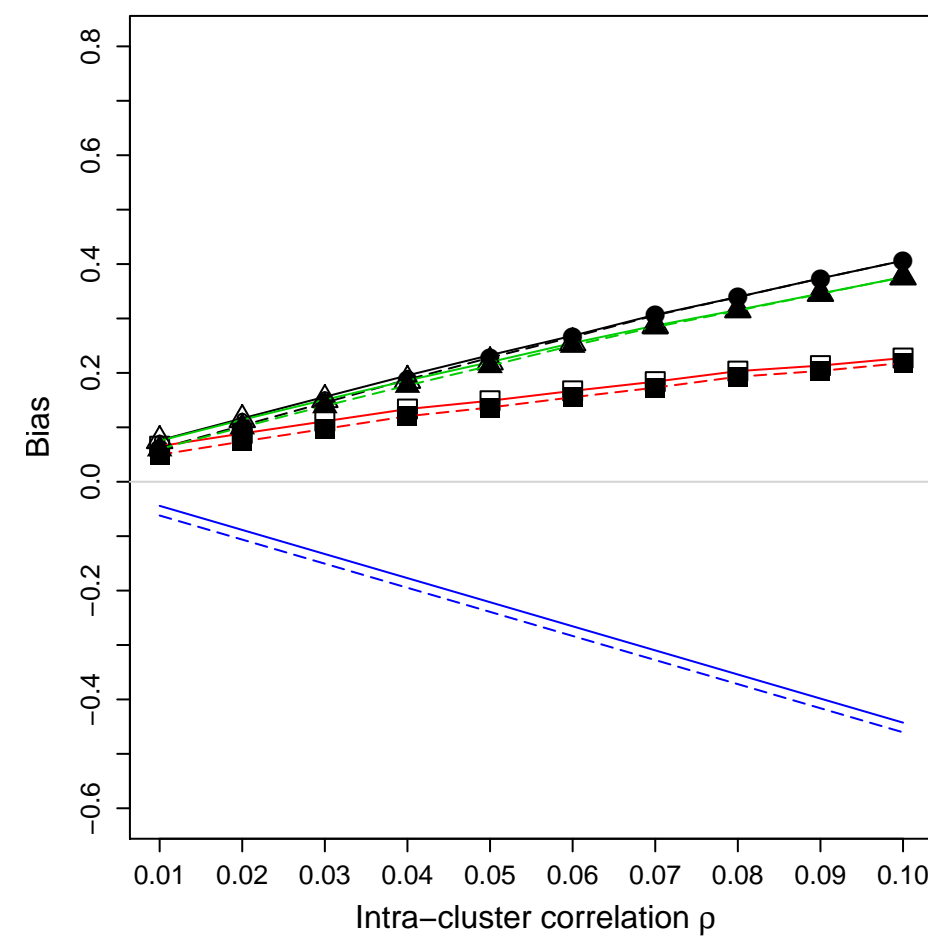

**n = 20 , k = 10**

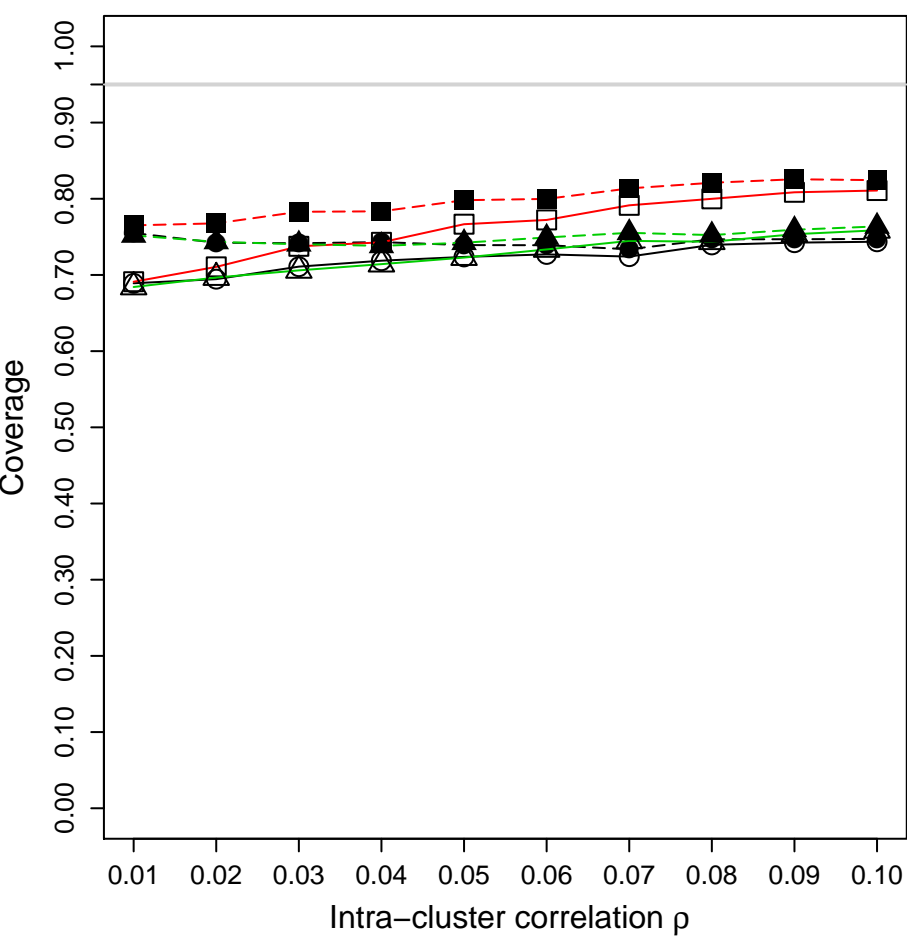

**n = 20 , k = 30**

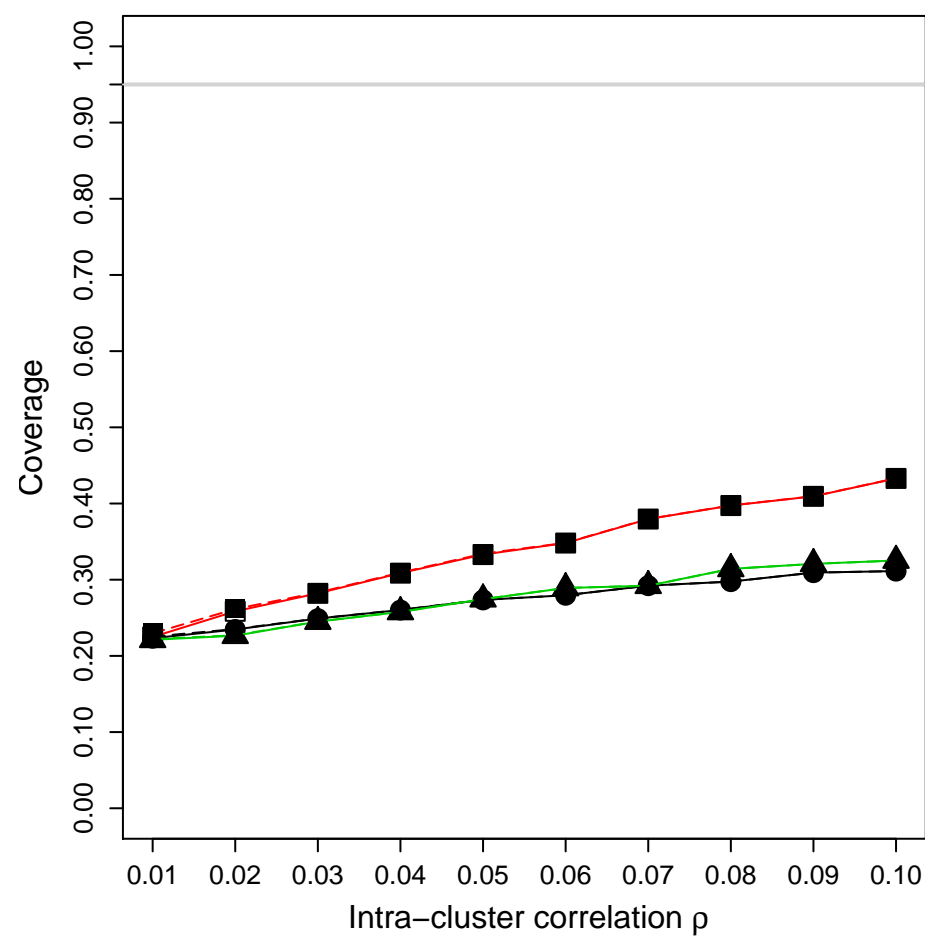

**n = 20 , k = 80**

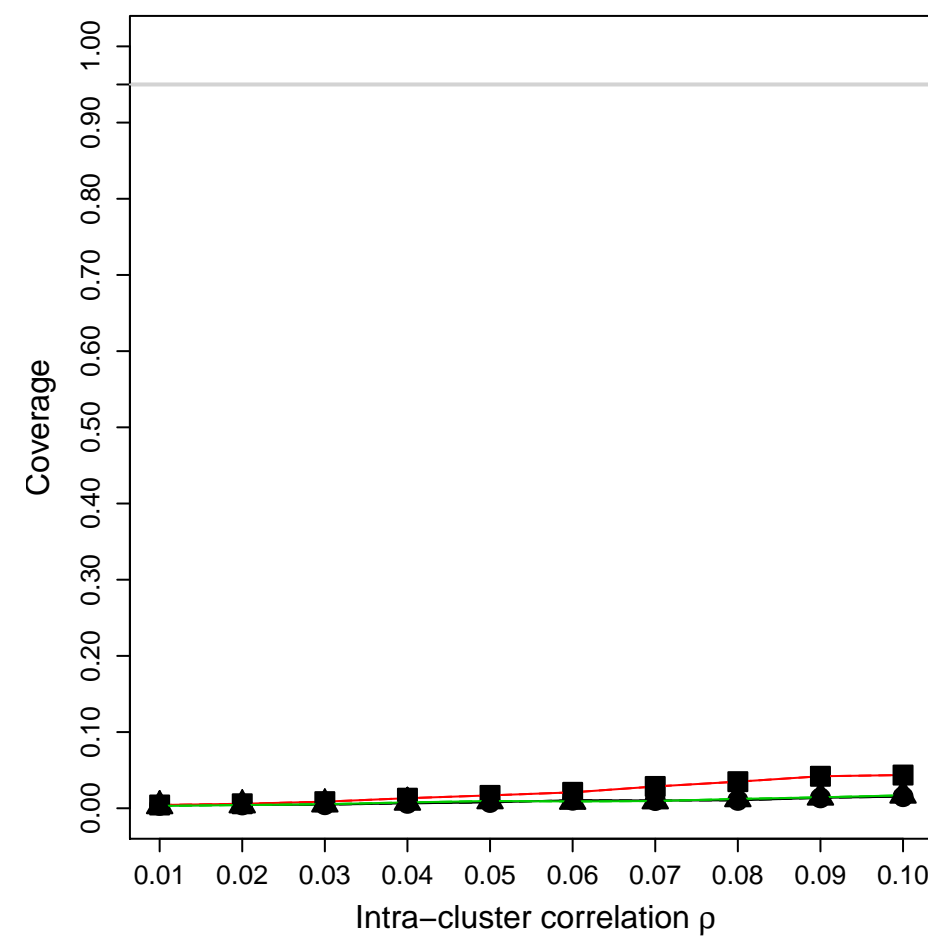

**n = 250 , k = 10**

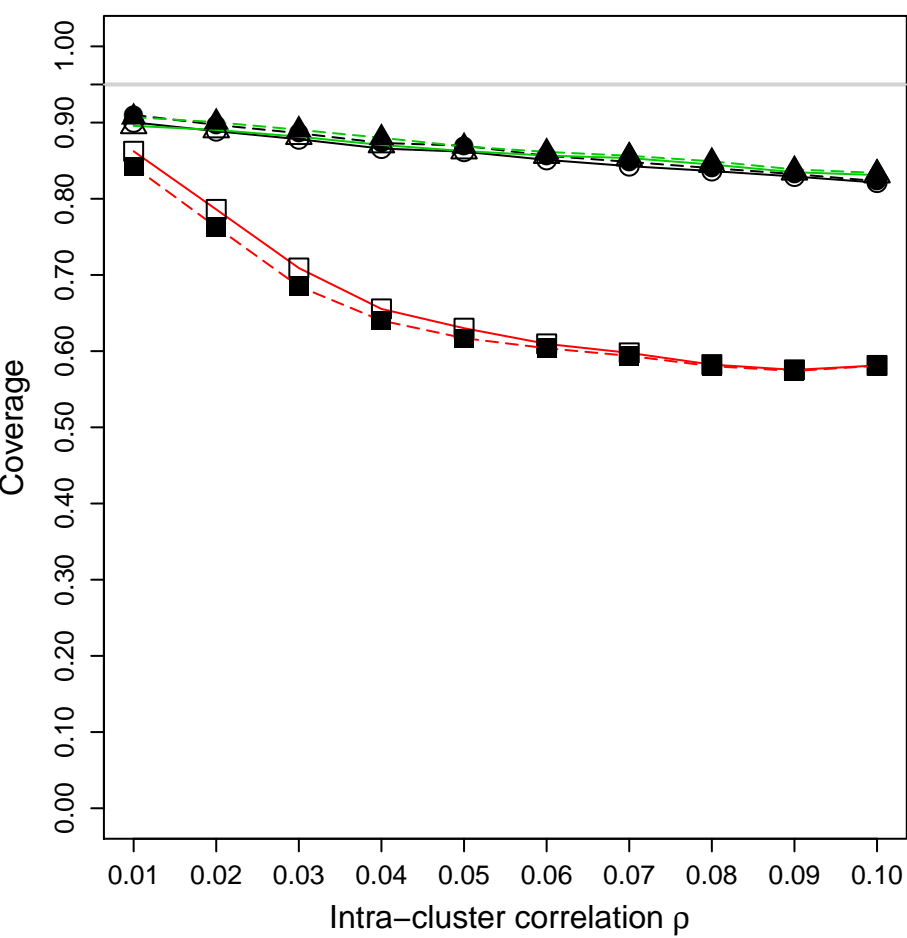

**n = 250 , k = 30**

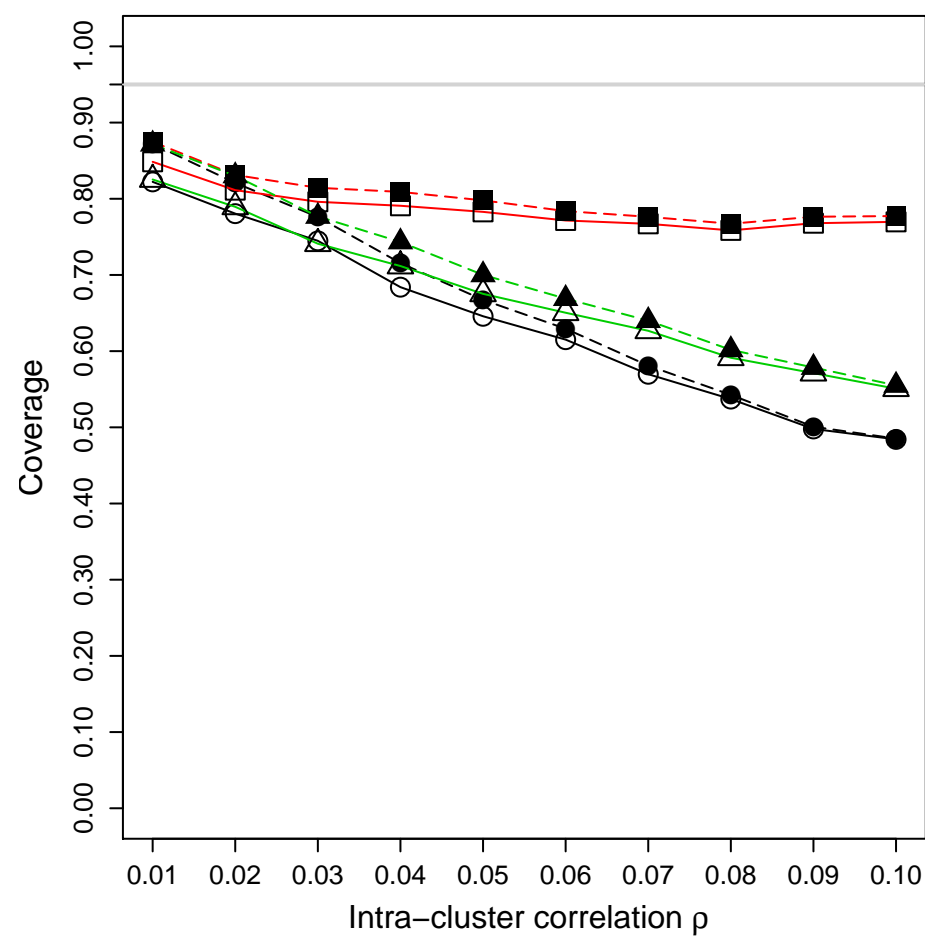

**n = 250 , k = 80**

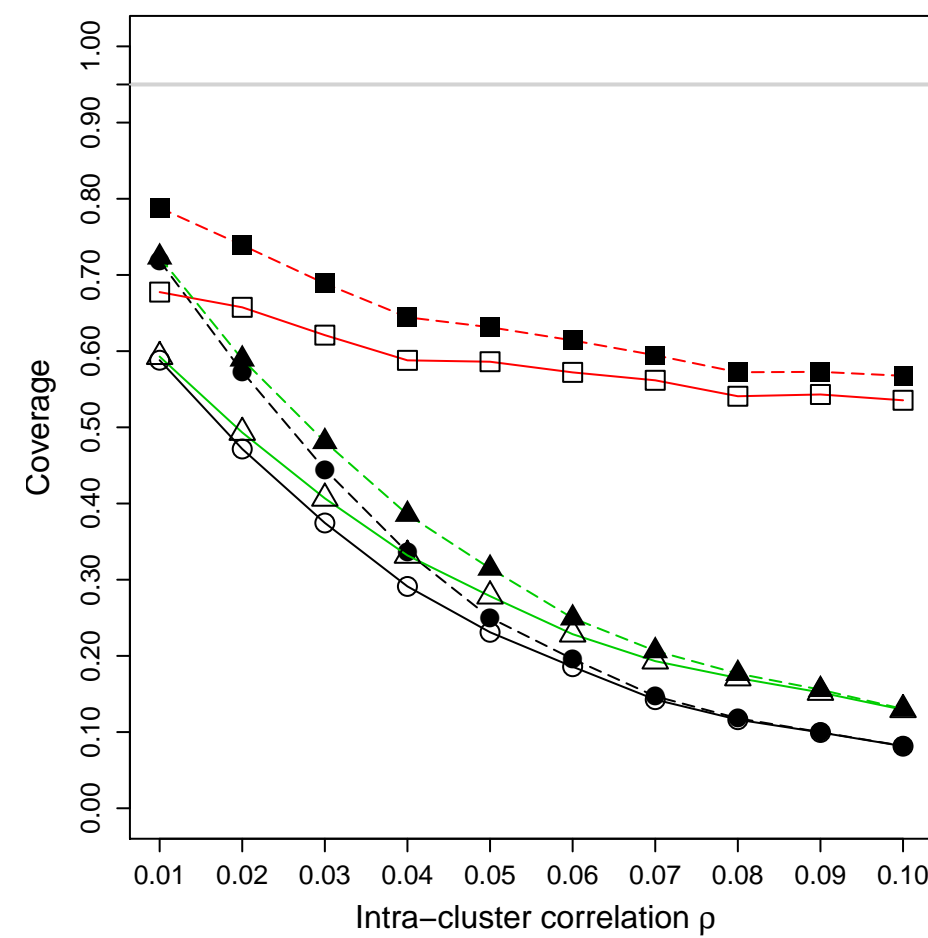

Supplement: Supplementary file 2 — Supporting Information [file BIMJ-58-896-s002.zip › README/FIGURE4/BiasAndCovPlotALogOddsBBandLDandNCp01KisNot1MA.pdf]
